# USP33 promotes pancreatic cancer malignant phenotype through the regulation of TGFBR2/TGFβ signaling pathway

**DOI:** 10.1038/s41419-023-05871-4

**Published:** 2023-06-15

**Authors:** Xinyuan Liu, Jian Xu, Bingbing shen, Jichuan Xu, Jianxin Jiang

**Affiliations:** Department of Hepatobiliary Surgery, Renmin Hospital of Wuhan University, Wuhan, 430060 China

**Keywords:** Pancreatic cancer, Tumour biomarkers

## Abstract

Pancreatic cancer (PC) ranked fourth among cancer-related death worldwide with a survival rate less than 5%. The abnormal proliferation and distant metastasis are major obstacles for the diagnosis and treatment of pancreatic cancer, therefore, it is urgent for researchers to uncover the molecular mechanisms underlying the PC proliferation and metastasis. In current study, we found that USP33, a member of deubiquitinating enzyme family, was upregulated among PC samples and cells, meanwhile, the high expression of USP33 correlated with poor prognosis of patients. Function experiments revealed that USP33 overexpression promoted the proliferation, migration and invasion of PC cells while the inhibition of USP33 expression in PC cells exhibited the opposite effect. The mass spectrum and luciferase complementation assay screened TGFBR2 as the potential binding protein of USP33. Mechanistically, USP33 triggered the deubiquitination of TGFBR2 and prevented its degradation by lysosome, therefore promoted TGFBR2 accumulation in cell membrane and eventually contributed to the sustained activation of TGF-β signaling. Moreover, our results revealed that the activation of TGF-β targeted gene ZEB1 promoted the transcription of USP33. In conclusion, our study found that USP33 contributed to the proliferation and metastasis of pancreatic cancer through a positive feedback loop with TGF-β signaling pathway. Moreover, this study suggested that USP33 may serve as a potential prognostic and therapeutic target in PC.

## Introduction

Previous studies demonstrated that pancreatic cancer (PC) was the 4th leading cause of cancer-related death worldwide [1]. Approximately 90% of PC patients were diagnosed at advanced stages with over 50% of systemic metastases which made 80% of patients unsuitable for surgical intervention [2, 3]. Moreover, the lack of effective therapeutic targets limited the diagnosis and treatment of PC. Recent years, researchers have found various potential molecules participated in the proliferation and metastasis of PC, such as KRAS and P53 [4, 5]. However, the specific molecular mechanisms that promoted the metastatic spread of PC was incompletely elucidated.

TGF-β signaling participated in the regulation of embryonic development, immune responses, the tumorigenesis and tumor metastasis [6, 7]. Moreover, TGF-β signaling played dual roles in the progression of various kinds of tumors [8]. At early stage of cancer TGF-β signaling functioned as a tumor suppressor while in advanced stage it performed as a promoter. PC had the highest incidence of TGF-β pathway mutations among cancers but the exact mechanism by which TGF-β pathway mediated the progression of PC remained uncleared. The TGF-β signal transduction required the activation of TGFBR2 and TGFBR1, which leads to the nuclear translocation of SMAD2/SMAD3 and eventually activate various kinds of transcription factors thereby regulate the malignant phenotype of PC. The whole TGF-β signaling relies mostly on the activation of TGFBRs, and previous study reported that the stability of TGFBRs in the plasma membrane is crucial for later transduction of TGF-β signaling [9, 10]. The accumulation of TGFBRs in membrane relied on the recycling endosome which carried proteins for targeted delivery [11–13]. Though mounting evidence suggested that TGFBR2 played a vital role in the malignant progression of PC, its underlying mechanism remained unclear.

As an important posttranslational modification, the ubiquitination of proteins participating in multiple biological processes [14, 15]. The balance between ubiquitination and deubiquitylation was controlled by multiple ubiquitinases and deubiquitinating enzymes [16, 17]. Previous studies had revealed that various deubiquitinating enzymes regulated the malignance of cancers. For instance, it was reported that OTUD7B functioned as a tumor suppressor by antagonizing the LCL161-induced lung cancer cell invasion and migration [18]. As a member of deubiquitinating enzyme, USP33 had been proved to be involved in serious of diseases. Previous study suggested that USP33 enhanced the resistance of prostate cells to docetaxel-induced apoptosis through deubiquitinating and stabilizing the DUSP1 protein to impair JNK signaling transduction [19]. In addition, the mitochondria localized USP33 could deubiquitinated the K6, K11, K48 and K63-linked ubiquitin conjugates on PRKN, therefore, USP33 served as a PRKN deubiquitinating enzymes to antagonize its pro-mitophagy effect [20]. USP33 could also serve as the deubiquitinating enzyme of HIF-1A protein and promote the stability of HIF-1A protein to control the HIF-1A signaling pathway in cancers [21]. Though there have been numbers of reports discussing the role of USP33 in cancers, the function of USP33 in pancreatic cancer has not been clearly elucidated.

In this study, we found for the first time that USP33 was upregulated in PC tissues and cells. The overexpression of USP33 accelerated the proliferation, invasion and metastasis of PC in vitro and in vivo. Mechanistically, USP33 deubiquitinated and stabilized the TGFBR2 protein in PC cells and therefore enhanced the signaling of TGF-β pathway, USP33 removed the K63-linked ubiquitin conjugates from TGFBR2 and prevented its degradation by lysosome, meanwhile, USP33 promoted the recycling of TGFBR2 to cell membrane and eventually enhanced the signaling in TGF-β pathway. Moreover, our research found that TGF-β signaling targeted gene ZEB1 activated the transcription of USP33, the positive loop between USP33 and TGF-β pathway eventually accelerated the progression of PC. In conclusion, our finding indicated that USP33 could serve as a potential therapeutic target for PC diagnosis and treatment.

## Materials and methods

### Cell culture and transfection

Immortalized human pancreatic ductal epithelial cells (HPDE) and human pancreatic cancer cells AsPC-1, BxPC-3 were cultured in DMEM medium (Solarbio Science & Technology Co., Beijing, China) contained 10% fetal bovine serum (FBS, Cegrogen, Stadtallendorf, Germany), 100 U/ml penicillin and 100 U/ml streptomycin (Beyotime Biotechnology), human embryonic kidney (HEK293T) cells and pancreatic cancer cells MIA PaCa-2, PANC-1 and SW-1990 were cultured in RPMI 1640 medium (Solarbio Science & Technology Co., Beijing, China) contained 10% fetal bovine serum, 100 U/ml penicillin and 100 U/ml streptomycin. All cells were cultured in a 5% CO2 atmosphere at 37 °C.

The indicated shRNAs or the corresponding control (NC) RNAs were constructed into the lentiviral vector pENTR/H1/TO (ThermoFisher) and transfected into the indicated cell lines. Lipofectamine 2000 (ThermoFisher Scientific) was used for shRNA transfection. The sequence of shRNAs were showed in the supplementary table 1. The full length or truncated sequences of USP33 and TGFBR2 were coloned into pHAGE-HA or pHAGE-Flag vectors, the USP33^C194A^ was constructed by PCR mutagenesis of the pHAGE-HA plasmid. The MYC-tagged WT, K6R, K11R, K27R, K29R, K33R, K48R and K63R mutant plasmids were purchased from GENECHEM. The Lipofectamine lipo3000 reagent (Lipofectamine lipo3000; Invitrogen) was used for the instantaneous transfection of plasmid DNA.

### PCR

The RNA-easy Isolation Reagent (Vazyme, China) was used to extract RNA from all samples, in briefly, the samples were digested and lysed by RNA-easy Isolation Reagent, then the samples were centrifuged at 12,000 rpm and the total RNA was precipitated by isopropanol/ethanol, then the HiScript^®^ III RT SuperMix for qPCR (Vazyme) was used for cDNA generation, RT-qPCR was performed using the ChamQ Universal SYBR qPCR Master Mix (Vazyme). GAPDH was used as the internal reference gene and the relative expression of indicated transcripts were calculated according to delta delta Ct method. All primers used in our study were listed at Supplementary Table 1.

### Cell Counting Kit-8 (CCK-8) assay

Cell Counting Kit-8 (CCK-8) (beyotime, China) were used to measure the PC cell viability. After synchronizing the cells at M phase to avoid interference by differences in cell cycle stage, we seeded the PC cells into 96- well plates. After 24 h cultivation, cells were stained with 100ul of CCK-8 reagent for 2 h at 37 °C. Absorbance readings for each well were measured at 450 nm using a microplate reader. The absorbance of cells in each group was detected after 24, 48, 72 and 96 h cultivation. All experiments were performed in triplicate and the IC50 values +were analyzed using GraphPad Prism software.

### Colony formation assay

For colony formation assays, PC cells of different groups were plated in six-well plates at a density of 500 cells per well and cultured for another 2 weeks. Then the cells were fixed with 4% paraformaldehyde for 15 min at room temperature and stained with 2% crystal violet for 20 min. After washing with water, the air-dried plates were visualized with the camera. Images were photographed and the number of colonies was Calculated.

### EdU staining

EdU assays were performed using the EdU Cell Proliferation Kit 555 (Beyotime, Shanghai, China) following the manufacturer’s protocols. Cells were labeled with 10um EdU at 37 °C for 2 h to stained with the EdU label, and the plates were then wash with PBS and fixed with 4% PFA then stained by Azide555 regent for 30 min. The stained cells were imaged with fluorescence microscopy, and the quantification of stained cells were assessed using ImageJ software. Each EdU experiment was repeated at least three times.

### Transwell assay

The 0.5 and 0.8 μm pore 24-well Transwell plates (BD Biosciences) were used for Transwell migration and invasion assays, respectively. For the migration assay, 5 × 10^5^ cells were suspended at the upper chamber of Transwell plate and supported with the none-FBS medium and the lower chamber medium supplemented with 10% FBS. For the invasive assay, 5 × 10^5^ cells were seeded in Matrigel-coated upper chamber and cultured in the FBS free medium while the lower chamber contained medium with 10% FBS. After 24 h of cultivation the migrated or invasive cells were stained with crystal violet and visualized under a microscope.

### Western-blotting assay

The total protein was extracted from cell lysate using the RIPA buffer with protease inhibitor cocktail, phosphatase inhibitors and PMSF (RIPA: cocktail: phosphatase inhibitors: PMSF = 100:1:1:1). For Co-IP assay the RIPA buffer was replaced by NP40 buffer. Then the lysate protein or immunoprecipitated protein were heated in SDS loading buffer for 5–10 min at 98 °C. The protein abundance of each group was analyzed by SDS-PAGE gel electrophoresis and western blotting. The GAPDH or Tublin protein detected by anti-GAPDH, anti-LaminB1 or anti-β-Actin antibodies was used as an internal reference to normalize gene expression in corresponding experiments. All antibodies used in this study were listed at Supplementary Table 1.

### Mass spectrum

Liquid chromatography tandem mass spectrometry (LC–MS/MS) was used for mass spectrometry analyses in our research. The PC cells were lysed using NP40 buffer and the extracted sampled were immunoprecipitated by anti-USP33 or anti-IgG antibody, the different group of immunoprecipitated proteins were then digested with modified sequencing grade trypsin, the fragmented peptides were analyzed by LC–MS/MS to identify USP33-interacting proteins.

### Luciferase reporter assay

To assess the regulatory effects of ZEB1 on USP33 mRNA, dual-luciferase reporter assay was performed as described previously [22]. The USP33 promoter region from 2000 bp upstream to 1 bp downstream of the transcription start site (TSS) was cloned into plasmid pGL3-Basic firefly luciferase reporter plasmid. Firefly or renilla luciferase activity were detected by the dual-luciferase reporter system (Promega, USA). Relative luciferase activity was calculated by normalizing firefly luciferase activity to that of Renilla luciferase.

### ChIP-qPCR

For ChIP assay, the cells were lysed using ChIP lysis buffer, crosslinked chromatin and protein complexes were then lysed by sonication. The obtained lysis were centrifuged at 10,000 × *g*, 4 °C for 10 min. Appropriate amount of samples were kept as input DNA sample. Immunoprecipitation were carried out using anti-ZEB1 or rabbit IgG. The immunoprecipitated samples were then washed using the wash buffer. The washed sample were centrifuged at 700 rpm for 1 min then eluted by ChIP elution buffer. Cross-linking was then reversed with decrosslinking buffer (0.2 M NaCl) overnight at 65 °C. The samples were then fixed with RNaseA at 37 °C for 1 h and treated with 0.5 M EDTA, 1 M Tris HCl and Proteinase K at 45 °C for 2 h. The samples were then dissolved in ddH_2_O for the following qPCR experiments. The qPCR was used to analyze binding of the ZEB1 to the USP33 promoter and the results were normalized by input.

### Co-IP

For coimmunoprecipitation, the PC cells transfected with corresponding plasmids were lysed using NP40 lysis buffer. The protein concentration was determined by BCA kit (Biosharp, Shanghai, China), cell lysate was then undergoing overnight incubation at 4 °C with corresponding IP antibody or isotype IgG followed by incubation with protein A/G magnetic beads (MCE, NJ, USA). The immunoprecipitate–bead complexes were washed thrice with IP washing buffer with rotation. The washed samples were pelleted using a magnetic rack and the supernatant was discarded. The immunoprecipitates were analyzed by Western blotting.

### Ubiquitination assays

For the ubiquitination assay, the 293T cells or PC cells were pretreated with the indicated plasmids for 24 h. After treated with 20 μM MG-132 for 6 h, the cells were lysed by NP40 buffer, the obtained lysates were then fixed with IP buffer. Next the TGFBR2 protein was immunoprecipitated with anti- Flag antibody and protein A/G magnetic beads, the TGFBR2 protein-beads complex was then purified using a magnetic rack and the supernatant was discarded. The immunoprecipitates were then subjected to Western blotting.

### Immunofluorescence staining

For immunofluorescence analysis, different groups of PC cells were seeded into multi-chamber slides and incubated at 37 °C. At 80% of confluency, cell slides were fixed in 4% paraformaldehyde for 20 min at room temperature, then 0.1% Triton X-100 were applied to permeabilized samples, next the samples were incubated with primary antibodies (USP33 or TGFBR2 antibody) at 4 °C overnight, followed by incubating with secondary antibodies. After the cell samples fixed and stained with DAPI, images were captured by immunofluorescence microscopy.

### Immunohistochemistry

The tissues of the PC bearing mice were separated and soaked in formaldehyde solution for further detection. For tissues form patients, tumor and normal samples were sectioned on slides with 4-μm thickness. The expression level of indicated proteins were determined using IHC on a tissue microarray. Samples were incubated with anti-USP33, anti-Ki-67, anti-PCNA, anti-TGFBR2 or anti-ZEB1 antibodies followed by incubation with an HRP-conjugated secondary antibody. Samples were then visualized with DAB and counterstained with hematoxylin, images were finally captured by a light microscope.

### Clinical samples collection

A total of 46 Tumor tissues and 39 of which with paired adjacent noncancerous tissues of PC patients were obtained from surgical specimens at the Department of Hepatobiliary Surgery, Renmin Hospital of Wuhan University. The collected patients tissues had all been pathologically diagnosed with PC. All data were collected with informed consent. All data was determined by histopathological examination. The clinical characteristics and patient information were analyzed from patient clinical records and pathology reports. All the clinical data were available in Supplementary Table 2.

### Subcutaneous xenograft experiments

The 4-week-old female BALB/c-nude mice, specific pathogen free (SPF), were purchased from GemPharmatech Co. Ltd, the 15 mice were randomly divided into three groups and bilateral subcutaneously inoculated with different group of PANC-1 cells (5 × 10^6^ cells/100 µl) to established the xenograft PC bearing model. Tumor sizes and volumes were monitored every three days. The tumor volume was calculated using the following formula: *V* (cm^3^) = 1/2 × length × width^2^. All animal experiments were approved by the Wuhan University Institutional Animal Care and Use Committee.

### liver metastasis model

The 4-week-old female BALB/c-nude mice, specific pathogen free (SPF), were purchased from GemPharmatech Co. Ltd, the mice were randomly divided into three groups (12 mice for each group). PC cells from each group were injected into the spleens of the nude mice (2 × 10^5^ cells/100 µl) to establish the liver metastasis model. 30 days after the injection, 6 mice of each group were randomly sacrificed by cervical dislocation and the number of metastatic nodules was calculated with a diameter bigger than 1.0 mm. The metastatic livers were collected for further histological analysis. The rest mice of each group was fed for the overall survival documentation, the death of some mice was recorded as the endpoint, while some of which were sacrificed by cervical dislocation as they kept survival till the end of our observation and the OS time was determined.

### Bioinformatics analysis

The Gene Expression Profiling Interactive Analysis (GEPIA) platform (http://gepia.cancer-pku.cn/), TCGA-PAAD dataset (https://portal.gdc.cancer.gov/) and GETx database (https://www.gtexportal.org/home%5B33) were used to analyze the expression and clinical correlation of USP33 in PC patients. The GO analysis and KEGG analysis were conducted using the R software (version 4.0.1). The protein sequence analysis and the construction predication of USP33 were carried out using the InterPro (https://www.ebi.ac.uk/interpro/), Uniprot (https://www.uniprot.org/) and Alphafold (https://alphafold.ebi.ac.uk/) database.

### Statistical analysis

SPSS 22.0 software (Chicago, IL, USA) and GraphPad Prism 9.0 (La Jolla, CA, USA) were used to analyze the data in our research. We used *t*-test or one-way ANOVA to analyze the significant differences between different groups. Data were expressed as mean ± SD from three individual experiments and a *p* value < 0.05 was considered statistically significant.

## Results

### USP33 was highly expressed in PC tissues and cells

**Fig. 1:**
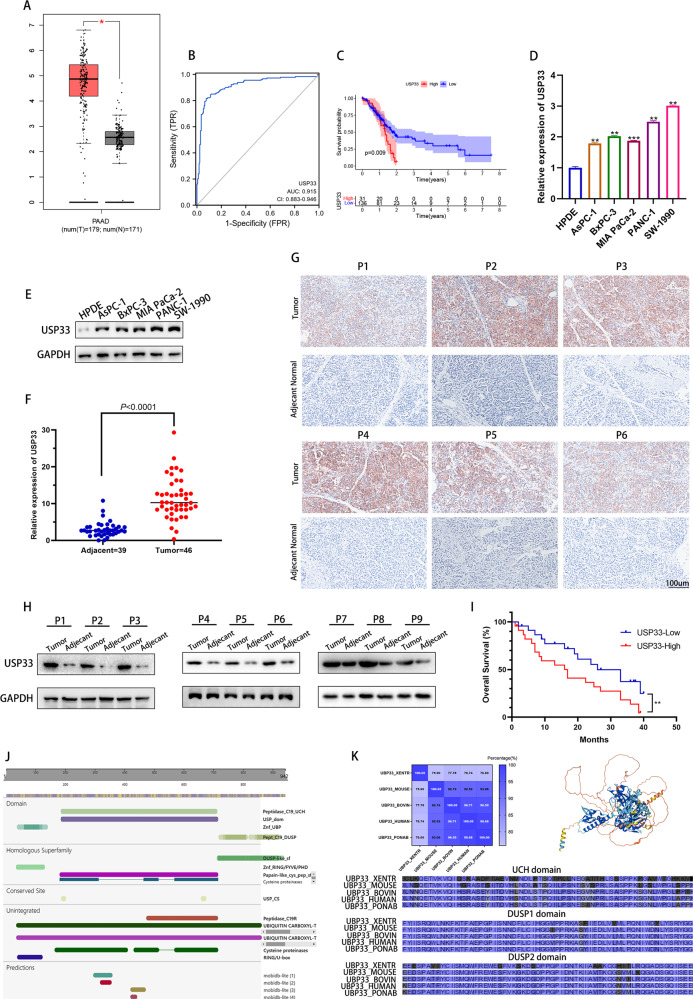
USP33 was highly expressed in PC tissues and cells. **A** The expression of USP33 analyzed in GEPIA database using TCGA-PAAD and GTEx datasets. **B** The ROC curve of USP33 in TCGA-PAAD dataset. **C** The USP33 related survival analysis in TCGA-PAAD dataset. **D** The expression of USP33 mRNA among PC cell lines and normal pancreatic cell line. **E** The expression of USP33 protein among PC cell lines and normal pancreatic cell line. **F** The expression of USP33 in our collected PC patients. **G** The representative IHC results of USP33 among PC patients. **H** The western-blotting result revealed the expression of USP33 protein in PC patients. **I** The USP33 related survival analysis on our collected clinical data. **J** The graphic illustrate of USP33 domain predicted by online database. **K** The predicted protein structure and conserved functional domains of USP33.

Our analysis of TCGA-PAAD dataset showed that USP33 was upregulated in PC samples compared with normal ones (Fig. 1A). Analysis of TCGA clinical data showed that PC patients with higher USP33 expression were accompanied with poorer overall survival, meanwhile, the ROC analysis suggested that USP33 was a significant predictor of tumor versus normal subtypes (AUC = 0.915) (Fig. 1B, C).The qRT-PCR and western-blotting experiments also supported our bioinformatic analysis, the mRNA and protein level of USP33 was higher in PC cells or tumor samples than that of normal pancreatic ductal cells or adjacent normal samples (Fig. 1D–F). The immunohistochemical analysis and western-blotting demonstrated that patient-derived pancreatic samples exhibited a higher expression of USP33 protein but adjacent normal tissues showed a negligible USP33 expression (Fig. 1G, H). Moreover, our collected clinical data showed that the expression of USP33 in PC patients was negatively correlated with patient overall survival (OS) (Fig. 1I). Protein sequence analysis revealed that USP33 contained three highly conserved functional domains among species, the UCH domain, the DUSP1 domain, and the DUSP2 domain (Fig. 1J, K). Collectively, our results indicated that USP33 was upregulated in PC samples, the expression of USP33 was negatively correlated with the prognosis of PC patients, and there were three highly conserved functional domains that may define the vital function of USP33.

### USP33 promoted the proliferation, migration and invasion of PC cells

**Fig. 2:**
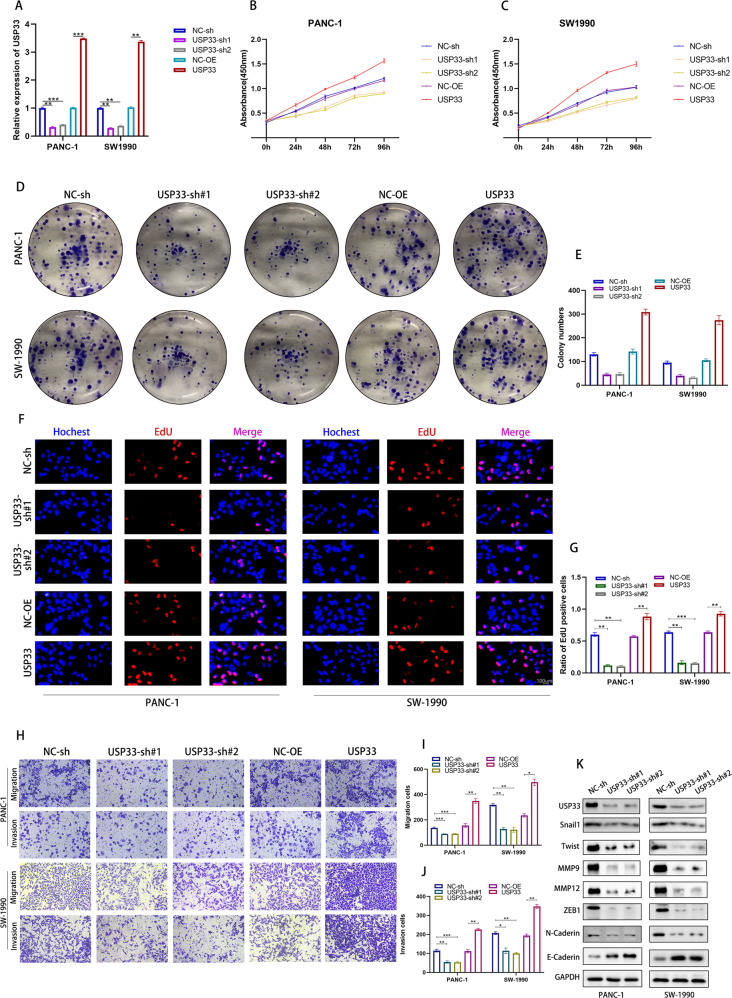
USP33 promoted the proliferation, migration and invasion of PC cells. **A** qPCR validation of USP33 shRNA and overexpression plasmids transfection efficiency **B**, **C** The quantification of CCK-8 assay of PC cells transfected with indicated plasmids. **D** The colony formation assay of PC cells transfected with indicated plasmids. **E** The quantification of colony formation assay. **F** The EdU assay of PC cells transfected with indicated plasmids. **G** The quantification of EdU assay. **H** The Transwell migration and invasion assay of PC cells transfected with indicated plasmids. **I**, **J** The quantification of Transwell migration and invasion assay. **K** The western-blotting assay revealed the alteration of PC malignant biomarkers’ protein expression in PC cells transfected with indicated plasmids.

To define the function of USP33 in the proliferation, migration and invasion of PC cells, we transfected PC cells with USP33 shRNA or overexpression plasmids and observed their effect on the malignant phenotype of PC cells (Fig. 2A). CCK-8 assay demonstrated that knockdown of USP33 significantly dampened the viability of PC cells, while opposite results were observed in PC cells transfected with USP33 overexpression plasmids (Fig. 2B, C). The colony formation experiment showed similar results with our CCK-8 assay, the proliferation ability of PC cells decreased rapidly after the transfection of USP33 shRNA while overexpression of USP33 had the opposite effect (Fig. 2D, E). Moreover, we labeled EdU-positive PC cells to monitor the proliferation rate of PC cells, our data showed that EdU-positive cells was markedly reduced after USP33 downregulation while USP33 overexpression increased the proliferation rate of PC cells (Fig. 2F, G). To assess whether USP33 affected the invasiveness and migration of PC cells, we performed the transwell migration and invasion assays and found that USP33 knockdown significantly inhibited the migration and invasiveness of PC cells while USP33 overexpression showed the opposite effect (Fig. 2H–J). Further, we performed western-blotting assay to evaluate the effect of USP33 on biomarkers of proliferation, migration and invasion, the results showed that the expression of Snail1, Twist, MMP9, MMP12, ZEB1,N-Caderin decreased rapidly while the protein level of E-Caderin increased after the knockdown of USP33 (Fig. 2K).

### USP33 was involved in the TGF-β signaling pathway

**Fig. 3:**
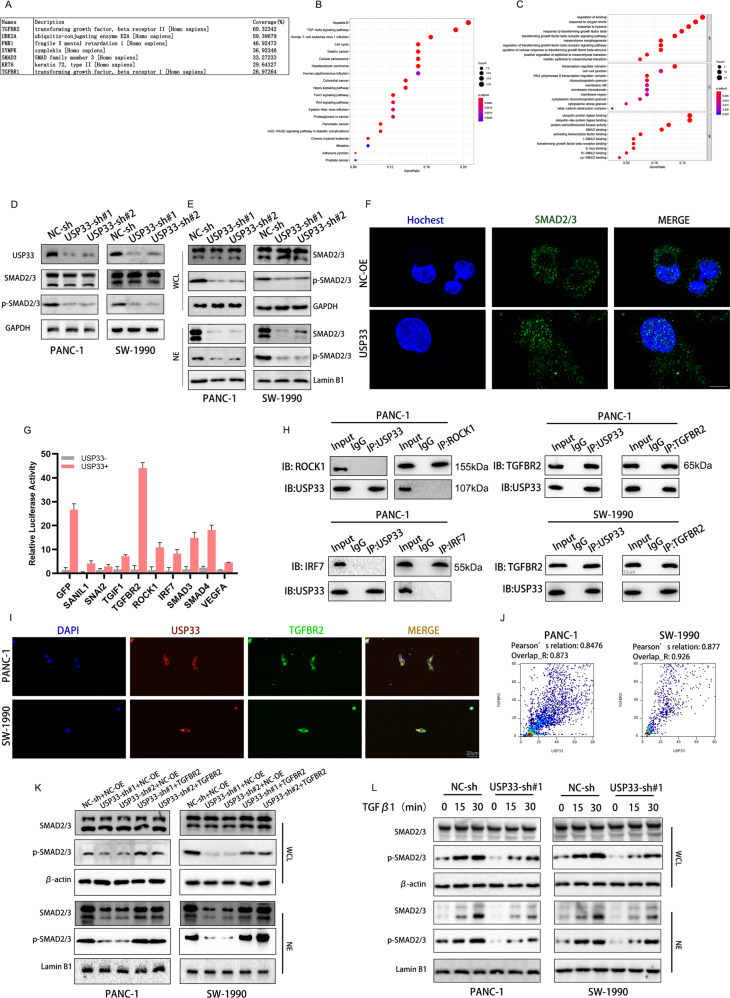
USP33 was involved in the TGF-β signaling pathway. **A** The LC–MS/MS results of USP33 binging proteins. **B** The KEGG analysis of the LC–MS/MS result. **C** The GO analysis of the LC–MS/MS result. **D** The alteration of SMAD2/3 expression in PC cells transfected with USP33 shRNAs. **E** The expression of SMAD2/3 in the whole cell lysate (WCL) and nuclear extraction (NE) in PC cells transfected with USP33 shRNAs. **F** The nuclear translocation of SMAD2/3 in PC cells visualized by confocal laser scanning microscope. **G** The luciferase complementary results revealed the binding efficiency between USP33 and indicated target proteins. **H** The Co-IP experiment verifying the interaction between USP33 and the indicated targets. **I** The colocalization between USP33 and TGFBR2 visualized by fluorescence microscope. **J** The quantification of the immunofluorescence result. **K** The alteration of SMAD2/3, p-SMAD2/3 expression in the nuclear of PC cells transfected with the indicated plasmids. **L** The alteration of SMAD2/3, p-SMAD2/3 expression in the nuclear of PC cells transfected with the indicated plasmids and treated with TGFβ1 for indicated time.

Considering USP33 as a member of deubiquitinating enzyme, we tend to find the potential target of USP33 through mass spectrum analysis (Fig. 3A). We then conducted the GO and KEGG analysis of screened proteins, the results indicated that USP33 may participate in the regulation of TGF-β signaling pathway (Fig. 3B, C). To verify our speculation, we tested the expression of SMAD2/3 and p-SMAD2/3 in PC cells after the knockdown of USP33, our experiment demonstrated that USP33 elevated the expression of p-SMAD2/3 with no alteration in protein level of SMAD2/3. Further, we separated the nuclear proteins in PC cells transfected with USP33 shRNA, the western-blotting assay showed that after the knockdown of USP33, the expression of SMAD2/3 and p-SMAD2/3 in the nuclear decreased rapidly (Fig. 3D, E). The confocal microscopy result showed that USP33 promoted the translocation of Smad2/3 from the cytoplasm to the nucleus (Fig. 3F). The results above implied that USP33 regulated the nuclear translocation of SMAD2/3 and their phosphorylation. Noticing that several regulators of TGF-β signaling were detected in our mass spectrum analysis, we performed a luciferase complementation assay to screened the exact target of USP33, the result showed that TGFBR2 exhibited the strongest binding with USP33 (Fig. 3G). Further Co-IP experiments showed a similar result with our luciferase complementation assay, TGFBR2 and USP33 could bind to each other while the other candidates had no interaction with USP33 (Fig. 3H). Similar results were obtained by immunofluorescence microscopy, the result showed that USP33 and TGFBR2 colocalized with each other in PC cells (Fig. 3I, J). Considering that TGFBR1 may be involved in the USP33-mediated SMAD signaling, we performed the western-blotting assay and found that USP33 knockdown decreased the expression of TGFb targeted molecules like PAI-1 and VEGF but didn’t alternate the expression of TGFBR1 (Fig. S1A, B). Moreover, our results showed that USP33 knockdown led to a decrease in the phosphorylation and translocation of SMAD2/3, while the TGFBR2 overexpression rescued the effect of USP33 knockdown (Fig. 3K). We further investigated the correlation between TGFBR2 and USP33 in the regulation of TGF-β signaling pathway. Under the stimulation of TGFβ1, PC cells transfected with USP33 shRNA exhibited the lower level of phosphorylated or nuclear located SMAD2/3 (Fig. 3L). In collusion, our experiment demonstrated that USP33 served as a regulator of TGF-β signaling pathway through its interaction with TGFBR2.

### USP33 regulated the malignant phenotype of PC through TGFBR2

**Fig. 4:**
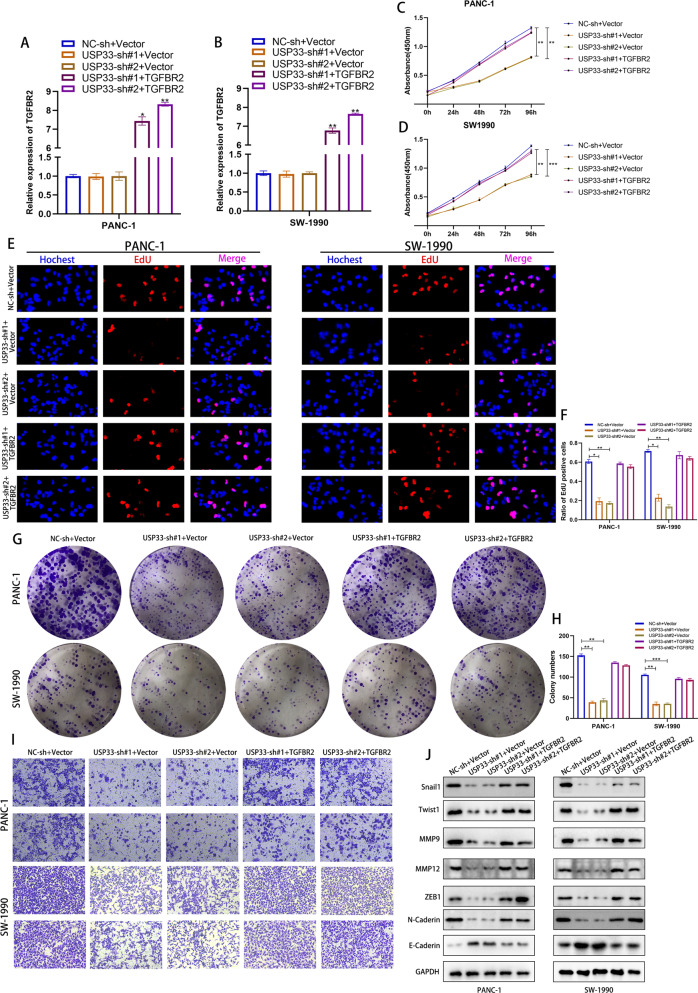
USP33 regulated the malignant phenotype of PC through TGFBR2. **A**, **B** qPCR validation of indicated plasmids transfection efficiency **C**, **D** The quantification of CCK-8 assay of PC cells transfected with indicated plasmids. **E** The EdU assay of PC cells transfected with indicated plasmids. **F** The quantification of EdU assay. **G**, **H** The colony formation assay and quantification of PC cells transfected with indicated plasmids. **I** The Transwell migration and invasion assay of PC cells transfected with indicated plasmids. **J** The western-blotting assay revealed the alteration of PC malignant biomarkers’ protein expression in PC cells transfected with indicated plasmids.

To investigate the role of TGFBR2 in USP33-mediated PC cell malignance, we performed the corresponding rescue experiments. Our CCK-8 assay showed that USP33 knockdown significantly impaired the viability of PC cells while the overexpression of TGFBR2 rescued its effect (Fig. 4A–D). Similar results were observed in EdU assays and colony formation experiments, the proliferation ability of PC cells decreased rapidly after transfection of USP33 shRNA while overexpression of TGFBR2 remedied this phenotype (Fig. 4E–H). Moreover, the transwell assay showed that TGFBR2 overexpression rescued the effect of USP33 on the migration and invasion of PC cells (Fig. 4I). Further western-blotting experiment demonstrated that USP33 knockdown inhibited the expression of biomarkers of migration and invasion while TGFBR2 overexpression rescued its effect (Fig. 4J).

### USP33 promoted the recycling of TGFBR2

**Fig. 5:**
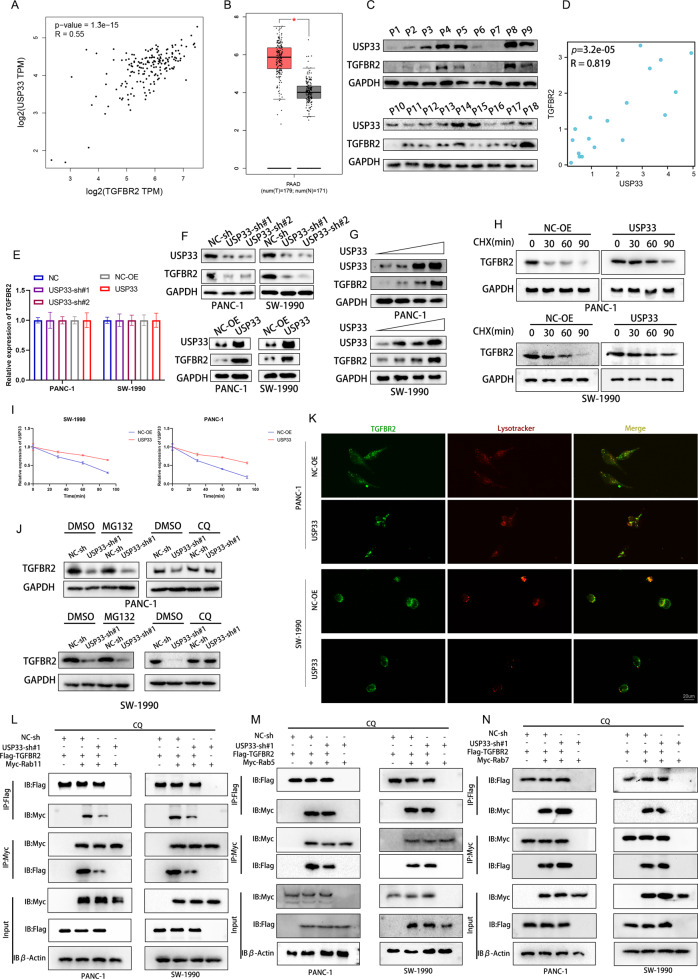
USP33 promoted the recycling of TGFBR2. **A** The correlation between the expression of USP33 and TGFBR2 mRAN in TCGA-PAAD. **B** The expression of TGFBR2 in GEPIA database. **C** The expression of TGFBR2 and USP33 in PC patients derived tissues. **D** The correlation between the expression of USP33 and TGFBR2 protein in PC patients derived tissues. **E** The expression of TGFBR2 mRNA in PC cells transfected with USP33 shRNAs or overexpression plasmids. **F** The expression of TGFBR2 protein in PC cells transfected with USP33 shRNAs or overexpression plasmids. **G** The expression of TGFBR2 protein in PC cells transfected with different concentration of USP33 overexpression plasmids. **H** The expression of TGFBR2 protein in PC cells transfected with indicated plasmids and treated with CHX for indicated time. **I** The quantification of TGFBR2 expression in CHX treated PC cells. **J** The expression of TGFBR2 in PC cells treated with DMSO, CQ or MG-132 and transfected with USP33 shRNAs. **K** The colocalization between USP33 and lysotracker visualized by fluorescence microscope. **L** The Co-IP result of TGFBR2 and Rab11 in PC cells transfected with USP33 shRNA. **M** The Co-IP result of TGFBR2 and Rab5 in PC cells transfected with USP33 shRNA. **N** The Co-IP result of TGFBR2 and Rab7 in PC cells transfected with USP33 shRNA.

Our previous experiments proved that USP33 interacted with TGFBR2, while the effect of USP33 in TGFBR2 was unclear. By analyzing data from TCGA-PAAD dataset, we found that TGFBR2 was high expressed in PC patients and the USP33 mRNA expression was positively correlated with TGFBR2 (Fig. 5A, B). Moreover, our western-blotting experiment showed the protein level of USP33 in PC patients was also positively correlated with TGFBR2 (Fig. 5C, D). Further we diminished the expression of USP33 in PC cells and detected the alteration of TGFBR2 expression at mRNA and protein levels. Our results suggested that USP33 knockdown inhibited the expression of TGFBR2 protein, the overexpression of USP33 had the opposite effect on TGFBR2 protein while no alteration was observed in TGFBR2 mRNA after the manipulation on USP33 expression (Fig. 5E–G), all these results indicated that USP33 may participate in the posttranslational modification of TGFBR2. To verify our hypothesis, we measured the half-life of TGFBR2 protein in PC cells treated with CHX, our results showed that overexpression of USP33 inhibited the degradation of TGFBR2 (Fig. 5H, I). While there were two main way for protein degradation, we treated the PC cells with the lysosome inhibitor chloroquine (CQ) or the proteasome inhibitor MG-132 to screen the exact degradation way of TGFBR2 regulated by USP33. Our results showed that CQ rather than the MG-132 rescued the inhibition effect of USP33 shRNA on TGFBR2 (Fig. 5J). These results indicated that USP33 was involved in the degradation of TGFBR2 by lysosome. The IF assay showed that after the overexpression of USP33, the colocalization of TGFBR2 with lysosome was impaired, meanwhile USP33 overexpression promoted the accumulation of TGFBR2 in cell membrane (Fig. 5K). Previous studies suggested that various kinds of receptors in cell membrane was regulated by endocytosis and lysosome [23, 24]. We hypothesized that USP33 may be involved in the endocytosis of TGFBR2. Our Co-IP analysis demonstrated that after knockdown of USP33, the interaction between TGFBR2 and the recycling endosome marker Rab11 was impaired while no effect was observed on Rab5 (early endosome marker) and Rab7 (late endosome biomarker) (Fig. 5L–N). Moreover, our data showed that USP33 promoted the recycling of TGFBR2 to cell membrane in PC cells during its internalization (Fig. S2). In conclusion, our study suggested that USP33 prevented the degradation of TGFBR2 by lysosome by promote its recycling by the recycling endosome.

### USP33 deubiquitinated TGFBR2 and removed the K63 lysate conjuncated ubiquitin chain

**Fig. 6:**
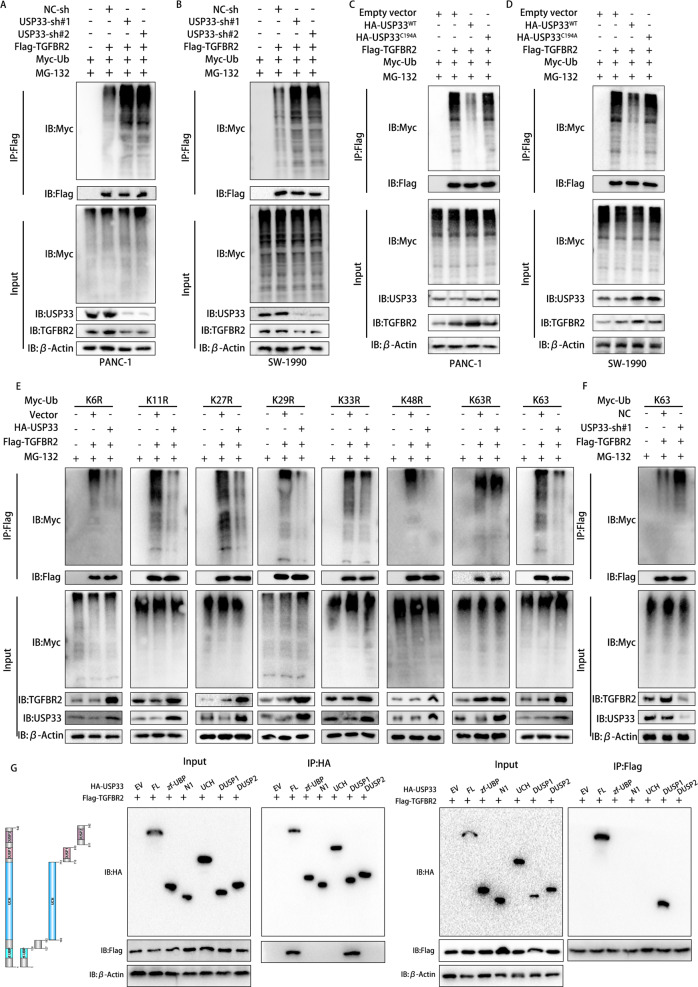
USP33 deubiquitinated TGFBR2 and removed the K63 lysate conjuncated ubiquitine. **A**, **B** The ubiquitination level of TGFBR2 in PC cells transfected with USP33 shRNAs. **C**, **D** The ubiquitination level of TGFBR2 in PC cells transfected with USP33 overexpression plasmids or USP33C194A mutant plasmids. **E** The ubiquitination level of TGFBR2 in 293T cells transfected with indicated Ub mutants and USP33 overexpression plasmids. **F** The ubiquitination level of TGFBR2 in 293T cells transfected with USP33 shRNA and indicated Ub K63 only mutant. **G** The reciprocal Co-IP results of USP33 truncation and TGFBR2 in 293T cells.

Our previous results showed that USP33 protected TGFBR2 degradation from lysosome, therefore we designed the corresponding experiments to verify if USP33 regulated the posttranslational modification of TGFBR2. Our ubiquitination assay showed that after the knockdown of USP33, the ubiquitination level of TGFBR2 increased rapidly (Fig. 6A, B). Moreover, after the transfection of USP33 mutant (C194A), we observed no alteration on the ubiquitination level of TGFBR2 compared with the empty vector group(Fig. 6C, D). Further, we transfected the 293T cells with different types of ubiquitin mutants and Flag-tagged TGFBR2, then we detected the ubiquitination level of TGFBR2 by western blot. Our results demonstrated that the K63R-Ub mutant and USP33 co-transfection had no effect on TGFBR2 ubiquitination, while the co-transfection of USP33 with other Ub mutants impaired the ubiquitination of TGFBR2 (Fig. 6E). Further, our western-blotting result showed that USP33 knockdown increased the ubiquitination level of TGFBR2 in 293T cells transfected with K63 only Ub plasmid (Fig. 6F). Moreover, we transfected 293T cells with the USP33 truncation constructs and immunoprecipitating TGFBR2 or USP33 respectively to confirm the exact binding region for USP33 and TGFBR2. Our results showed that only the truncation containing the DUSP1 domain of USP33 successfully coimmunoprecipitated the TGFBR2 protein (Fig. 6G). All in all, our data revealed that USP33 regulated the K63 deubiquitination of TGFBR2 and promoted the its protein stability, meanwhile, the binding between USP33 and TGFBR2 was dependent on the DUSP1 domain of USP33.

### USP33 was transcriptional activated by ZEB1

**Fig. 7:**
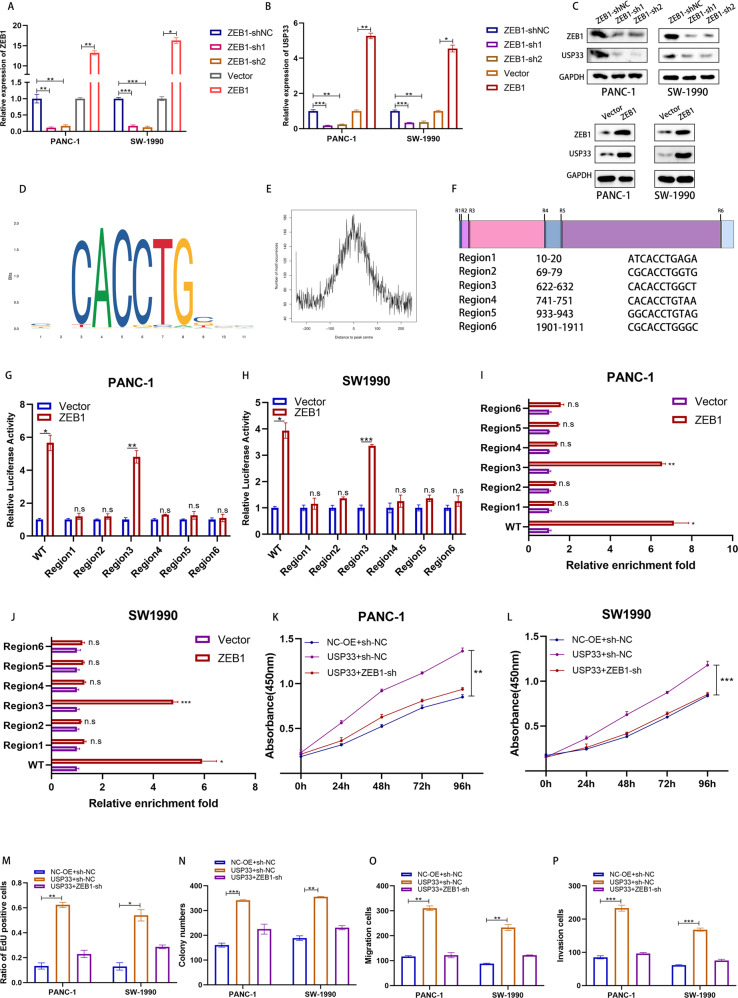
USP33 was transcriptional activated by ZEB1. **A** The expression of ZEB1 in PC cells transfected with ZEB1 shRNA or overexpression plasmids. **B** The expression of USP33 mRNA in PC cells transfected with ZEB1 shRNA or overexpression plasmids. **C** The expression of USP33 protein in PC cells transfected with ZEB1 shRNA or overexpression plasmids. **D**, **E** The motif of ZEB1 predicted by JASPAR database. **F** The possible regions in USP33 promoter for the binding of ZEB1. **G**, **H** The dual-luciferase reporter assay revealed the binding between indicated regions of USP33 promoter and ZEB1. **I**, **J** The ChIP-qPCR assay revealed the binding between indicated regions of USP33 promoter and ZEB1. **K**, **L** The CCK-8 assay in PC cells co-transfected with USP33 overexpression plasmids and ZEB1 shRNA. **M** The quantification of EdU assay in PC cells co-transfected with USP33 overexpression plasmids and ZEB1 shRNA. **N** The quantification of colony formation assay in PC cells co-transfected with USP33 overexpression plasmids and ZEB1 shRNA. **O**, **P** The quantification of Transwell assay in PC cells co-transfected with USP33 overexpression plasmids and ZEB1 shRNA.

Considering our previous results, USP33 had no effect on the mRNA expression of TGFBR2, while the TCGA-PAAD dataset revealed that the expression of USP33 mRNA positively correlated with TGFBR2. Moreover, our western-blotting and PCR assay showed that the stimulation of TGF-β evaluated the expression of USP33 in PC cells (Fig. S3A, B). Taking together, we hypothesized that USP33 mRNA may be regulated by TGFBR2. Therefore, we analyzed the JASPAR database and screened the TGF-β related genes, ZEB1 was considered as the potential candidate regulator of USP33. Next, we manipulated the ZEB1 expression in PC cells and found that ZEB1 overexpression elevated the mRNA and protein level of USP33 (Fig. 7A–C, Fig. S4). By analyzing the JASPAR database, we identified the possible ZEB1 binding regions of TGFBR2 promoter and got 6 putative sites (Fig. 7D–F). Dual-luciferase reporter assays and the ChIP-qPCR experiments demonstrated that ZEB1 could bind to the 622-632 region of USP33 at the chromatin level (Fig. 7G–J). Further, we performed the corresponding rescue experiments and found that ZEB1 knockdown could rescue the effect of USP33 on the proliferation, migration and invasion of PC cells (Fig. 7K–P, Fig. S5).

### USP33/TGFBR2 axis promoted the tumor growth and metastasis of PC in vivo

**Fig. 8:**
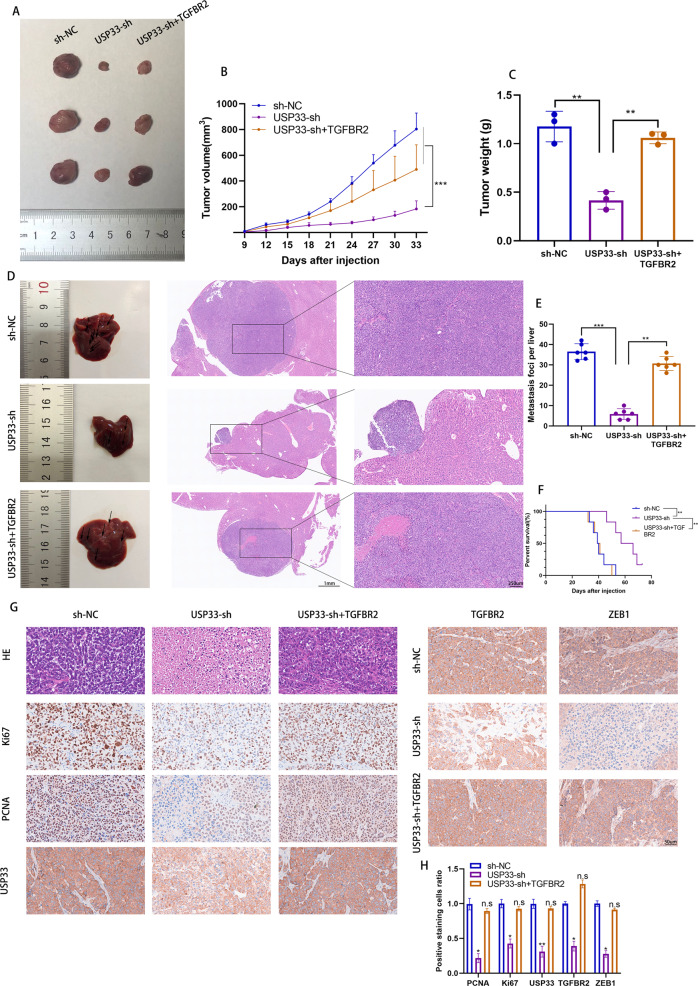
USP33/TGFBR2 axis promoted the tumor growth and metastasis of PC in vivo. **A** The xenografted PC tumor derived from nude mice injected with indicated PC cells. **B** The volume of xenografted PC tumor in nude mice at indicated time after injection. **C** The weight of xenografted PC tumor derived from nude mice injected with indicated PC cells. **D** The metastatic liver tissues derived from nude mice injected with different group of PC cells. **E** The quantification of metastatic foci in metastatic liver tissues. **F** The survival of liver metastasis nude mice model. **G** The representative HE and IHC results of indicated proteins in xenografted PC tumor. **H** The quantification of IHC results.

Given the important role of USP33 in regulating the proliferation and invasion of PC cells in vitro, we next verified the function of USP33 on the tumor growth and metastasis of PC in vivo. Our results showed that USP33 deficiency significantly decreased the tumor growth of subcutaneous xenograft nude mice while the overexpression of TGFBR2 rescued the effect of USP33 (Fig. 8A–C). The IHC results of xenografted tumor showed that USP33 depletion inhibited the expression of Ki67, PCNA, TGFBR2 and ZEB1 (Fig. 8G, H). Further, we established the liver metastasis model and evaluated the effect of USP33 on PC liver metastasis in vivo. The results showed that knockdown of USP33 inhibited the liver metastasis of PC and increased the OS of PC bearing nude mice, while the overexpression of TGFBR2 remedied this phenotype (Fig. 8D–F). Taking together, our results demonstrated that USP33 promoted the tumor growth and metastasis of PC in a TGFBR2-dependent manner.

## Discussion

PC was considered as one of the most intractable malignancies, the mortality rate of PC approaches its incidence rate [25]. The biggest obstacle for PC diagnosis and treatment were the highly invasive property and the lack of specific diagnostic markers [26]. Therefore, it’s urgent for researchers to find an effective therapeutic target of PC. PC had the highest incidence of TGF-β pathway mutations among cancers and the TGFβ signaling acted as a double-edged sword’ in the progression of PC. The TGF-β signaling consists of noncanonical and canonical TGF-β signaling pathways and the canonical pathway which was SMAD-dependent had been well characterized [27, 28]. The activation of TGFBR2 initiated the TGF-β-SMAD signaling, then it recruited and phosphorylated TGFBR1 to maintain the signaling transduction. Phosphorylated TGFBR1 promoted the nuclear translocation of SMAD2/3 which eventually regulated the transcription of downstream genes [29]. There were various cancer-related genes involved in TGFβ signaling such as ZEB1, Snail1 and Vimentin that played vital roles in the malignant progression of PC [30]. Although numerous studies focused on the biological function of TGFβ signaling in PC development, the exact mechanism remained to be elucidated. In our study, we found that USP33 could serve as an important regulator of TGFβ signaling and may provide a new explanation of TGFβ activation. These indicated that USP33 could be applied as a potential therapeutic target of PC.

As a ubiquitous posttranslational modification, ubiquitination participated in various kinds of biological progresses including protein sorting, degradation, DNA repairment and transcriptional activation. The balance between ubiquitination and deubiquitylation determined the fate of tumor cells. Ubiquitination depends on the sequential action mediated by three key ubiquitination-related enzymes: ubiquitin-activating enzyme (E1), ubiquitin-conjugating enzyme (E2), and ubiquitin-ligasing enzyme (E3). The E1 recruited the ubiquitin molecular to E2 and the E3 transferred ubiquitin from E2 to its recognized targeted protein [31]. There were seven main types of ubiquitin ligase chains including K6, K11, K27, K29, K33, K48 and K63, different forms of protein ubiquitination led to different fates of proteins. The K48 and K11 polyubiquitination chains were involved in the proteasomal degradation signal. The K6 and K33 ubiquitination was reported to be regulators of DNA damage response [32–34]. The K63 polyubiquitination has been reported to mediate the signal transduction, protein translocation and autophagy while the function of K27 and K29 poly-Ubiquitinated proteins remained unclear [35, 36]. As a reverse procedure of ubiquitination, deubiquitination was decided by various deubiquitinases, similar to the ubiquitination process, different type of deubiquitination regulated different type of biological function. Previous studies had reported aberrant activity of ubiquitination or deubiquitination systems involved in the development of tumors. Qin et al. reported that BAP1 interacts directly with KLF5 and stabilizes KLF5 via deubiquitination and the KLF5/BAP1/HCF-1 complex promoted the tumorigenesis and lung metastasis of breast cancer by inhibiting p27 gene expression [37]. Similarly, USP3 was reported to promote the gastric cancer progression and metastasis by deubiquitinating COL9A3 and COL6A5 [38]. In our research, we reported for the first time that USP33 mediated the proliferation and metastasis of pancreatic cancer through its deubiquitinating function which may provide a novel explanation for PC malignant development.

USP33 was a member of the ubiquitin-specific protease (USP) family which was the largest deubiquitination enzymes (DUBs) family. There had been numerous studies reported the vital roles of USP33 in the progression of diverse tumors. Wen et al. reported that USP33 functioned as a tumor suppressor of lung cancer by promoting the protein stability of Robo1 and inhibiting cell migration of lung cancer cells [39]. In hepatocellular carcinoma USP33 functioned as an oncogene, the expression of USP33 was upregulated in HCC patients and the USP33 expression was negatively correlated with the prognosis of HCC patients. Mechanistically, USP33 directly bound SP1 protein and decrease its ubiquitination and enhance its protein stability, thereby upregulating c-Met expression which eventually promoted the migration and invasion of HCC [40]. Moreover, USP33 was reported to be overexpressed in prostate cancer cells and tissues and functioned as an oncogene of prostate cancer. USP33 could inhibit docetaxel-induced apoptosis of prostate cancer cells, including androgen-independent prostate cancer cells, mechanistically, USP33 could inhibit the Lys48 (K48)-linked polyubiquitination of DUSP1 which led to impaired JNK activation and apoptosis in prostate cancer [19]. Though it had been reported that USP33 participated in regulation of malignant phenotype of different tumors, there were no research demonstrating the role of USP33 in pancreatic cancer, in current study we found for the first time that USP33 may serve as an oncogene in PC. Our data showed that USP33 was abnormally expressed in PC cells and tissues and the expression of USP33 was negatively correlated with the prognosis of PC patients. Through our function experiments, we found that USP33 promoted the proliferation, migration and invasion of PC in vitro and in vivo. Our further experiments revealed that USP33 mediated the progression of PC cells through TGFBR2 which was a key regulator of TGFβ signaling pathway. Mechanistically, USP33 interacted with TGFBR2 through DUSP1 domain of USP33, moreover, USP33 enhanced the protein stability of TGFBR2 by removing the K63-linked ubiquitin chains on TGFBR2. The USP33-regulated deubiquitination of TGFBR2 avoided its lysosomal degradation, meanwhile USP33 promoted the colocalization of TGFBR2 with recycling endosome which led to its accumulation on the membrane and the activation of TGFβ signaling. Moreover, the downstream gene of TGFβ named ZEB1 was intriguing the transcriptional regulator of USP33, we found that ZEB1 promoted the transcription of USP33 through binding to the 622-632 site of USP33 promoter. Taking together, our study found the positive loop between USP33 and TGF-β pathway which eventually accelerated the progression of PC, it may inspire researchers to develop therapeutics targeting the USP33-TGFβ positive loop and provide novel treatment options for PC.

## Supplementary information

aj-checklist

supplementary FIGS1

supplementary FIGS2

supplementary FIGS3

supplementary FIGS4

supplementary FIGS5

supplementary table1

supplementary table2

supplementary figure legend

Original Data File

## Data Availability

All data generated and analyzed during this study are included in this published article and are available on request.
