# Supplementary figures and images for: USP33 promotes pancreatic cancer malignant phenotype through the regulation of TGFBR2/TGFβ signaling pathway
Source: Cell Death Dis. 2023 Jun 15;14(6):362. doi: 10.1038/s41419-023-05871-4 (PMC10272277; doi:10.1038/s41419-023-05871-4)

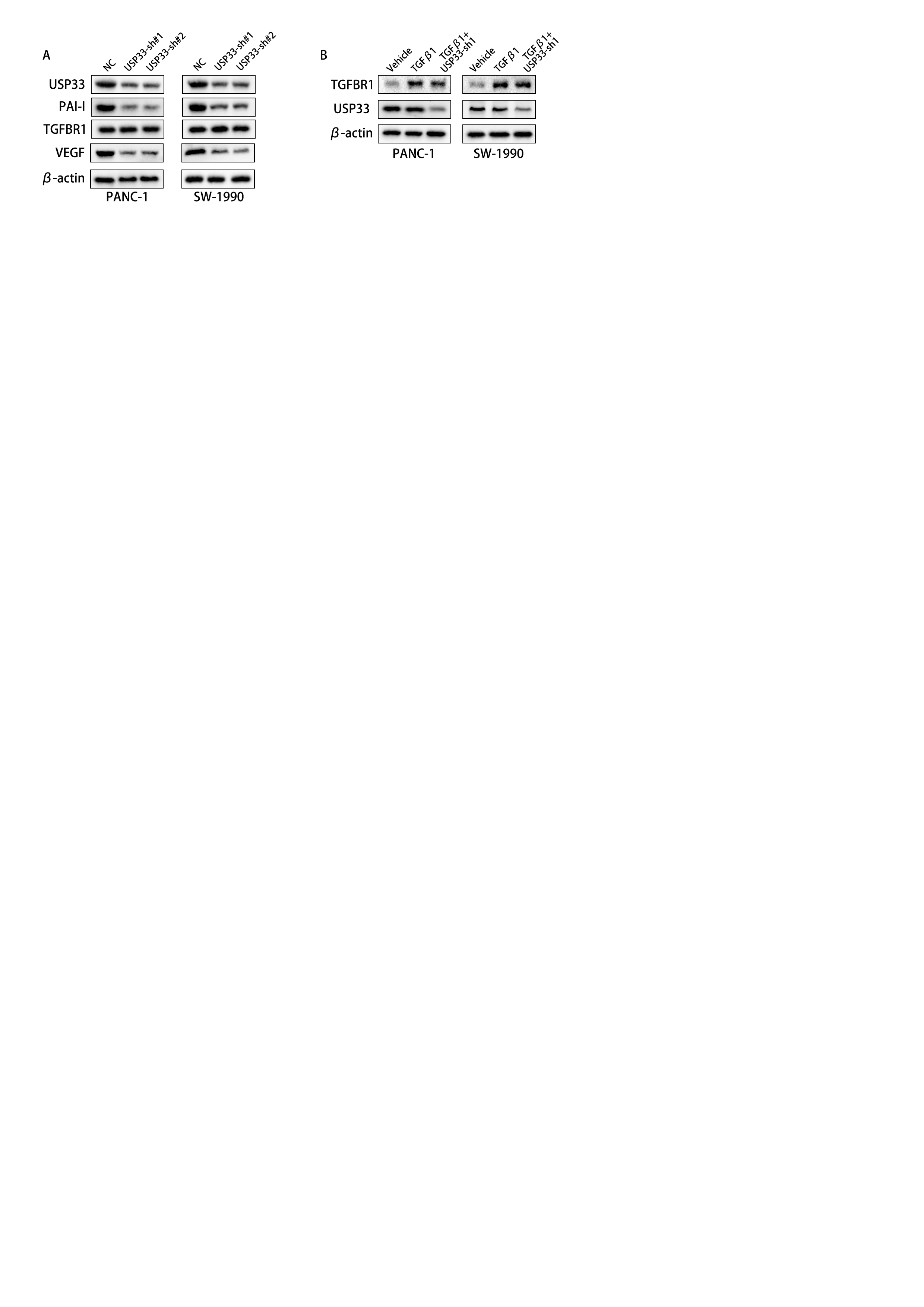

Supplement: Supplementary file 2 — supplementary FIGS1 [file 41419_2023_5871_MOESM2_ESM.tif]

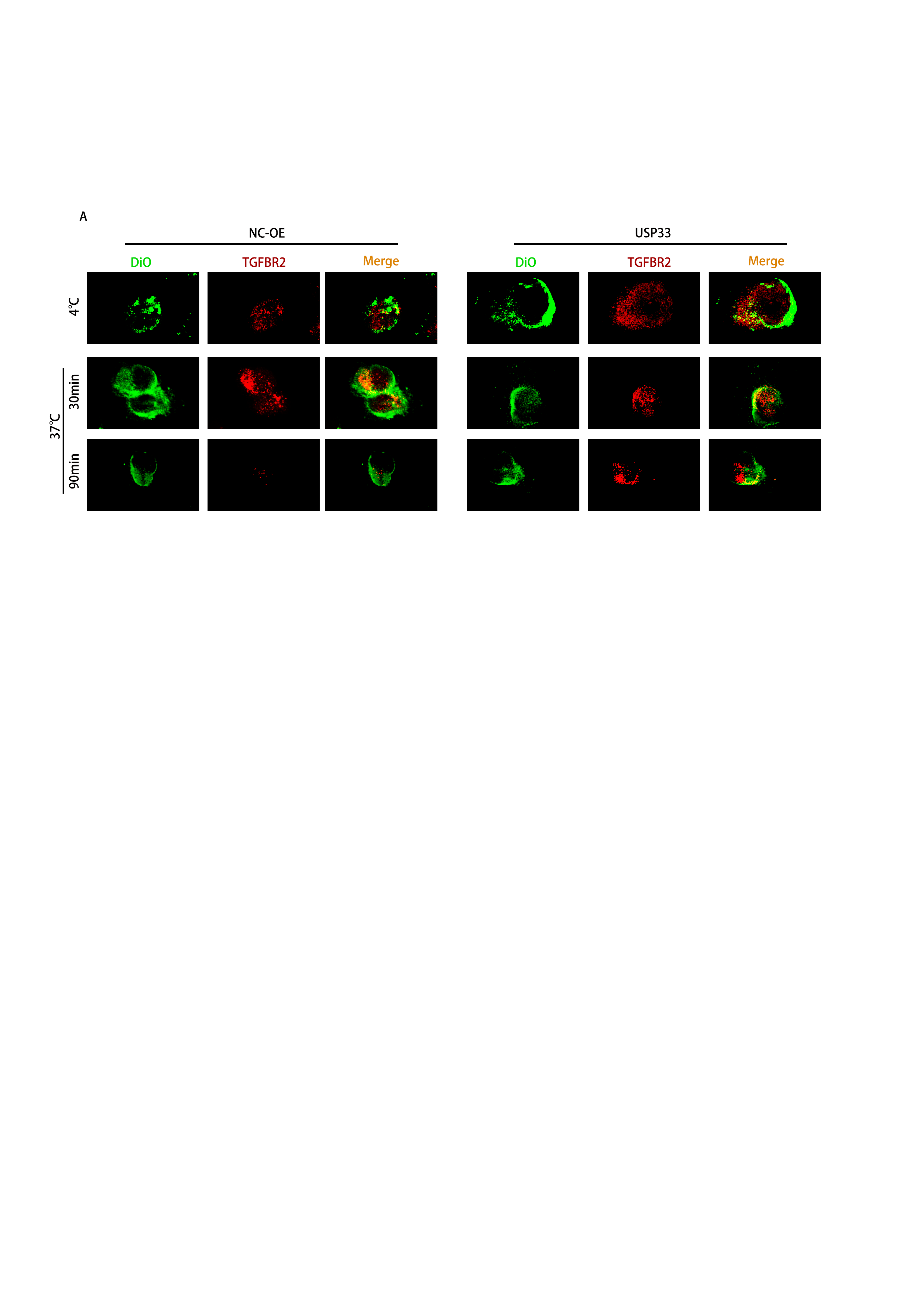

Supplement: Supplementary file 3 — supplementary FIGS2 [file 41419_2023_5871_MOESM3_ESM.tif]

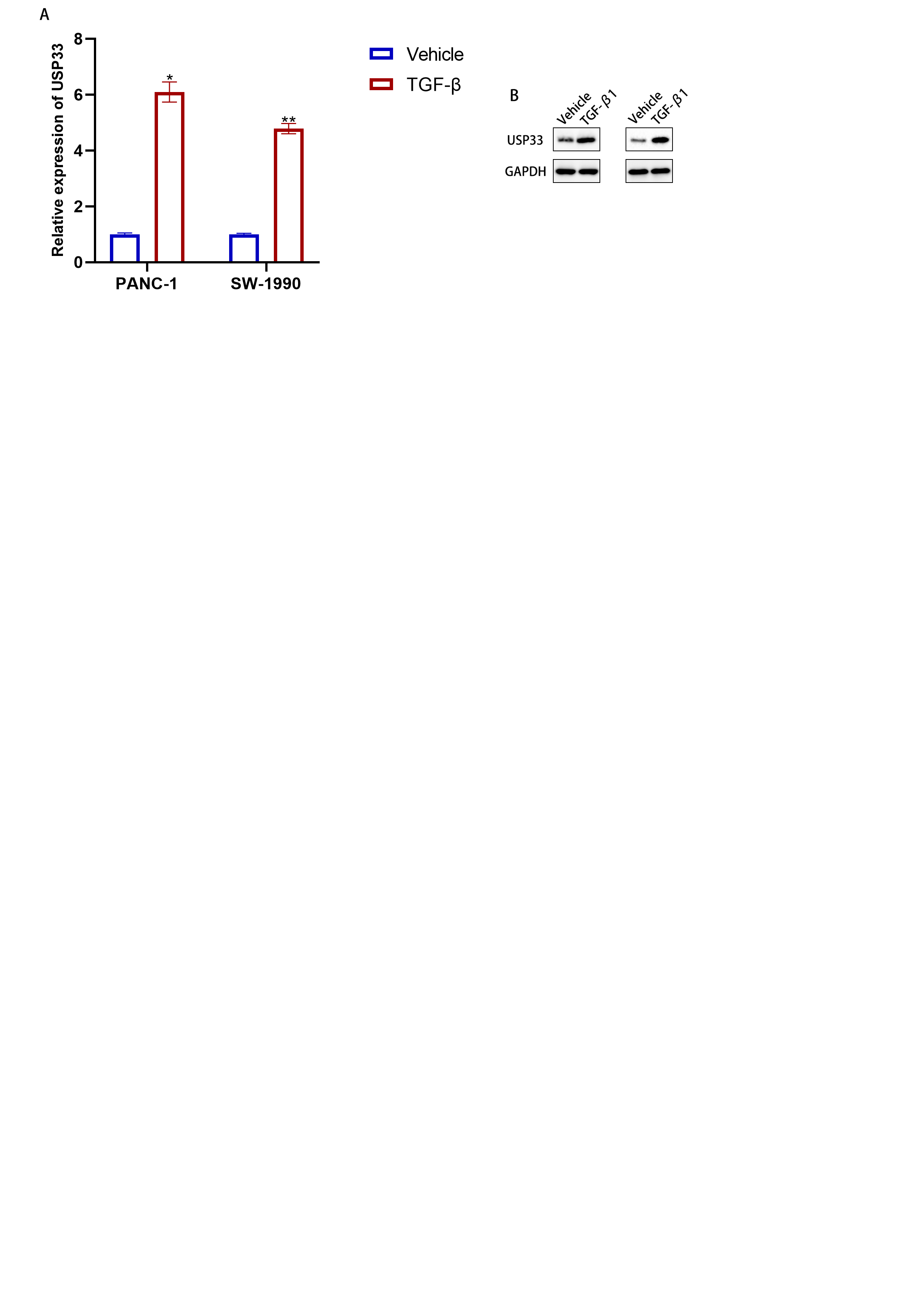

Supplement: Supplementary file 4 — supplementary FIGS3 [file 41419_2023_5871_MOESM4_ESM.tif]

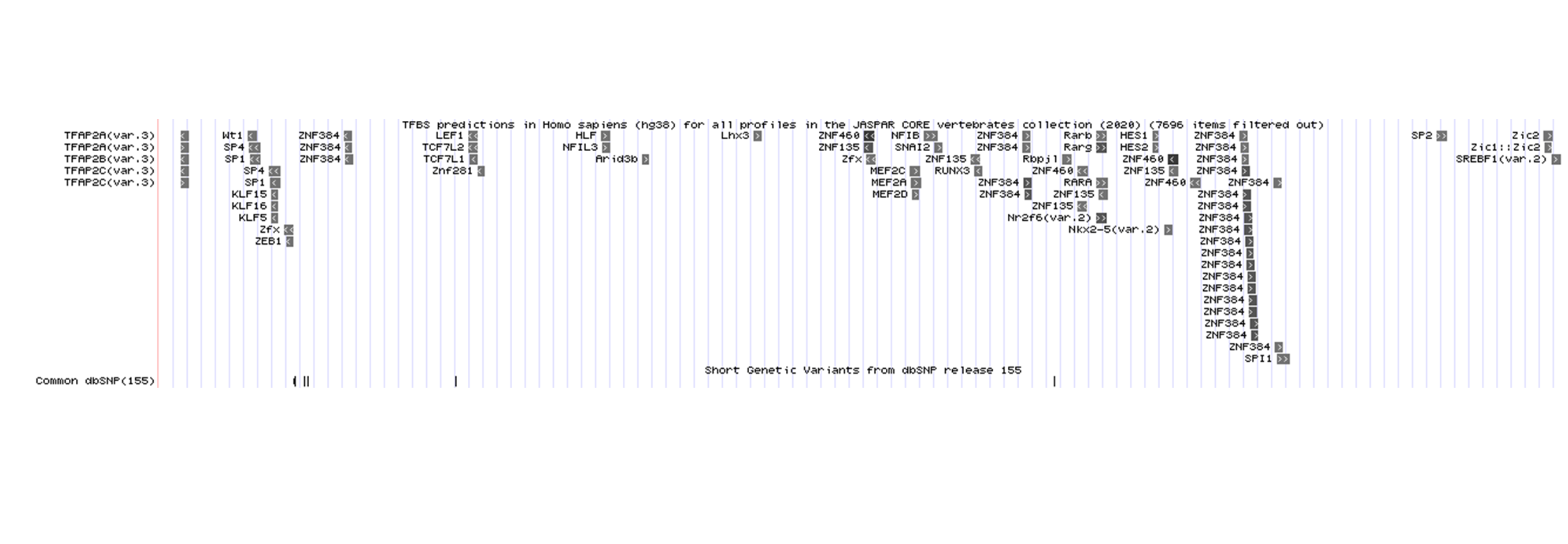

Supplement: Supplementary file 5 — supplementary FIGS4 [file 41419_2023_5871_MOESM5_ESM.tif]

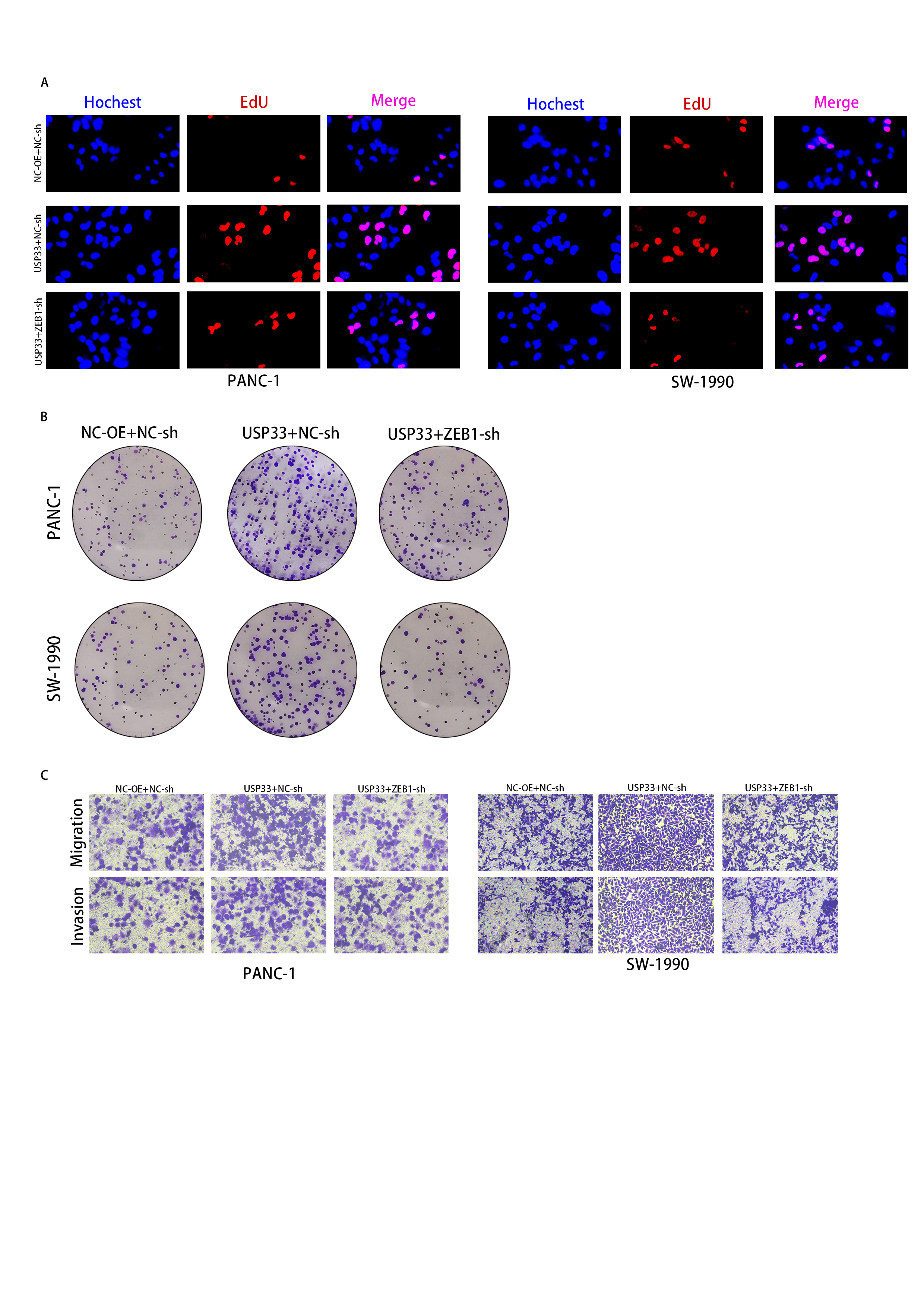

Supplement: Supplementary file 6 — supplementary FIGS5 [file 41419_2023_5871_MOESM6_ESM.tif]
