# Supplementary material for: USP33 promotes pancreatic cancer malignant phenotype through the regulation of TGFBR2/TGFβ signaling pathway
Source: Cell Death Dis. 2023 Jun 15;14(6):362. doi: 10.1038/s41419-023-05871-4 (PMC10272277; doi:10.1038/s41419-023-05871-4)
Supplement: Supplementary file 7 — supplementary table1 [file 41419_2023_5871_MOESM7_ESM.docx]

**ShRNAs used in this research**

| Name | Sequences |
| --- | --- |
| USP33-sh#1 | CCGGGCAACAGTGATAGAGCAGAAACTCGAGTTTCTGCTCTATCACTGTTGCTTTTT |
| USP33-sh#2 | CCGGCTGGATATAGAAGCGGATGAACTCGAGTTCATCCGCTTCTATATCCAGTTTTT |

**Primers used in this research**

| Name | Direction | Sequences(5’-3’) |
| --- | --- | --- |
| USP33 | Forward | AAAATCCCTTGGTACTTGTCAGG |
|  | Reverse | TCGAAGAGTGGTAAGGTTCACA |
| TGFBR2 | Forward |  |
|  | Reverse |  |
| ZEB1 | Forward |  |
|  | Reverse |  |
| GAPDH | Forward | TGTGGGCATCAATGGATTTGG |
|  | Reverse | ACACCATGTATTCCGGGTCAAT |

**Antibodies used in this research**

| Name | Host | Catalogue |
| --- | --- | --- |
| USP33 | Rabbit | 20445-1-AP, Proteintech, Wuhan, China |
| TGFBR2 | Mouse | 66636-1-Ig, Proteintech, Wuhan, China |
| ZEB1 | Mouse | 66279-1-Ig, Proteintech, Wuhan, China |
| Snai1 | Rabbit | A5243, ABclonal, Wuhan, China |
| Twist1 | Rabbit | A7314, ABclonal, Wuhan, China |
| MMP9 | Rabbit | A0289, ABclonal, Wuhan, China |
| MMP12 | Rabbit | A3713, ABclonal, Wuhan, China |
| N-Cadherin | Rabbit | ab76011, Abcam |
| E-Cadherin | Rabbit | ab181296, Abcam |
| GAPDH | Rabbit | 10494-1-AP, Proteintech, Wuhan, China |
| β-Actin | Rabbit | 20536-1-AP, Proteintech, Wuhan, China |
| Anti-Rabbit IgG | / | AS014, ABclonal, Wuhan, China |
| Anti-Mouse IgG | / | AS003, ABclonal, Wuhan, China |
