# Supplementary material for: USP33 promotes pancreatic cancer malignant phenotype through the regulation of TGFBR2/TGFβ signaling pathway
Source: Cell Death Dis. 2023 Jun 15;14(6):362. doi: 10.1038/s41419-023-05871-4 (PMC10272277; doi:10.1038/s41419-023-05871-4)
Supplement: Supplementary file 8 — supplementary table2 [file 41419_2023_5871_MOESM8_ESM.docx]

**Relationship between USP33 expression and clinicopathological features**

| **Parameters** | **n** | **USP33 expression**  **Low expression** | **High expression** | **P value** |
| --- | --- | --- | --- | --- |
| **Age (years)** |  |  |  |  |
| ≤60 | 20 | 9 | 11 |  |
| >60 | 26 | 14 | 12 | 0.5519 |
| **Sex** |  |  |  |  |
| Female | 33 | 16 | 17 |  |
| Male | 13 | 6 | 7 | 0.8867 |
| **T classification** |  |  |  |  |
| T1–T2 | 17 | 12 | 5 |  |
| T3–T4 | 29 | 9 | 20 | 0.0093 |
| **N classification** |  |  |  |  |
| N0 | 26 | 12 | 14 |  |
| N1-N2 | 20 | 5 | 15 | 0.1406 |
| **Distant metastasis** |  |  |  |  |
| No | 30 | 11 | 19 |  |
| Yes | 16 | 5 | 11 | 0.7133 |
| **Histologic grade** |  |  |  |  |
| High | 16 | 10 | 6 |  |
| Low to medium | 30 | 9 | 21 | 0.0330 |
| **Differentiation** |  |  |  |  |
| Well | 11 | 5 | 6 |  |
| Moderate/poor | 35 | 10 | 25 | 0.2974 |
| **Survival state** |  |  |  |  |
| Live | 34 | 13 | 21 |  |
| Dead | 12 | 10 | 2 | 0.0072 |
