# Supplementary material for: USP33 promotes pancreatic cancer malignant phenotype through the regulation of TGFBR2/TGFβ signaling pathway
Source: Cell Death Dis. 2023 Jun 15;14(6):362. doi: 10.1038/s41419-023-05871-4 (PMC10272277; doi:10.1038/s41419-023-05871-4)
Supplement: Supplementary file 9 — supplementary figure legend [file 41419_2023_5871_MOESM9_ESM.docx]

**FigS1**

**USP33 promoted the expression of TGFB targeted genes**

A. The western-blotting results showed the effect of USP33 knockdown on the expression of TGFB associated genes PAI-I, VEGF and TGFBR1. B. The western-blotting results showed the effect of USP33 on the TGFβ1-mdiated TGFBR1 activation.

**FigS2**

**USP33 promoted the recycling of TGFBR2 in PC cells**

A. After incubated the PANC-1 cells with cell membrane marker DIO or TGFBR2 antibodies at 4℃, we switched the temperature to 37 °C for indicated time to observe the internalization of TGFBR2, the results were visualized by confocal laser scanning microscope.

**FigS3**

**TGFβ signaling promoted the expression of USP33**

A. The qRT-PCR result showed the effect of TGFβ1 stimulation on the expression of USP33 mRNA. B. The western-blotting result showed the effect of TGFβ1 stimulation on the expression of USP33 protein.

**FigS4**

**ZEB1 was the candidate of USP33 transcriptional factor**

The JASPAR database screened the putative transcriptional factor of USP33 and ZEB1 was included.

**FigS5**

**USP33 promoted the malignant phenotype of PC cells in a ZEB1 dependent manner**

A. The EdU assay of PC cells co-transfected with USP33 overexpression plasmids and ZEB1 shRNA. B. The colony formation of PC cells co-transfected with USP33 overexpression plasmids and ZEB1 shRNA. C. The transwell experiment of PC cells co-transfected with indicated plasmids.
