# Supplementary material for: USP33 promotes pancreatic cancer malignant phenotype through the regulation of TGFBR2/TGFβ signaling pathway
Source: Cell Death Dis. 2023 Jun 15;14(6):362. doi: 10.1038/s41419-023-05871-4 (PMC10272277; doi:10.1038/s41419-023-05871-4)
Supplement: Supplementary file 10 — Original Data File [file 41419_2023_5871_MOESM10_ESM.pdf]

Fig1E

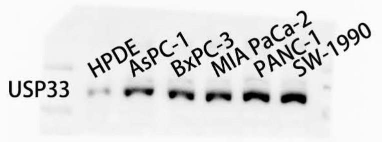

Fig1E

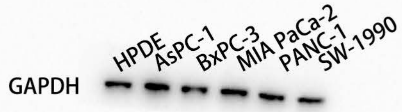

Fig1H

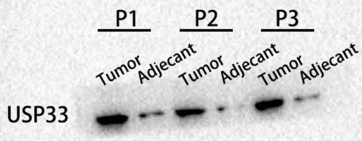

Fig1H

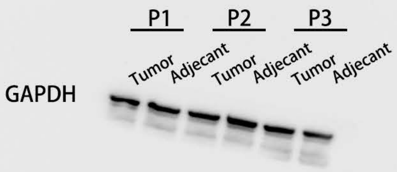

Fig1H

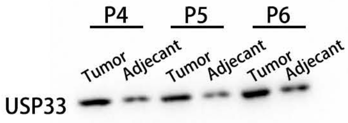

Fig1H

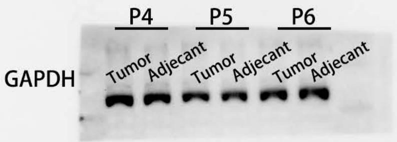

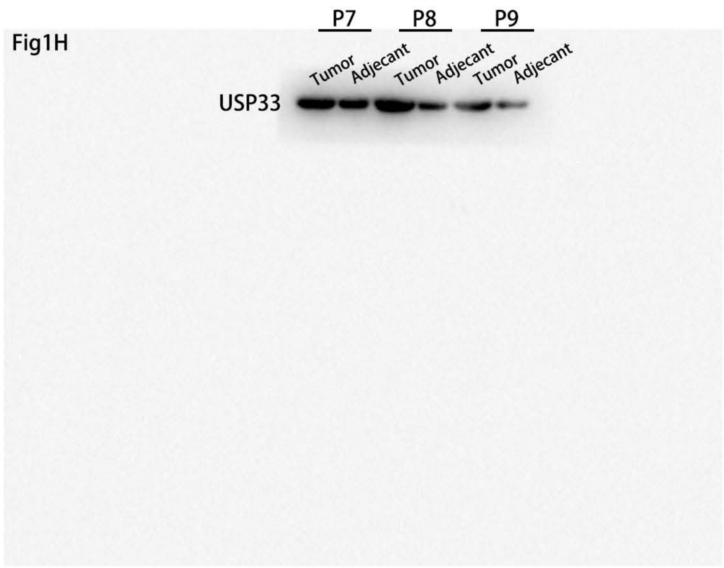
Fig1H
P7
P8
P9
Tumor
Adjecant
Tumor
Adjecant
Tumor
Adjecant
USP33

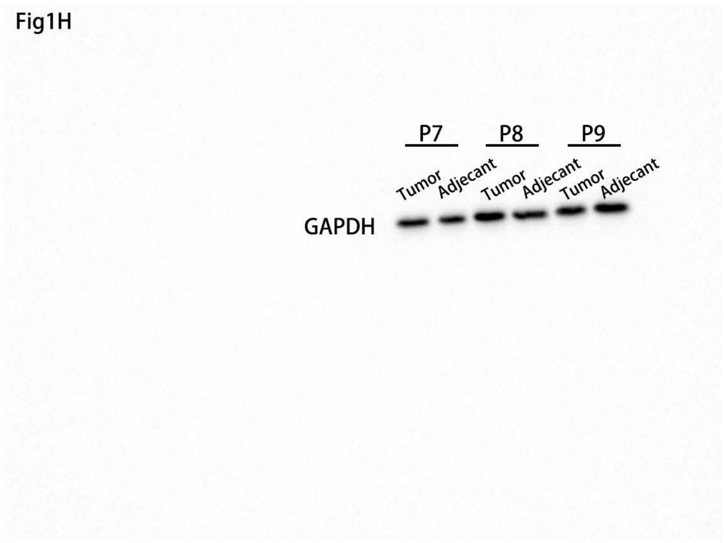
Fig1H
P7
P8
P9
Tumor
Adjecant
Tumor
Adjecant
Tumor
Adjecant
GAPDH

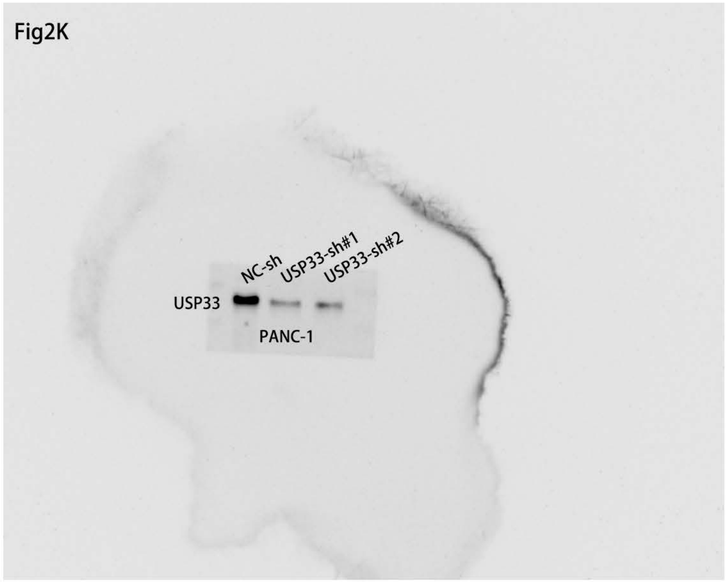
Fig2K
NC-sh
USP33-sh#1
USP33-sh#2
USP33
PANC-1

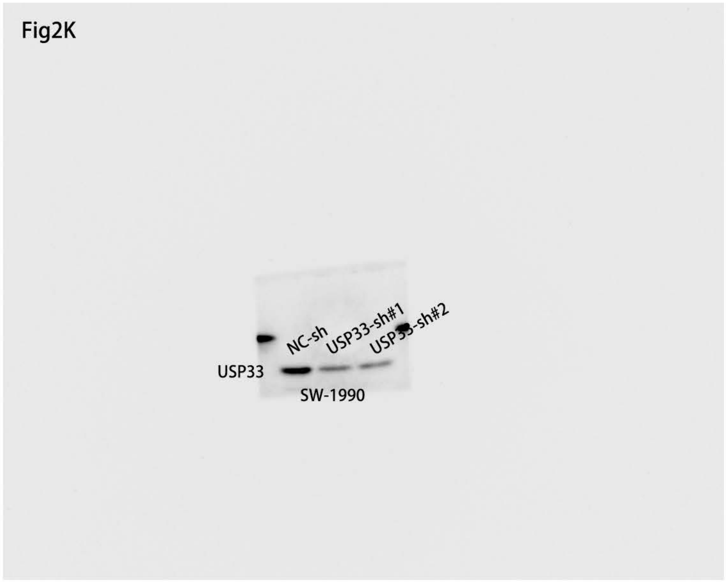
Fig2K
NC-sh
USP33-sh#1
USP33-sh#2
USP33
SW-1990

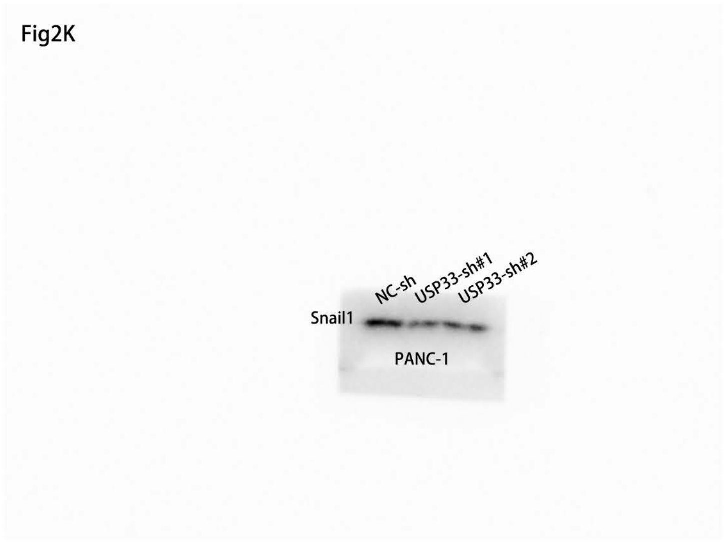
Fig2K
NC-sh
USP33-sh#1
USP33-sh#2
Snail1
PANC-1

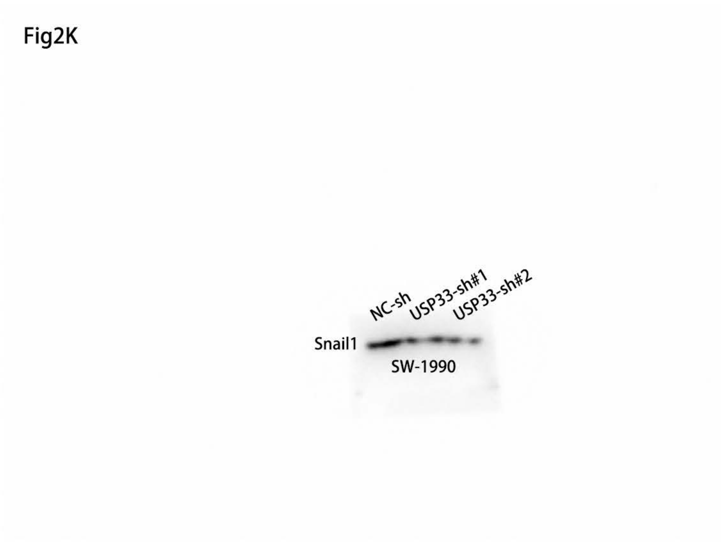
Fig2K
NC-sh
USP33-sh#1
USP33-sh#2
Snail1
SW-1990

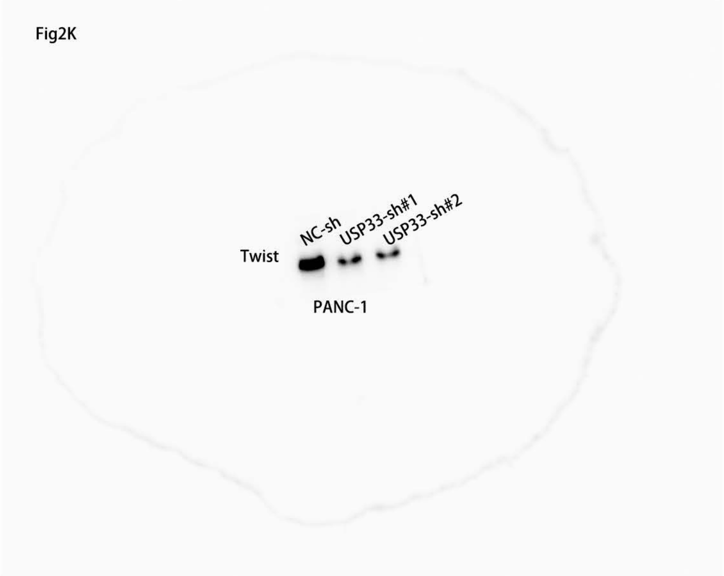
Fig2K
NC-sh
USP33-sh#1
USP33-sh#2
Twist
PANC-1

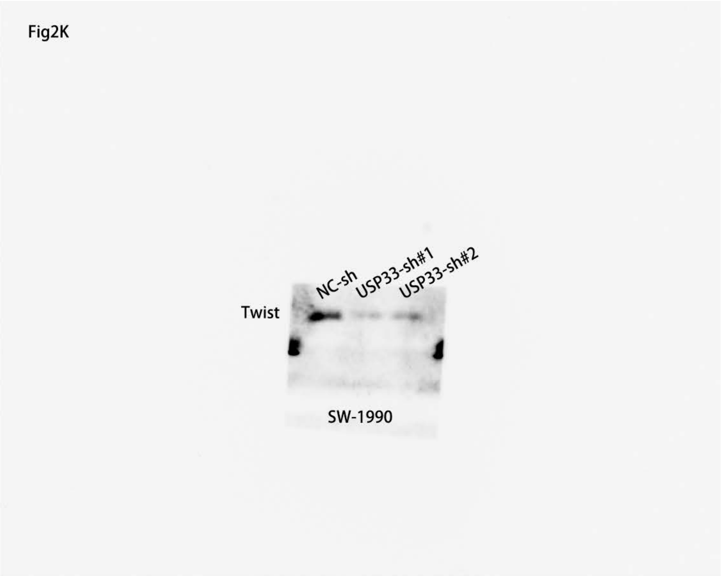
Fig2K
NC-sh
USP33-sh#1
USP33-sh#2
Twist
SW-1990

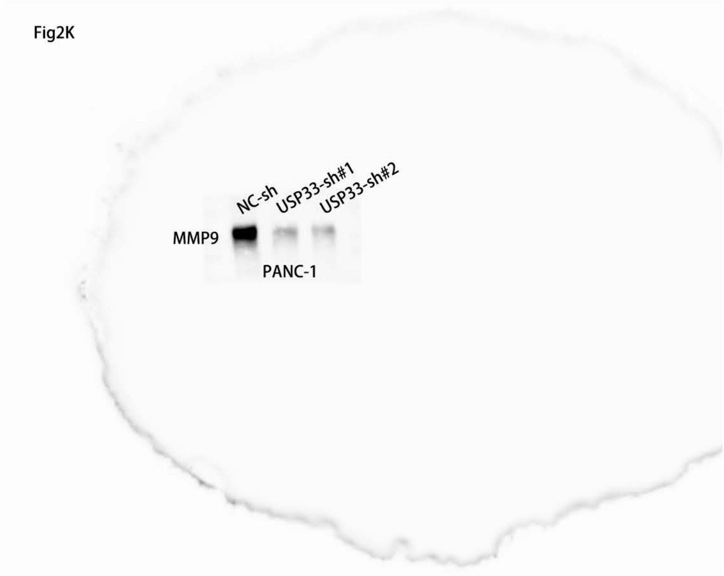
Fig2K
NC-sh
USP33-sh#1
USP33-sh#2
MMP9
PANC-1

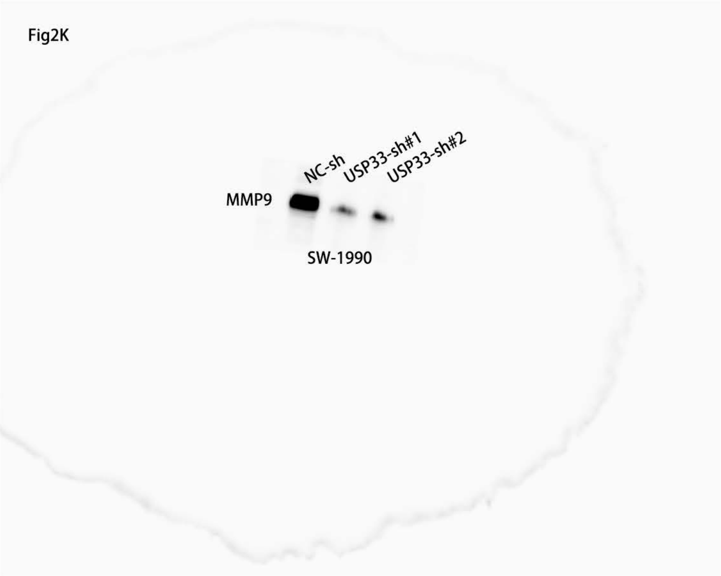
Fig2K
NC-sh
USP33-sh#1
USP33-sh#2
MMP9
SW-1990

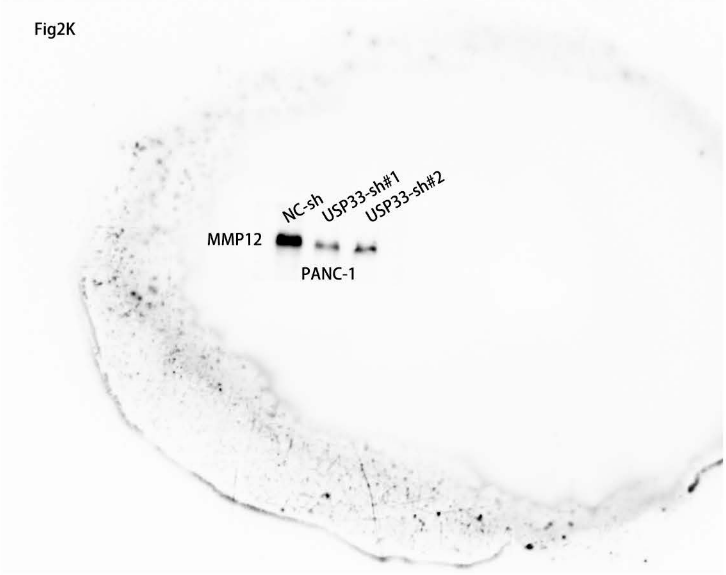
Fig2K
NC-sh
USP33-sh#1
USP33-sh#2
MMP12
PANC-1

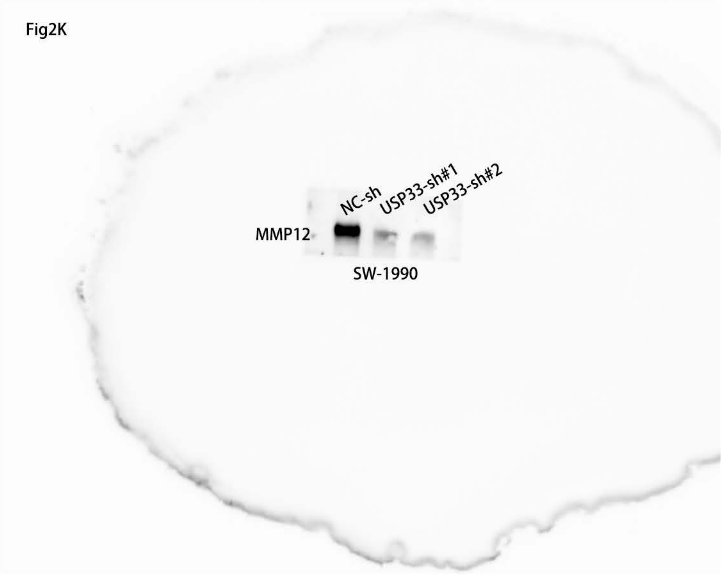
Fig2K
NC-sh
USP33-sh#1
USP33-sh#2
MMP12
SW-1990

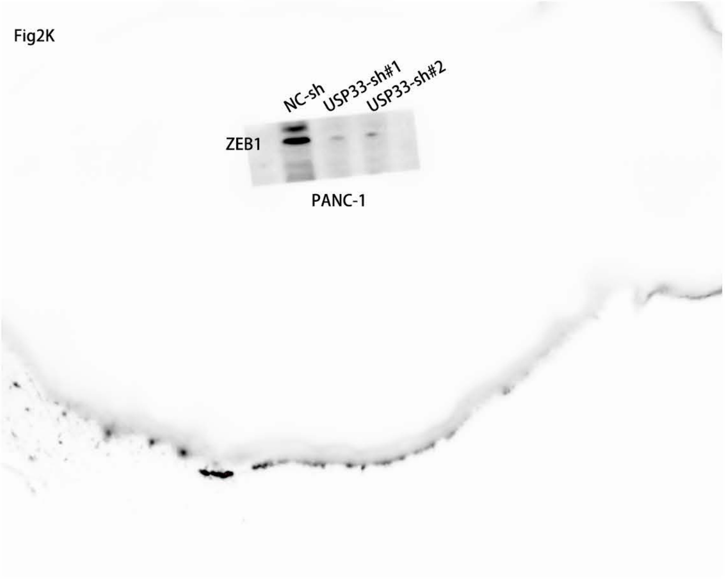
Fig2K
NC-sh
USP33-sh#1
USP33-sh#2
ZEB1
PANC-1

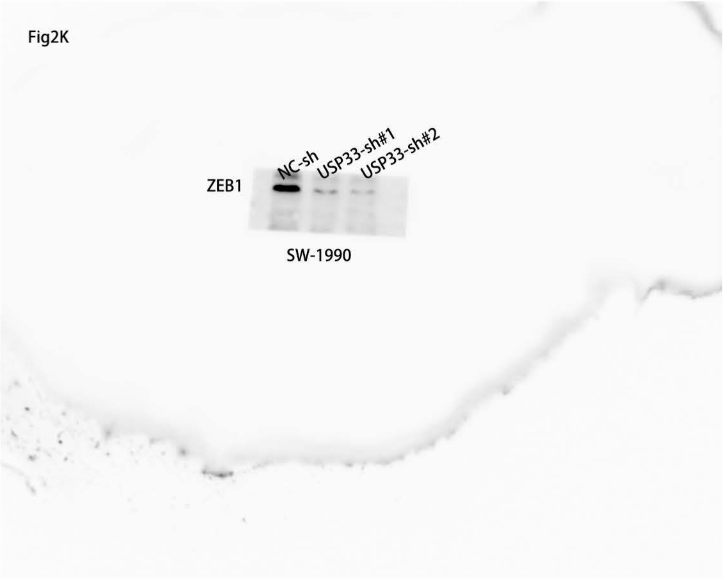
Fig2K
NC-sh
USP33-sh#1
USP33-sh#2
ZEB1
SW-1990

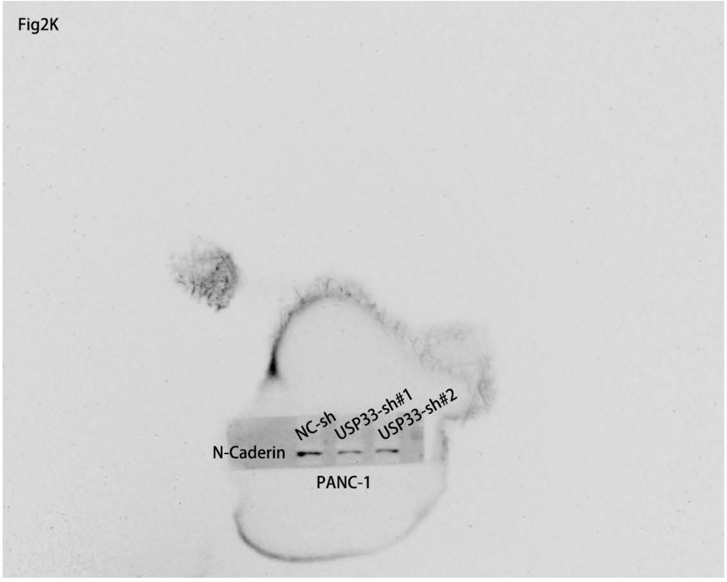
Fig2K
NC-sh
USP33-sh#1
USP33-sh#2
N-Caderin
PANC-1

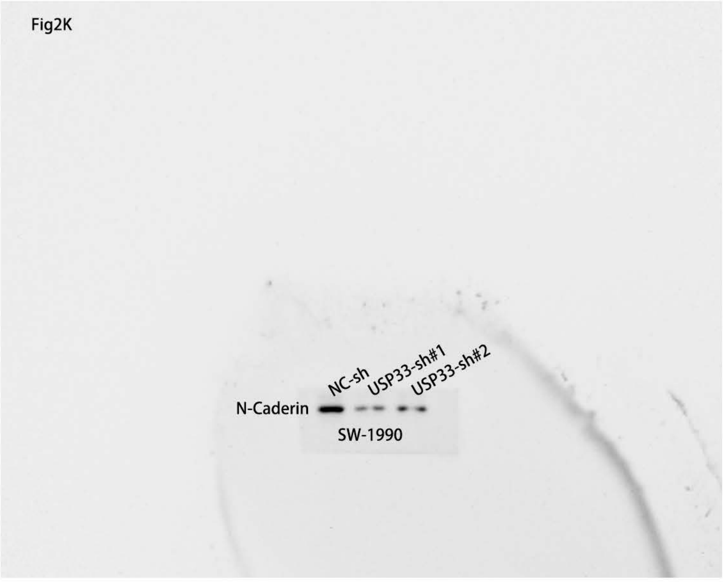
Fig2K
NC-sh
USP33-sh#1
USP33-sh#2
N-Caderin
SW-1990

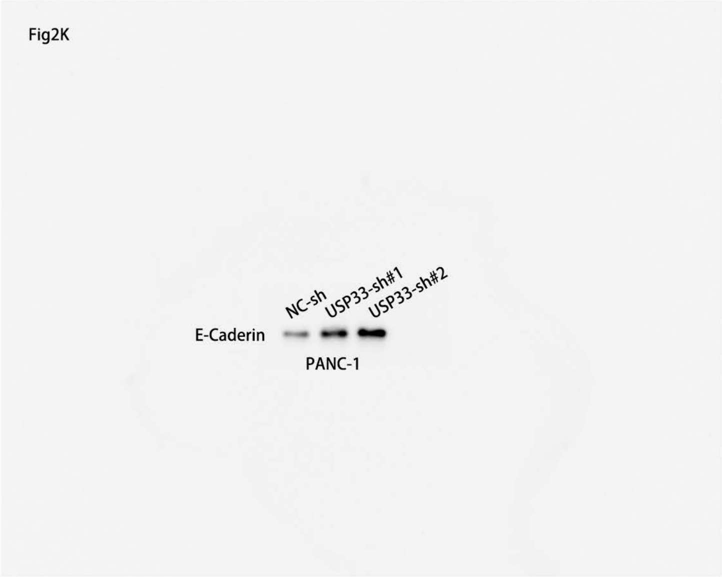
Fig2K
NC-sh
USP33-sh#1
USP33-sh#2
E-Caderin
PANC-1

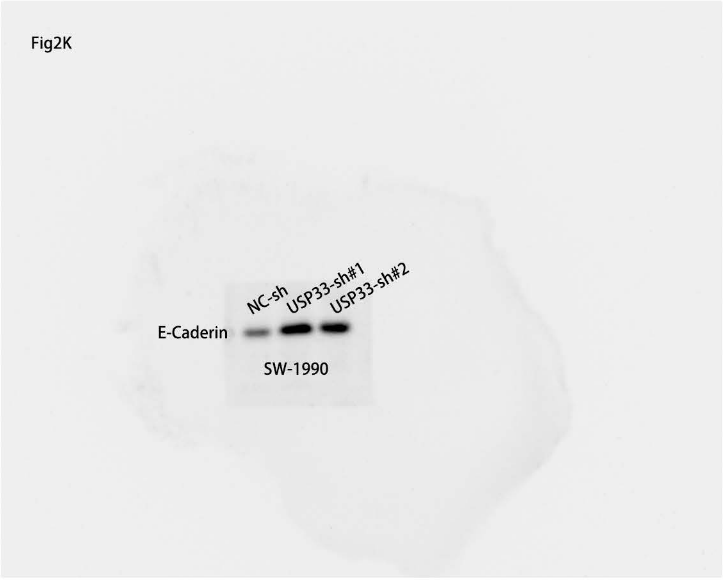
Fig2K
NC-sh
USP33-sh#1
USP33-sh#2
E-Caderin
SW-1990

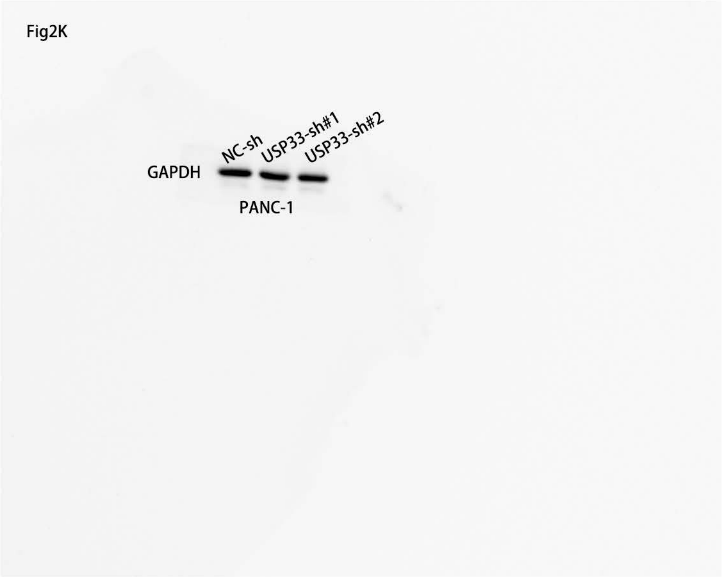
Fig2K
NC-sh
USP33-sh#1
USP33-sh#2
GAPDH
PANC-1

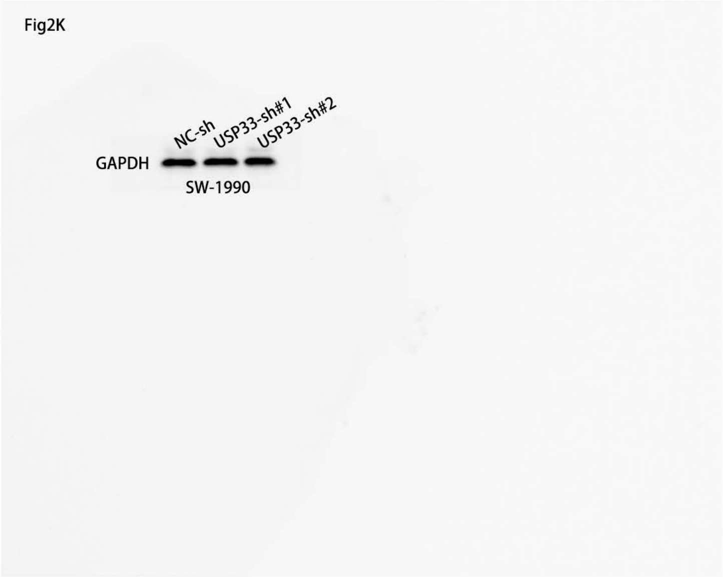
Fig2K
NC-sh
USP33-sh#1
USP33-sh#2
GAPDH
SW-1990

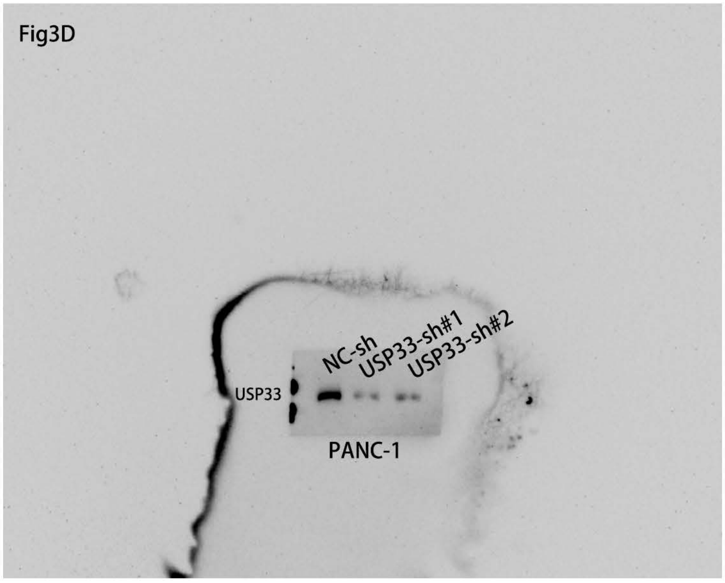
Fig3D
NC-sh
USP33-sh#1
USP33-sh#2
USP33
PANC-1

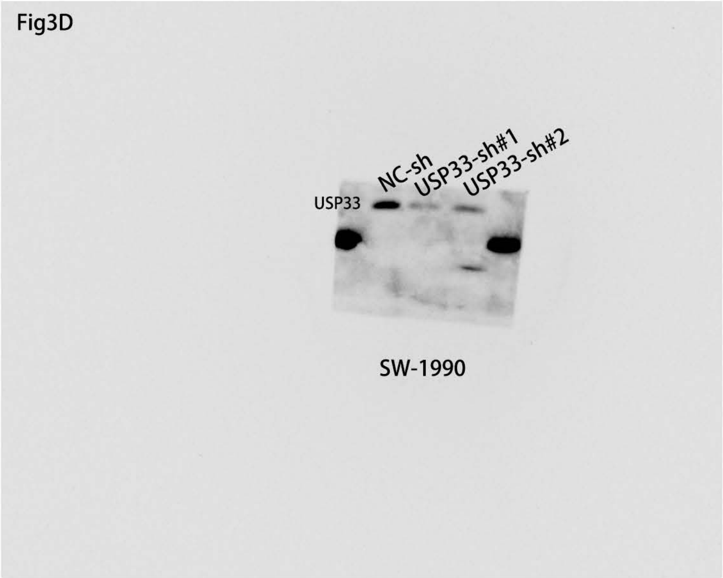
Fig3D
NC-sh
USP33-sh#1
USP33-sh#2
USP33
SW-1990

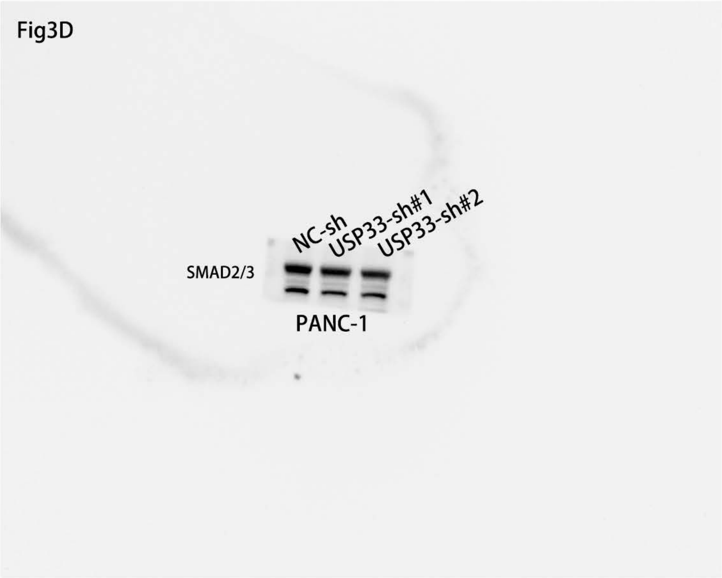
Fig3D
NC-sh
USP33-sh#1
USP33-sh#2
SMAD2/3
PANC-1

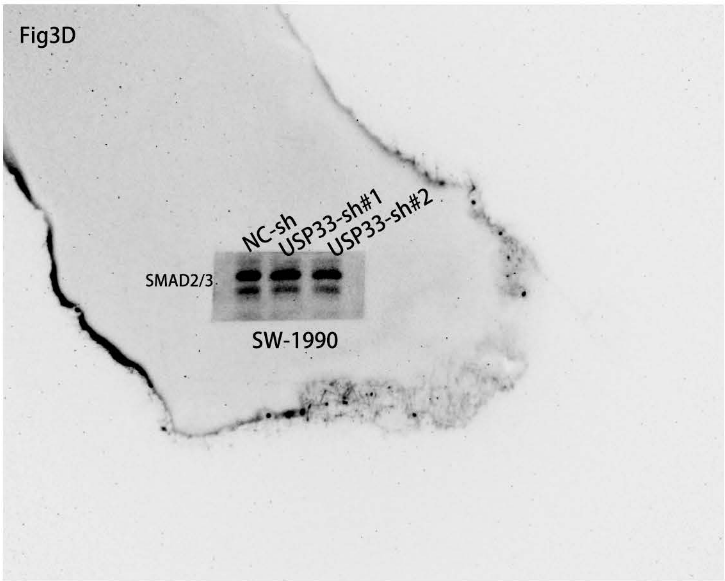
Fig3D
NC-sh
USP33-sh#1
USP33-sh#2
SMAD2/3
SW-1990

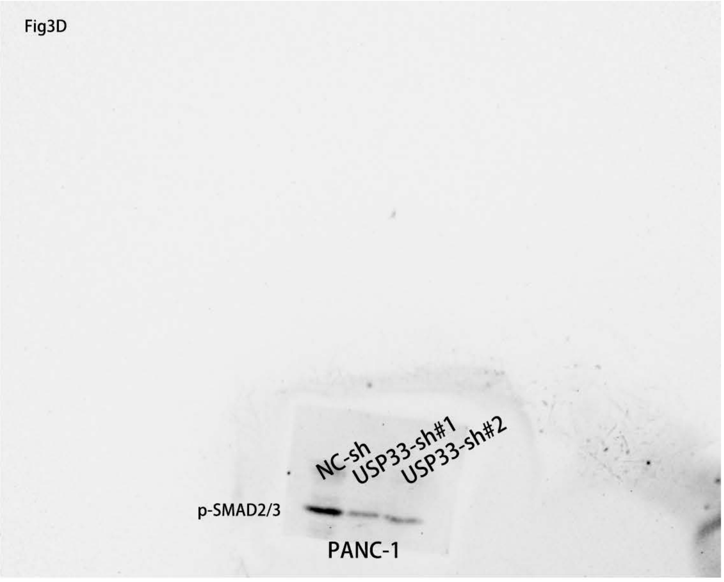
Fig3D
NC-sh
USP33-sh#1
USP33-sh#2
p-SMAD2/3
PANC-1

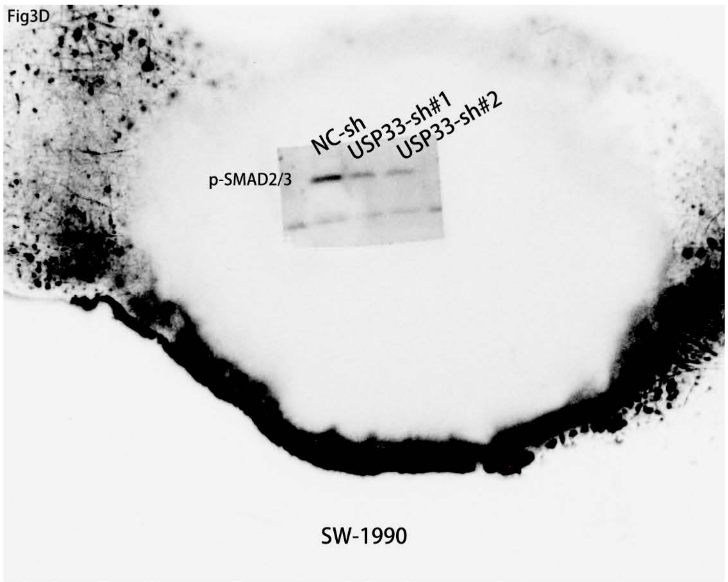
Fig3D
NC-sh
USP33-sh#1
USP33-sh#2
p-SMAD2/3
SW-1990

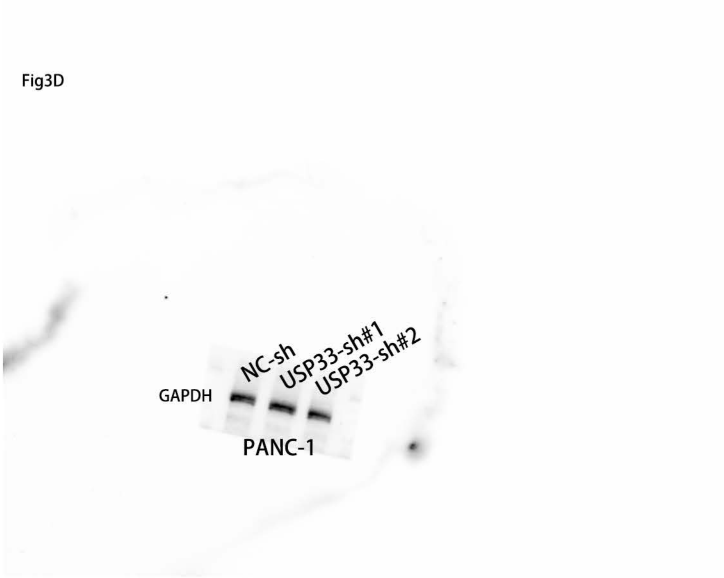
Fig3D
NC-sh
USP33-sh#1
USP33-sh#2
GAPDH
PANC-1

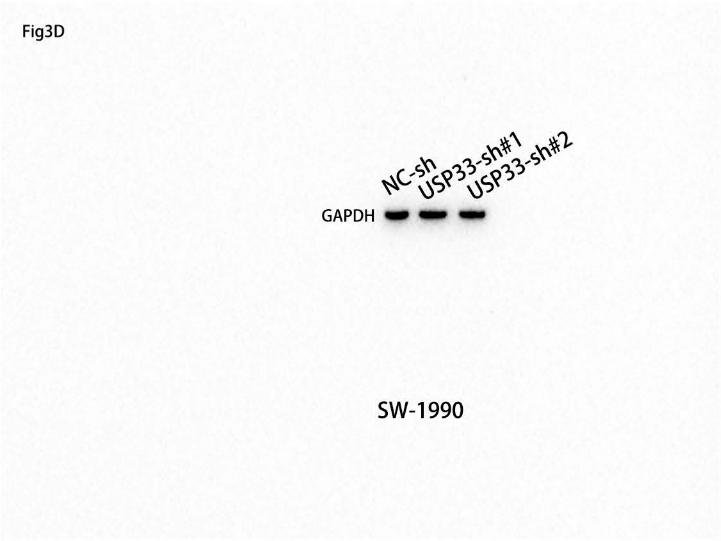
Fig3D
NC-sh
USP33-sh#1
USP33-sh#2
GAPDH
SW-1990

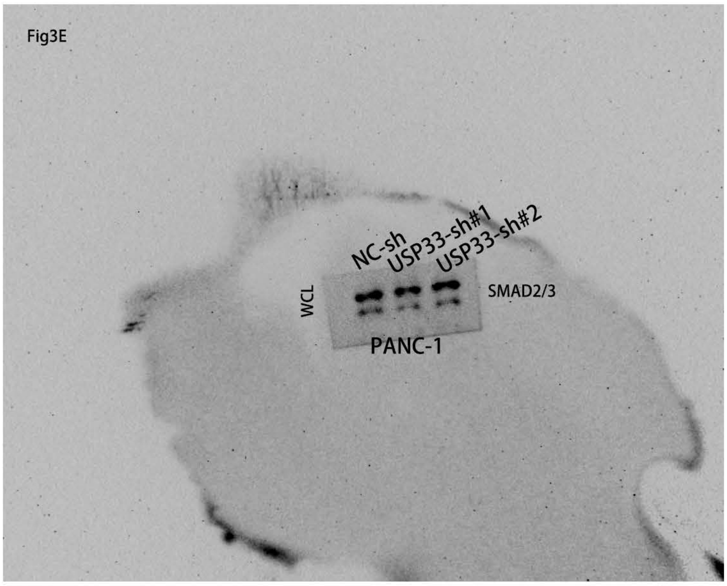
Fig3E
NC-sh
USP33-sh#1
USP33-sh#2
WCL
SMAD2/3
PANC-1

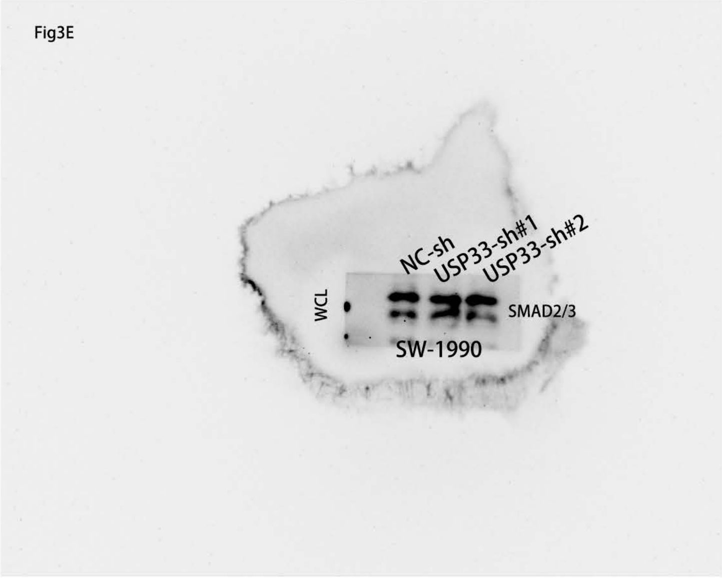
Fig3E
NC-sh
USP33-sh#1
USP33-sh#2
WCL
SMAD2/3
SW-1990

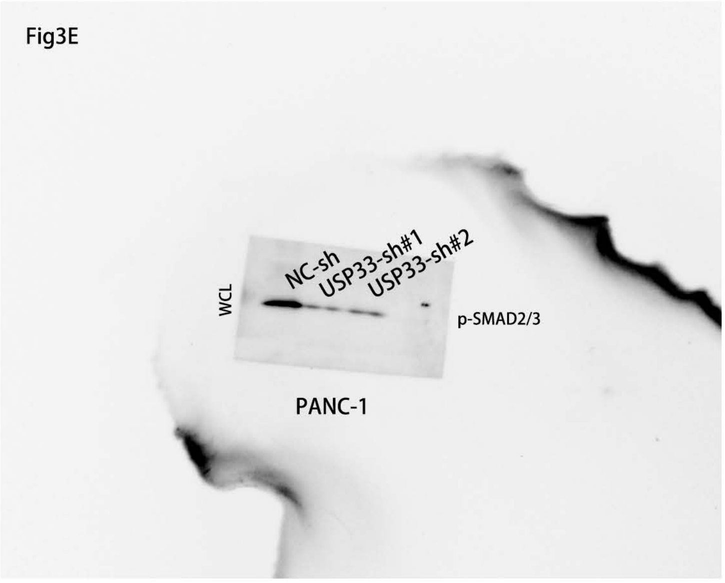
Fig3E
NC-sh
USP33-sh#1
USP33-sh#2
WCL
p-SMAD2/3
PANC-1

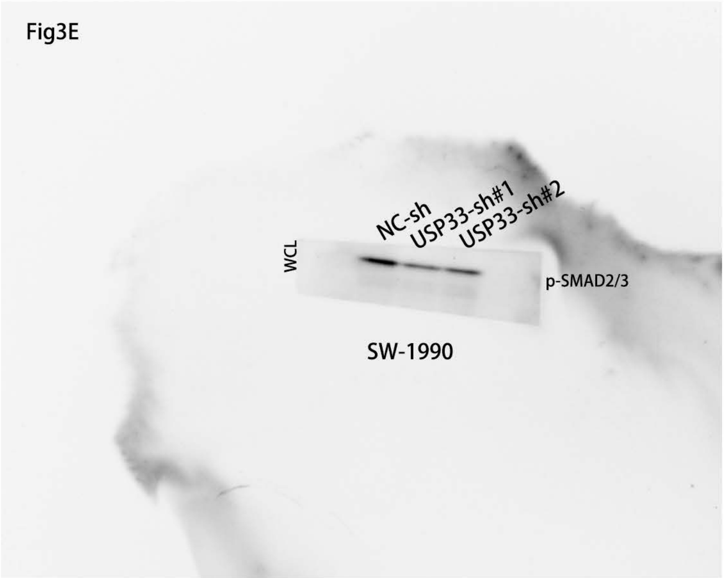
Fig3E
NC-sh
USP33-sh#1
USP33-sh#2
WCL
p-SMAD2/3
SW-1990

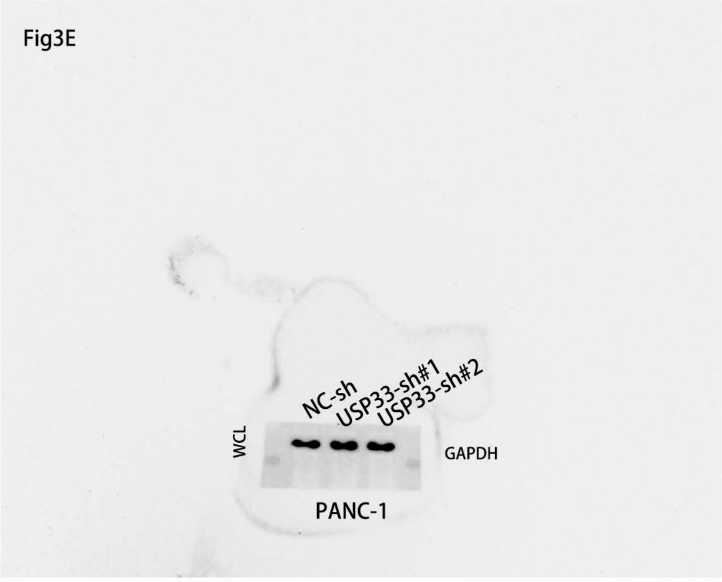
Fig3E
NC-sh
USP33-sh#1
USP33-sh#2
WCL
GAPDH
PANC-1

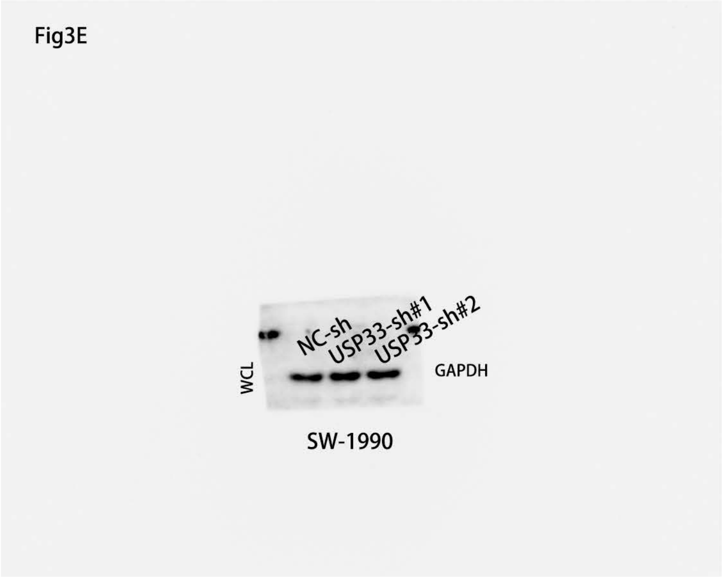
Fig3E
NC-sh
USP33-sh#1
USP33-sh#2
WCL
GAPDH
SW-1990

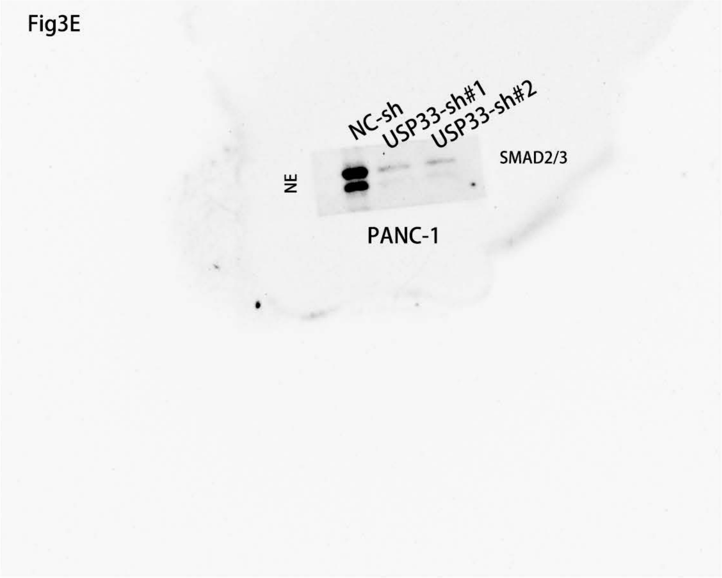
Fig3E
NC-sh
USP33-sh#1
USP33-sh#2
NE
SMAD2/3
PANC-1

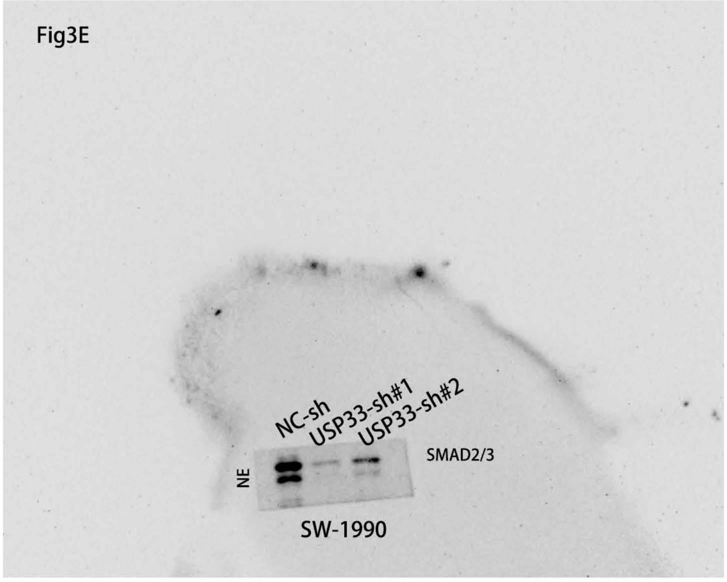
Fig3E
NC-sh
USP33-sh#1
USP33-sh#2
NE
SMAD2/3
SW-1990

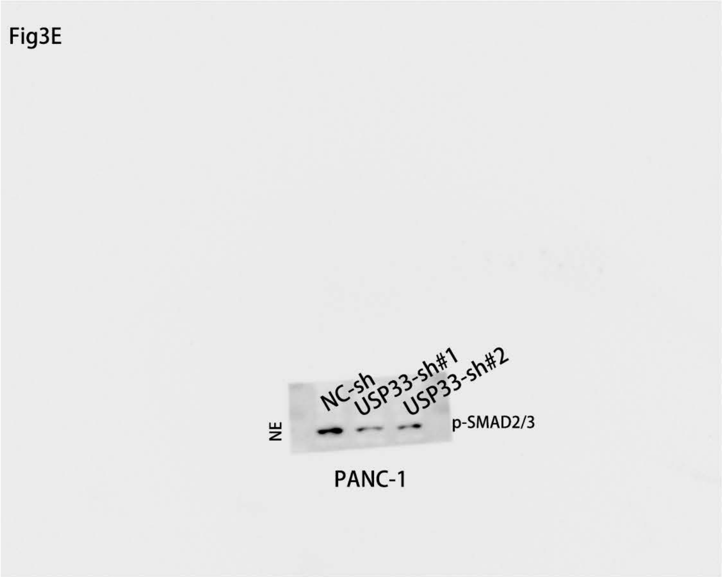
Fig3E
NC-sh
USP33-sh#1
USP33-sh#2
NE
p-SMAD2/3
PANC-1

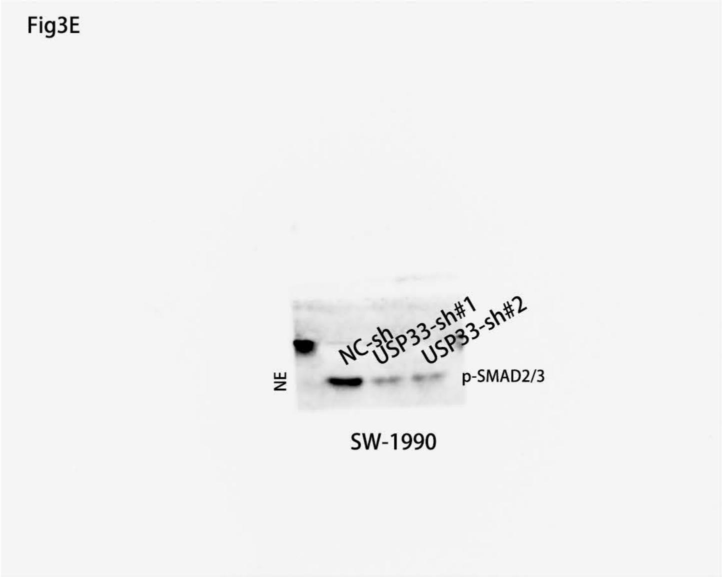
Fig3E
NC-sh
USP33-sh#1
USP33-sh#2
NE
p-SMAD2/3
SW-1990

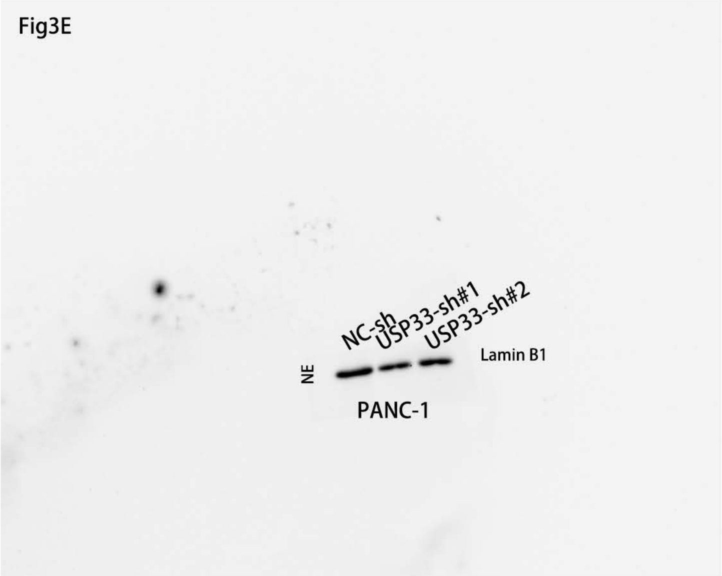
Fig3E
NC-sh
USP33-sh#1
USP33-sh#2
NE
Lamin B1
PANC-1

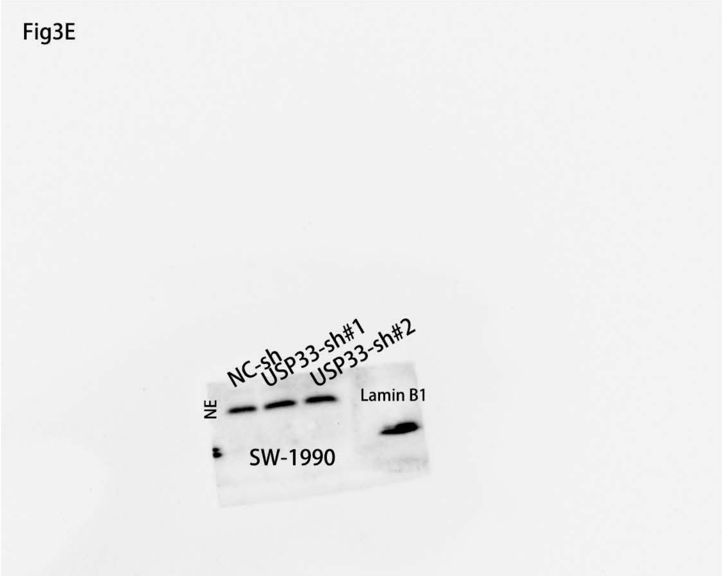
Fig3E
NC-sh
USP33-sh#1
USP33-sh#2
NE
Lamin B1
SW-1990

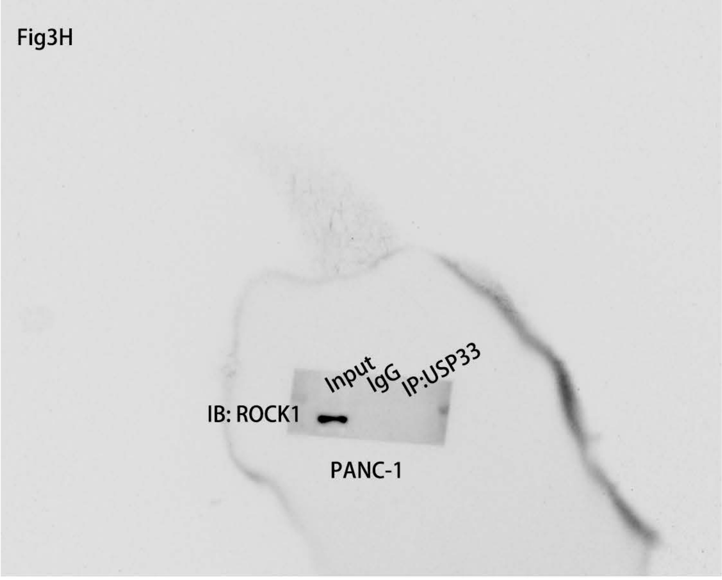
Fig3H
Input
IgG
IP:USP33
IB: ROCK1
PANC-1

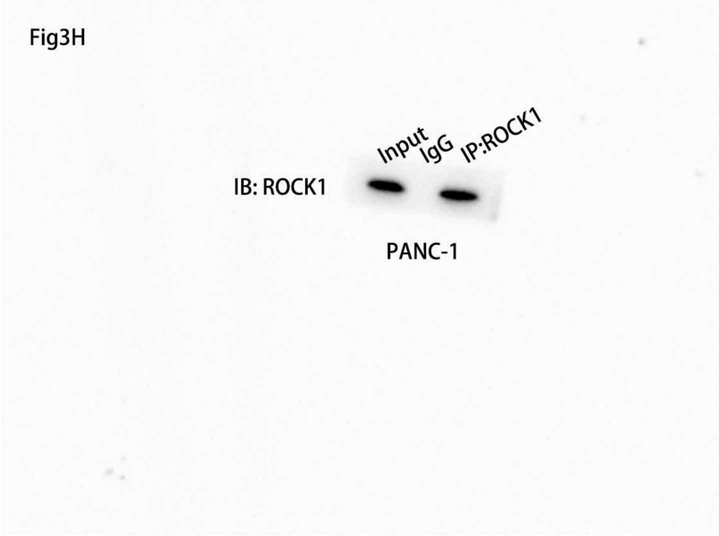
Fig3H
Input
IgG
IP:ROCK1
IB: ROCK1
PANC-1

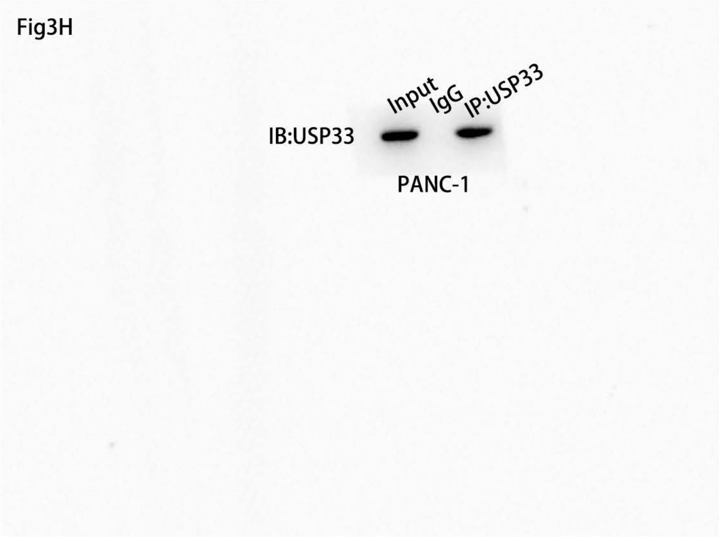
Fig3H
Input
IgG
IP:USP33
IB:USP33
PANC-1

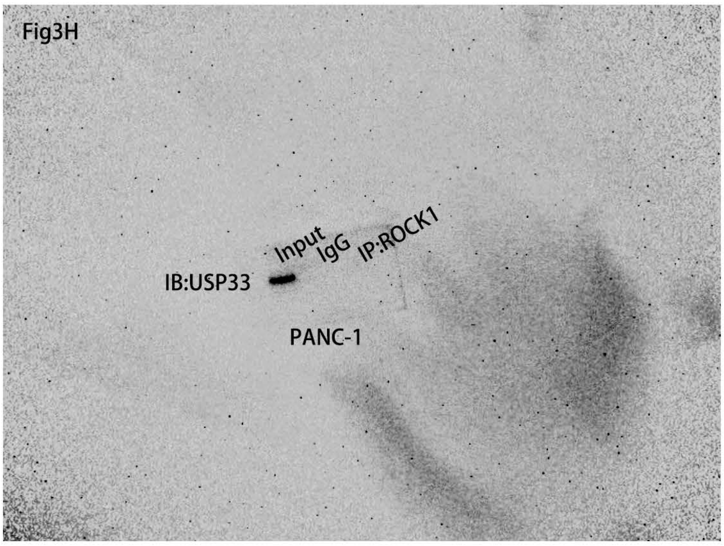
Fig3H
Input
IgG
IP:ROCK1
IB:USP33
PANC-1

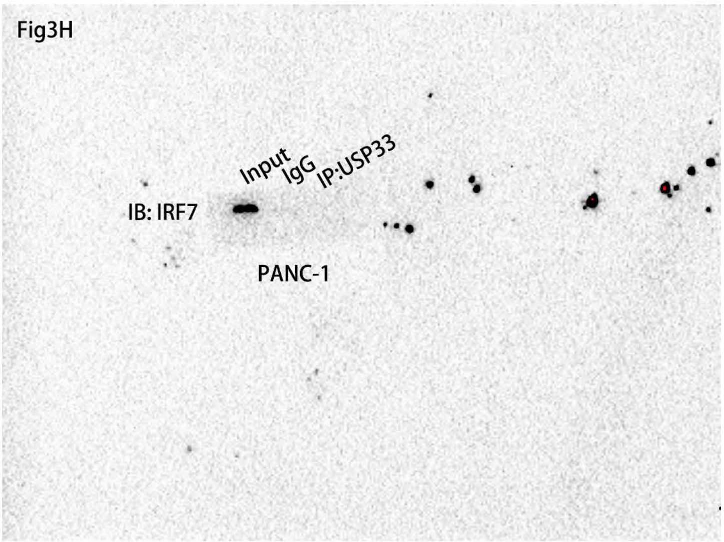
Fig3H
Input
IgG
IP:USP33
IB: IRF7
PANC-1

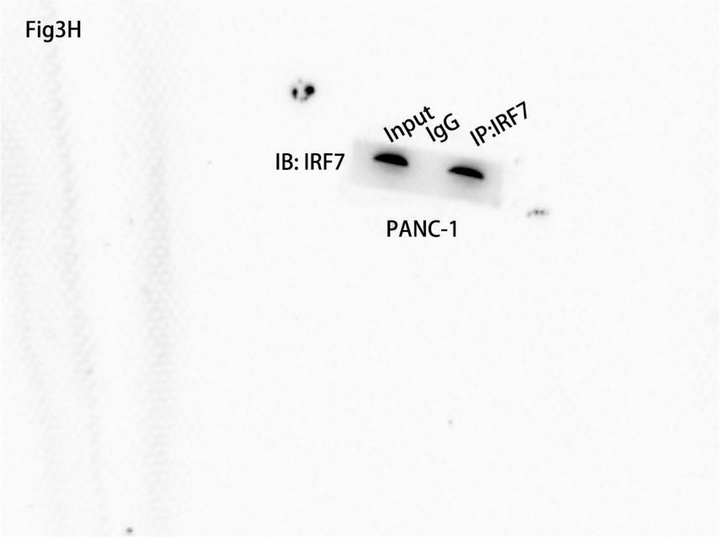
Fig3H
Input
IgG
IP:IRF7
IB: IRF7
PANC-1

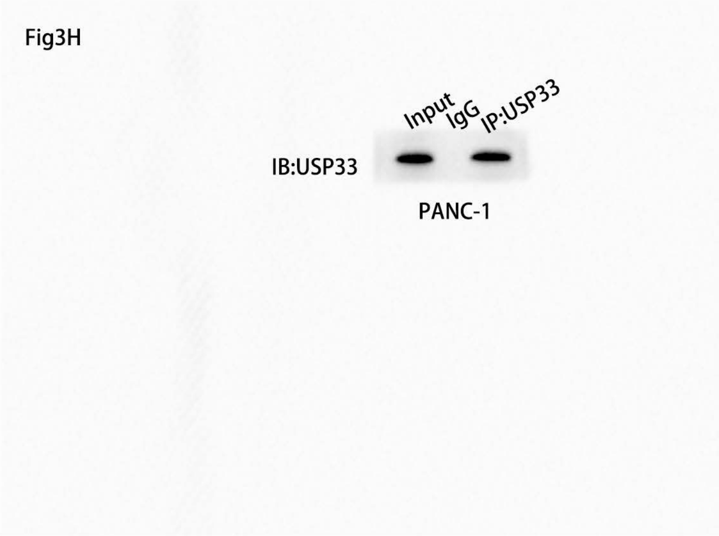
Fig3H
Input
IgG
IP:USP33
IB:USP33
PANC-1

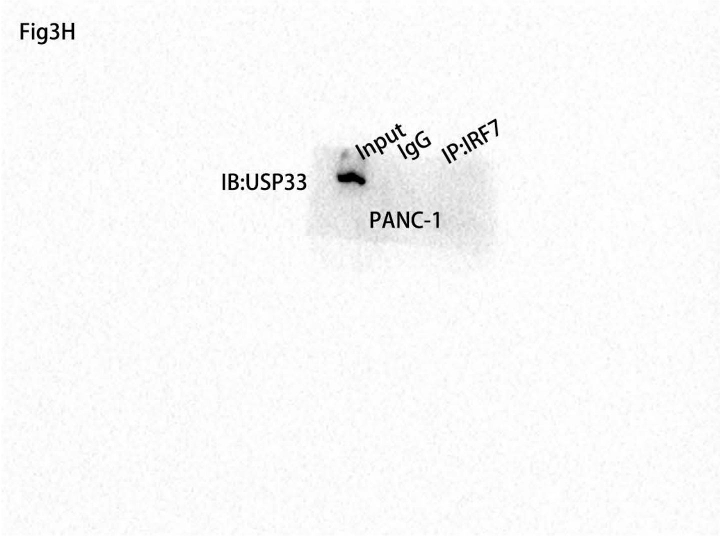
Fig3H
Input
IgG
IP:IRF7
IB:USP33
PANC-1

Fig3H

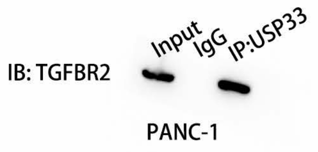

Fig3H

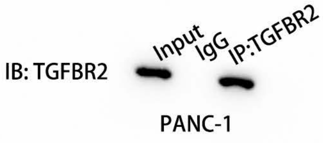

Fig3H

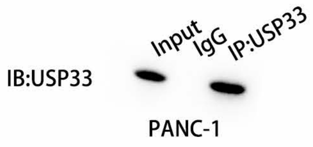

Fig3H

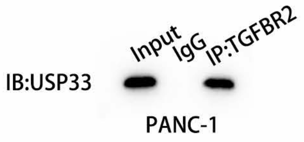

Fig3H

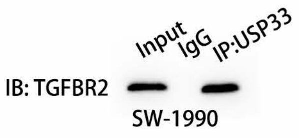

Fig3H

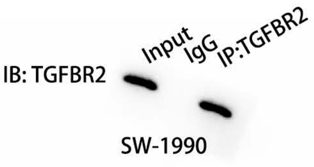

Fig3H

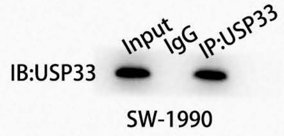

Fig3H

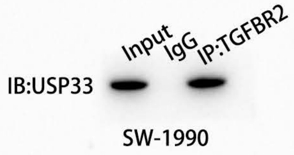

Fig3K

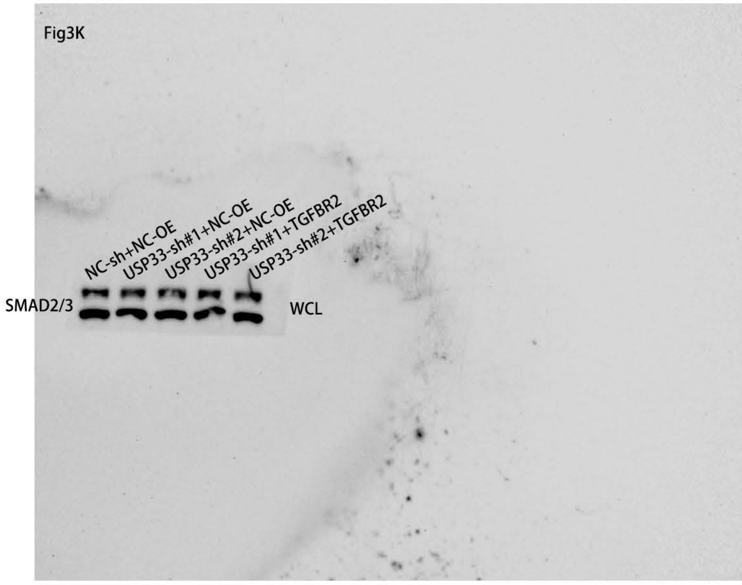

Fig3K

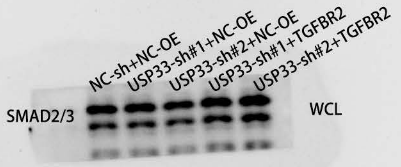

Fig3K

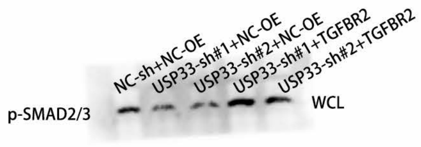

Fig3K

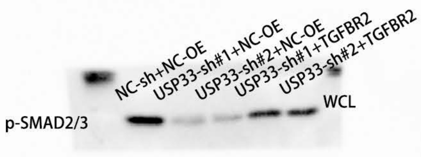

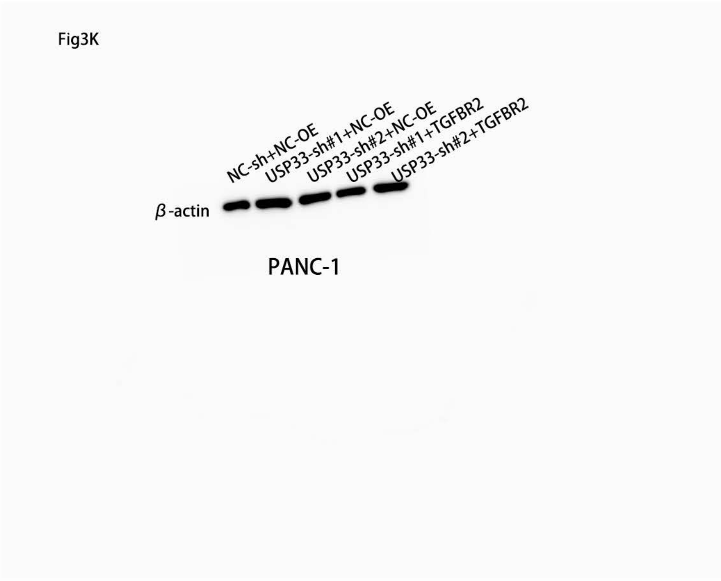
Fig3K
NC-sh+NC-OE
USP33-sh#1+NC-OE
USP33-sh#2+NC-OE
USP33-sh#1+TGFBR2
USP33-sh#2+TGFBR2
β-actin
PANC-1

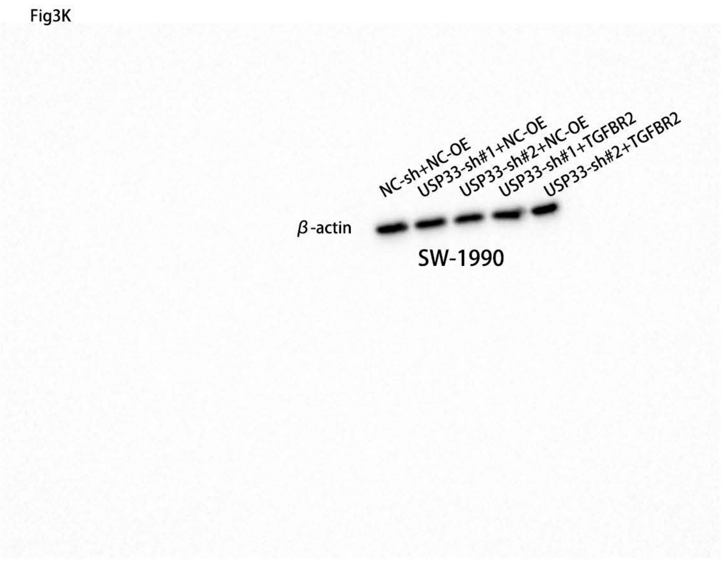
Fig3K
NC-sh+NC-OE
USP33-sh#1+NC-OE
USP33-sh#2+NC-OE
USP33-sh#1+TGFBR2
USP33-sh#2+TGFBR2
β-actin
SW-1990

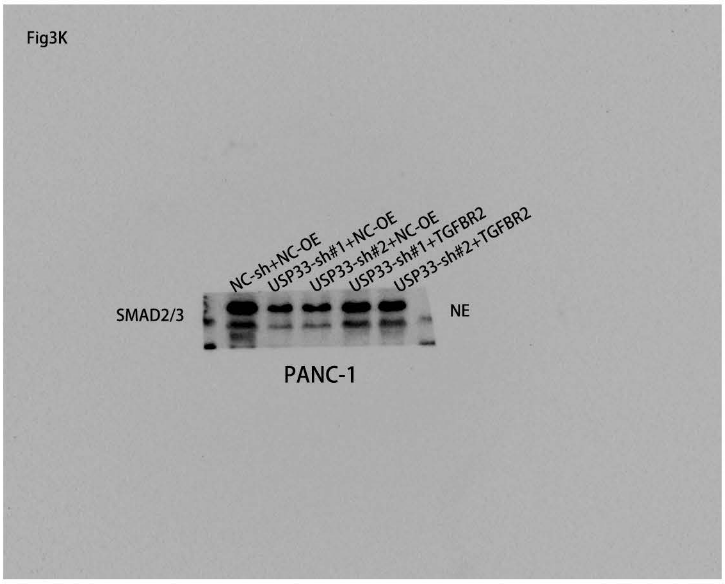
Fig3K
NC-sh+NC-OE
USP33-sh#1+NC-OE
USP33-sh#2+NC-OE
USP33-sh#1+TGFBR2
USP33-sh#2+TGFBR2
SMAD2/3
NE
PANC-1

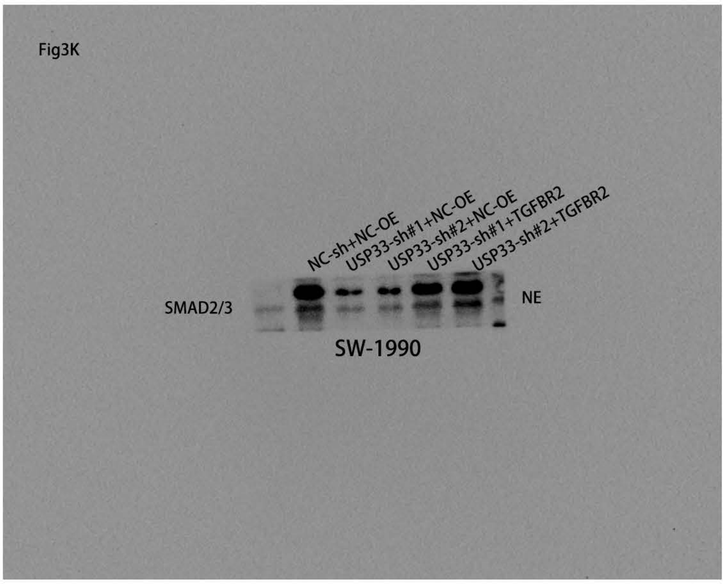
Fig3K
NC-sh+NC-OE
USP33-sh#1+NC-OE
USP33-sh#2+NC-OE
USP33-sh#1+TGFBR2
USP33-sh#2+TGFBR2
SMAD2/3
NE
SW-1990

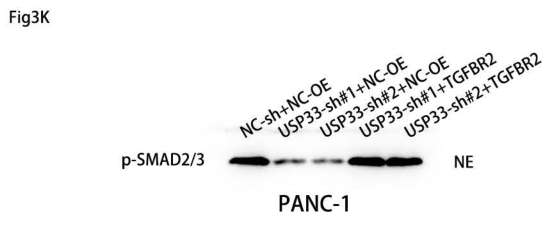
Fig3K
NC-sh+NC-OE
USP33-sh#1+NC-OE
USP33-sh#2+NC-OE
USP33-sh#1+TGFBR2
USP33-sh#2+TGFBR2
p-SMAD2/3
NE
PANC-1

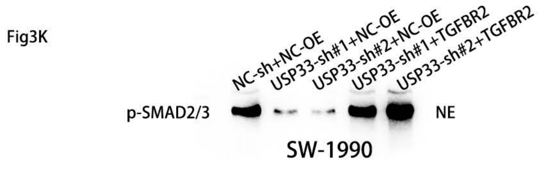
Fig3K
NC-sh+NC-OE
USP33-sh#1+NC-OE
USP33-sh#2+NC-OE
USP33-sh#1+TGFBR2
USP33-sh#2+TGFBR2
p-SMAD2/3
NE
SW-1990

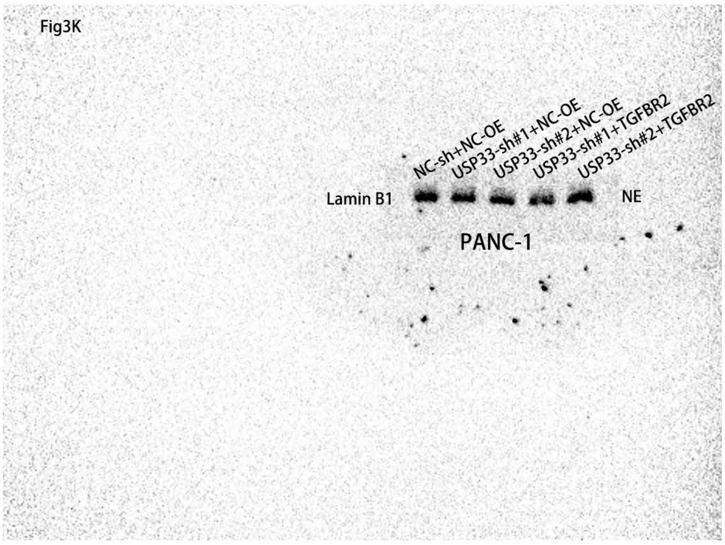
Fig3K
NC-sh+NC-OE
USP33-sh#1+NC-OE
USP33-sh#2+NC-OE
USP33-sh#1+TGFBR2
USP33-sh#2+TGFBR2
Lamin B1
NE
PANC-1

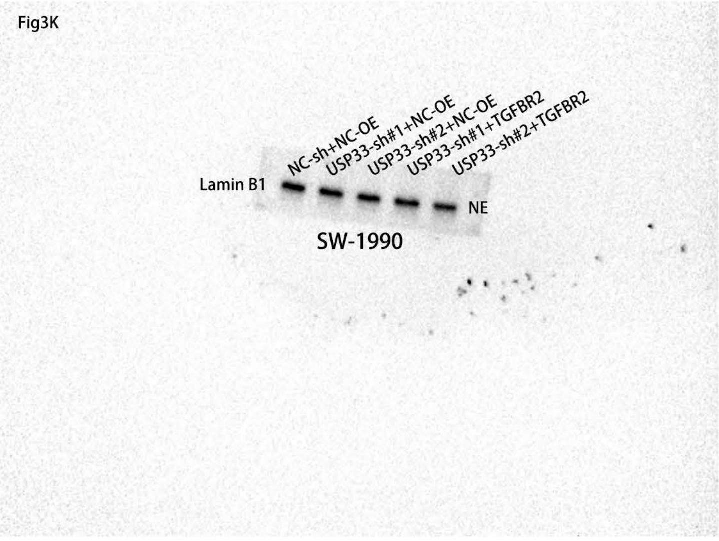
Fig3K
NC-sh+NC-OE
USP33-sh#1+NC-OE
USP33-sh#2+NC-OE
USP33-sh#1+TGFBR2
USP33-sh#2+TGFBR2
Lamin B1
NE
SW-1990

Fig3L

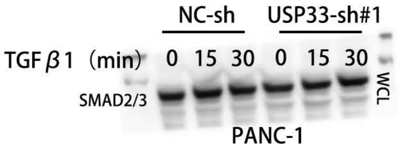
NC-sh
USP33-sh#1
TGFβ1 (min) 0 15 30 0 15 30
SMAD2/3
WCL
PANC-1

Fig3L

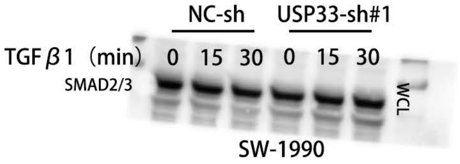
NC-sh
USP33-sh#1
TGFβ1 (min) 0 15 30 0 15 30
SMAD2/3
WCL
SW-1990

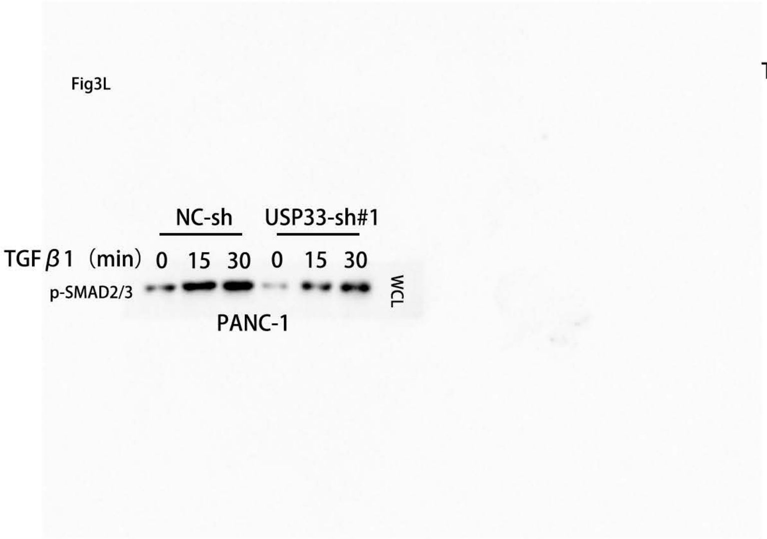
Fig3L
NC-sh
USP33-sh#1
TGFβ1 (min) 0 15 30 0 15 30
p-SMAD2/3
WCL
PANC-1

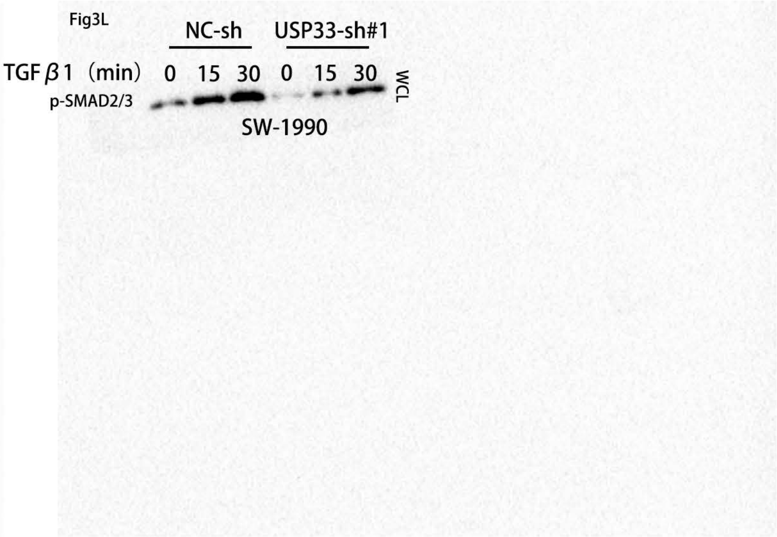
Fig3L
NC-sh
USP33-sh#1
TGFβ1 (min) 0 15 30 0 15 30
p-SMAD2/3
WCL
SW-1990

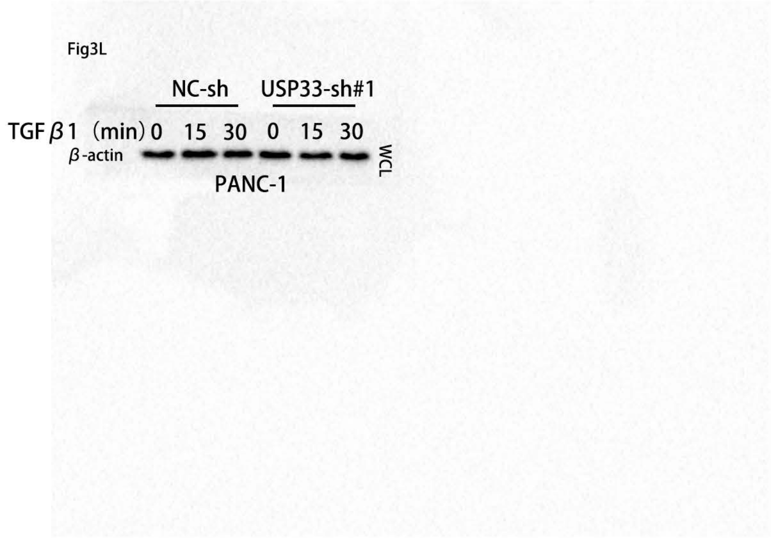
Fig3L
NC-sh
USP33-sh#1
TGFβ1 (min) 0 15 30 0 15 30
β-actin
WCL
PANC-1

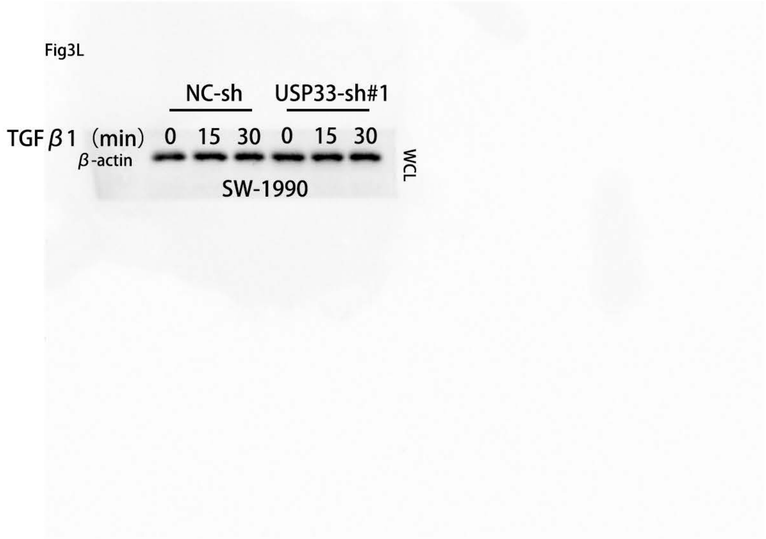
Fig3L
NC-sh
USP33-sh#1
TGFβ1 (min) 0 15 30 0 15 30
β-actin
WCL
SW-1990

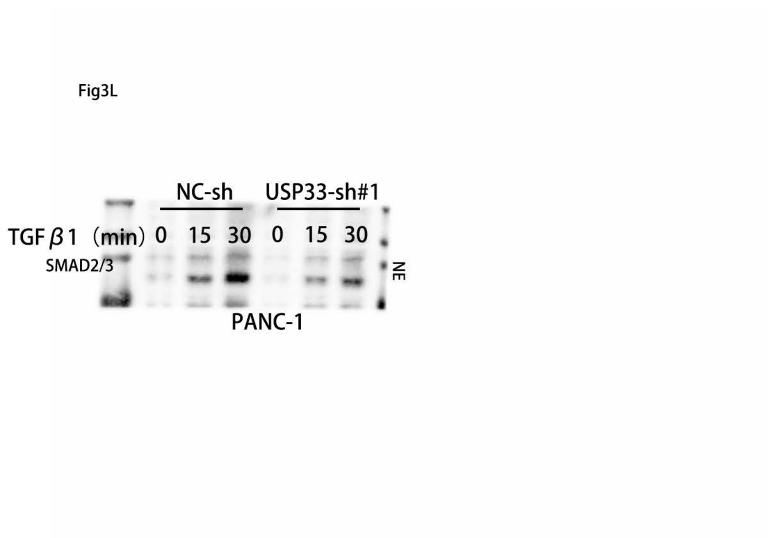
Fig3L
NC-sh
USP33-sh#1
TGFβ1 (min) 0 15 30 0 15 30
SMAD2/3
NE
PANC-1

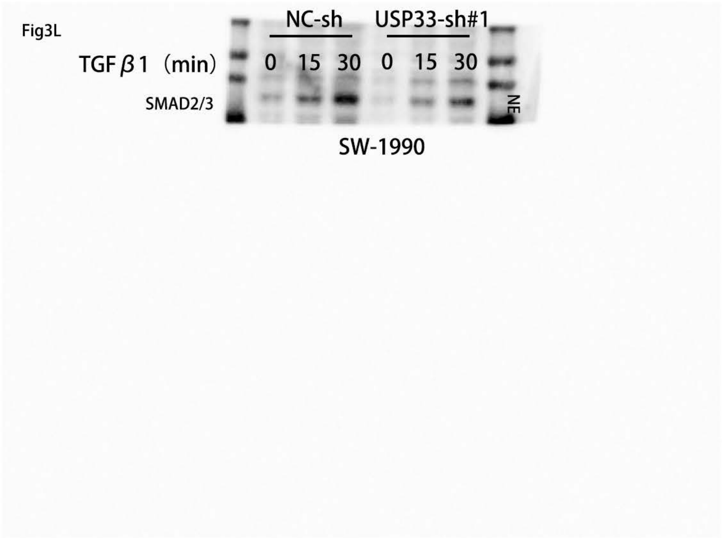
Fig3L
NC-sh
USP33-sh#1
TGFβ1 (min) 0 15 30 0 15 30
SMAD2/3
NE
SW-1990

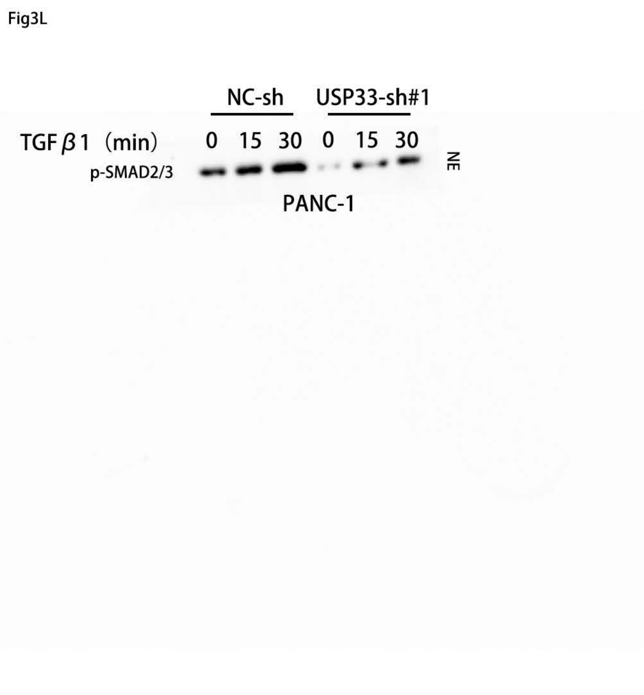
Fig3L
NC-sh
USP33-sh#1
TGFβ1 (min) 0 15 30 0 15 30
p-SMAD2/3
NE
PANC-1

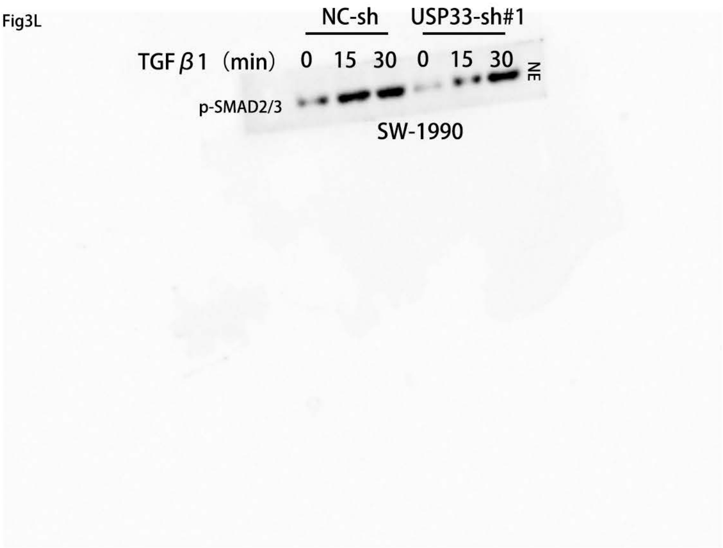
Fig3L
NC-sh
USP33-sh#1
TGFβ1 (min) 0 15 30 0 15 30
p-SMAD2/3
NE
SW-1990

Fig3L

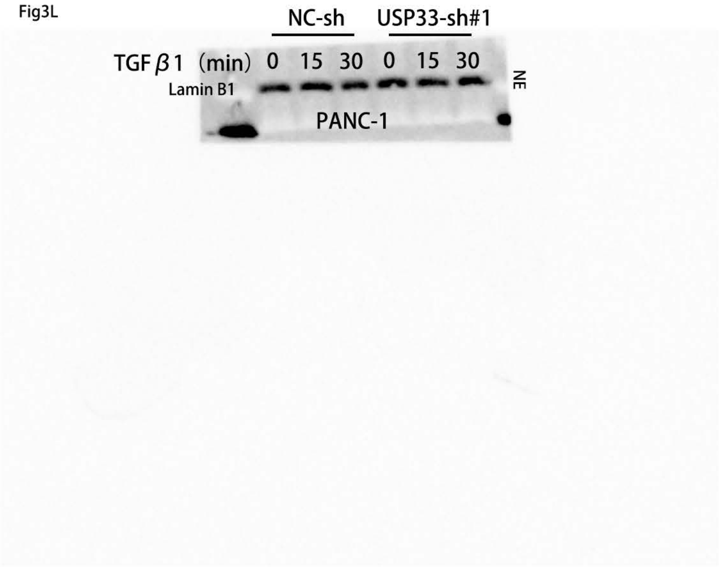

Fig3L

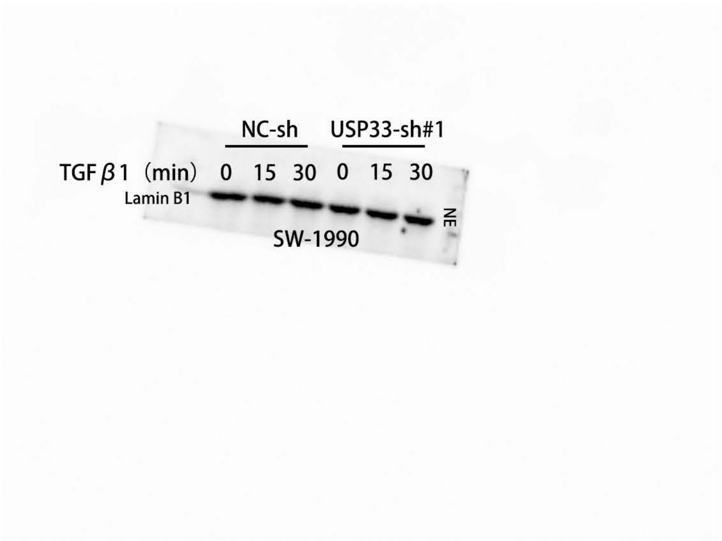

Fig4J

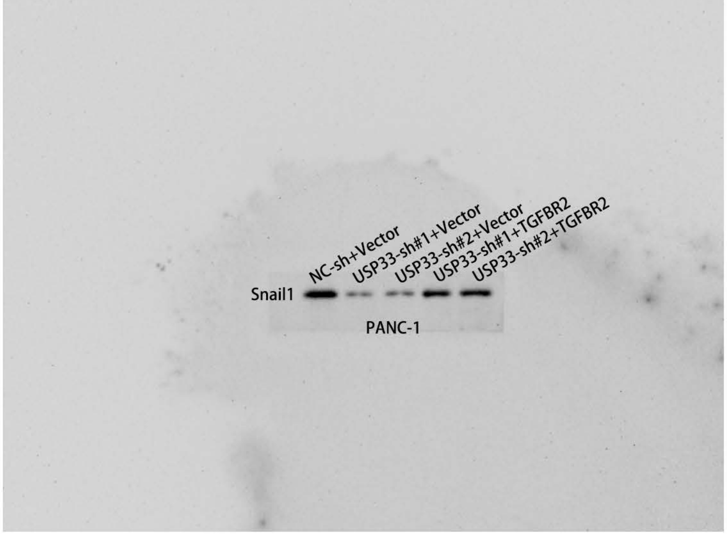

Fig4J

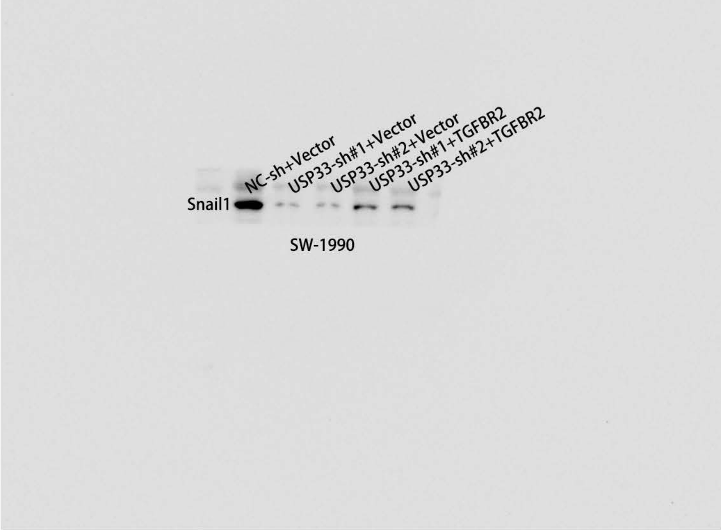

Fig4J

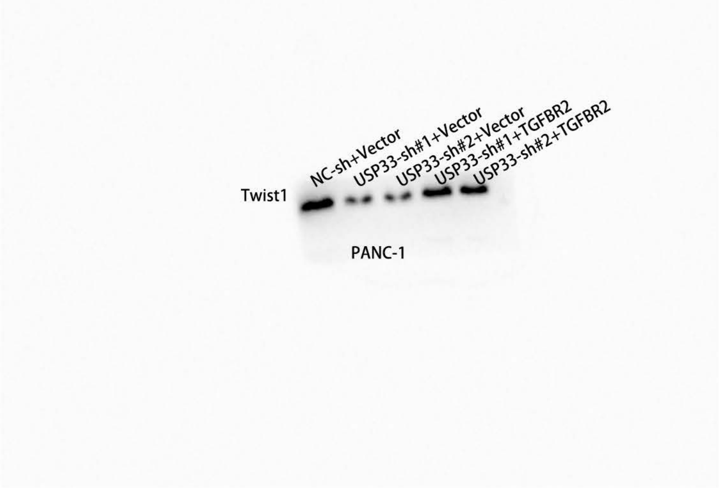

Fig4J

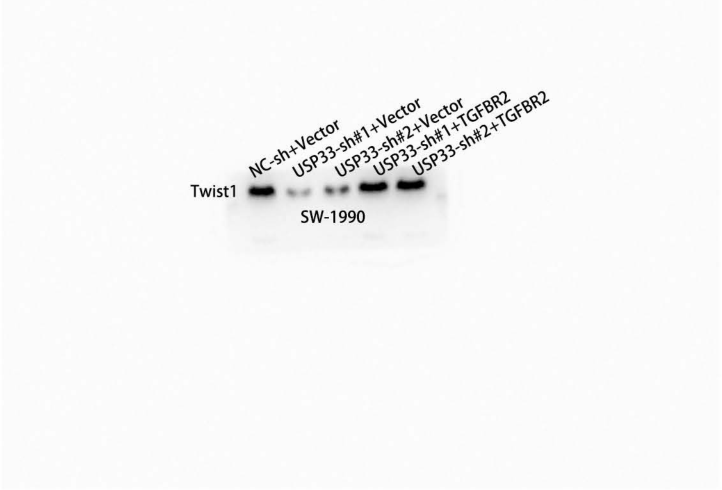

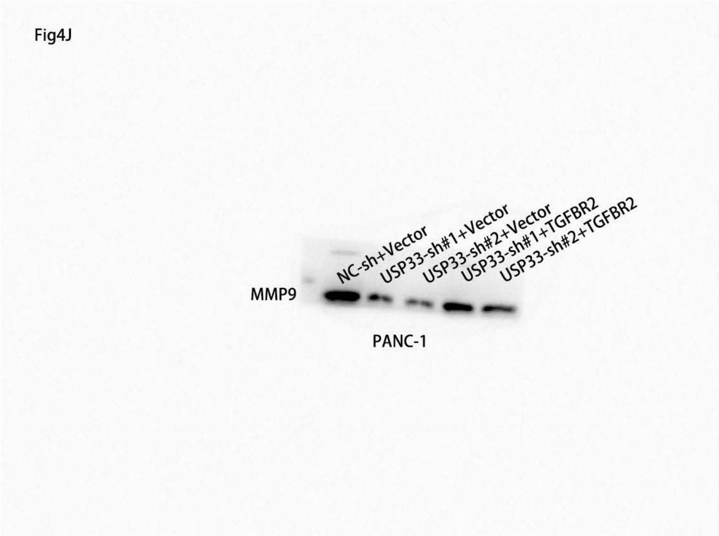
Fig4J
NC-sh+Vector
USP33-sh#1+Vector
USP33-sh#2+Vector
USP33-sh#1+TGFBR2
USP33-sh#2+TGFBR2
MMP9
PANC-1

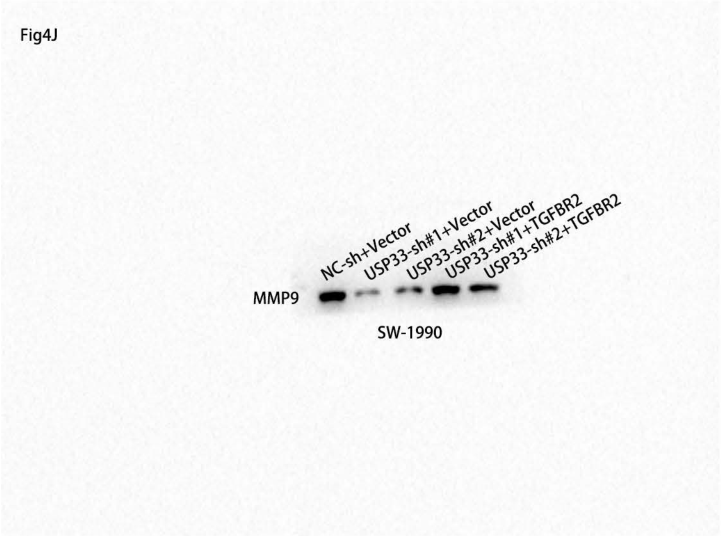
Fig4J
NC-sh+Vector
USP33-sh#1+Vector
USP33-sh#2+Vector
USP33-sh#1+TGFBR2
USP33-sh#2+TGFBR2
MMP9
SW-1990

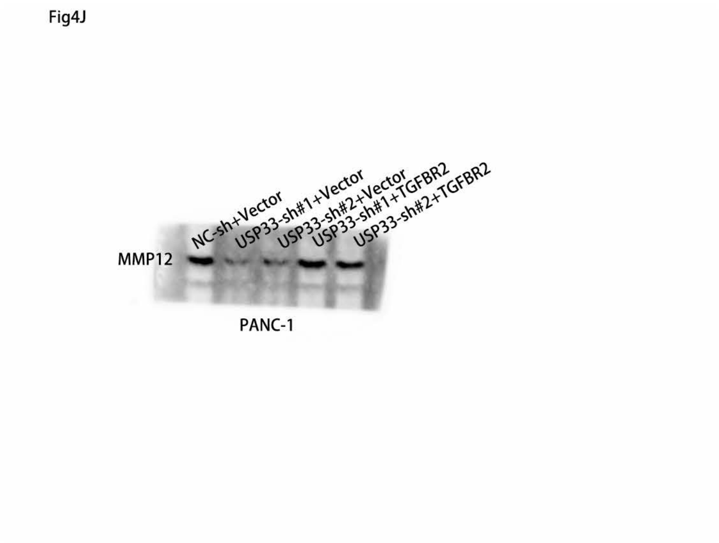
Fig4J
NC-sh+Vector
USP33-sh#1+Vector
USP33-sh#2+Vector
USP33-sh#1+TGFBR2
USP33-sh#2+TGFBR2
MMP12
PANC-1

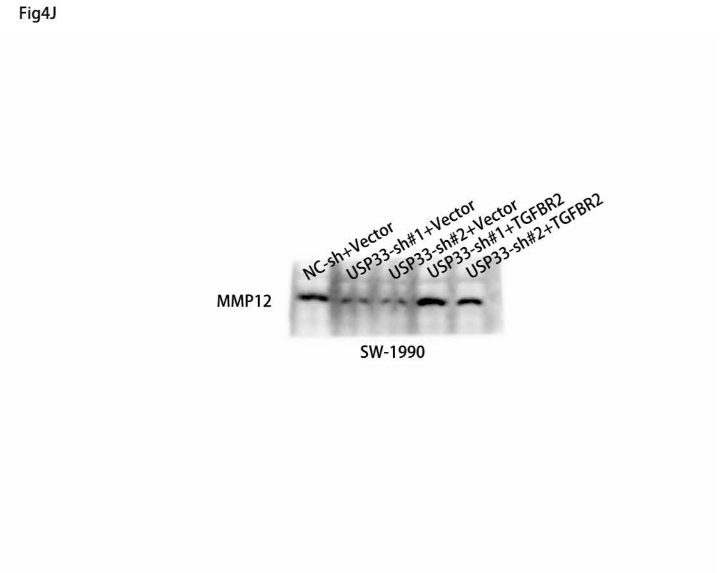
Fig4J
NC-sh+Vector
USP33-sh#1+Vector
USP33-sh#2+Vector
USP33-sh#1+TGFBR2
USP33-sh#2+TGFBR2
MMP12
SW-1990

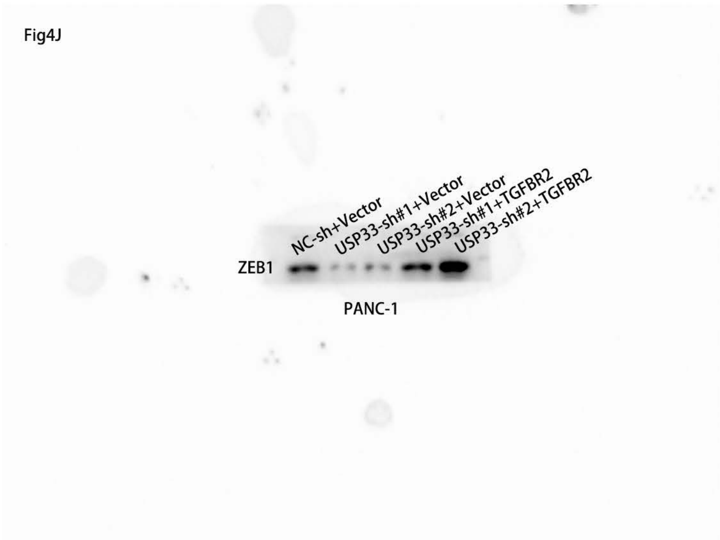
Fig4J
NC-sh+Vector
USP33-sh#1+Vector
USP33-sh#2+Vector
USP33-sh#1+TGFBR2
USP33-sh#2+TGFBR2
ZEB1
PANC-1

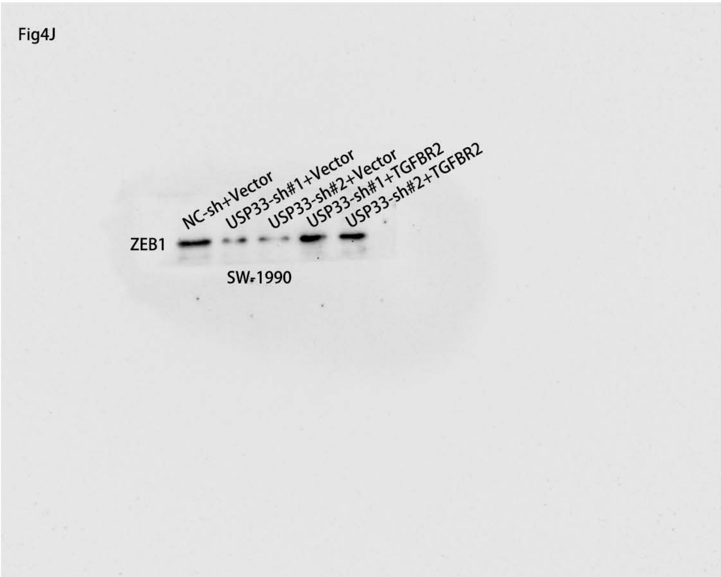
Fig4J
NC-sh+Vector
USP33-sh#1+Vector
USP33-sh#2+Vector
USP33-sh#1+TGFBR2
USP33-sh#2+TGFBR2
ZEB1
SW-1990

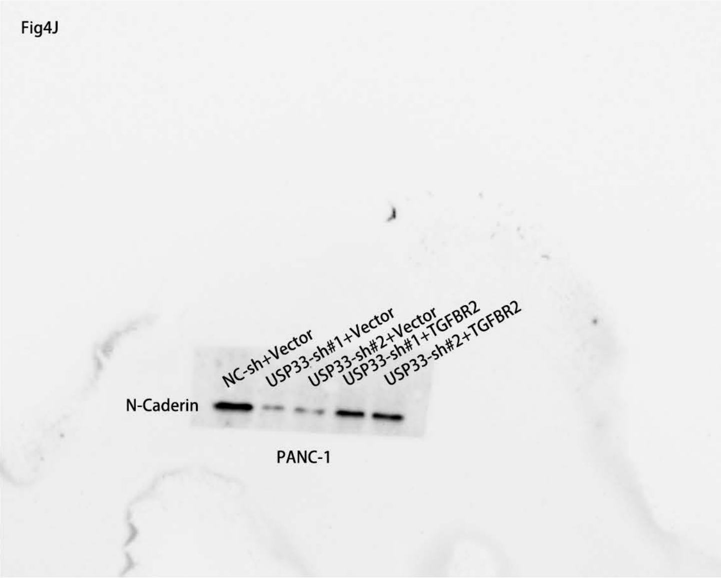
Fig4J
NC-sh+Vector
USP33-sh#1+Vector
USP33-sh#2+Vector
USP33-sh#1+TGFBR2
USP33-sh#2+TGFBR2
N-Caderin
PANC-1

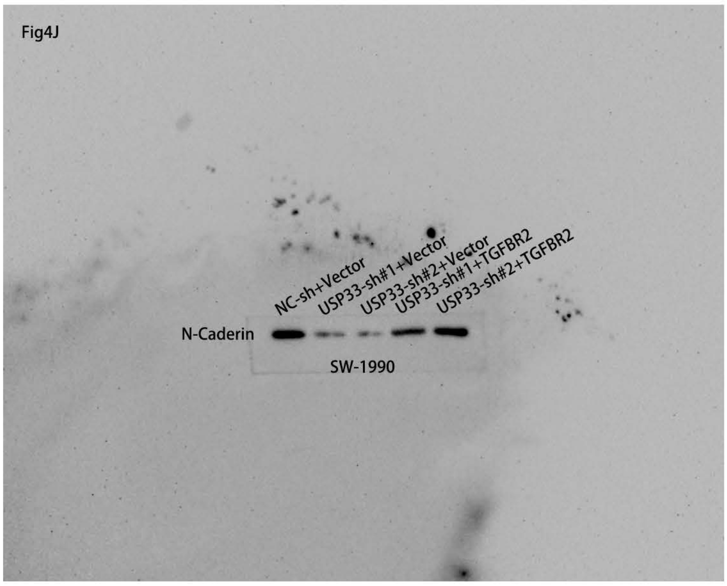
Fig4J
NC-sh+Vector
USP33-sh#1+Vector
USP33-sh#2+Vector
USP33-sh#1+TGFBR2
USP33-sh#2+TGFBR2
N-Caderin
SW-1990

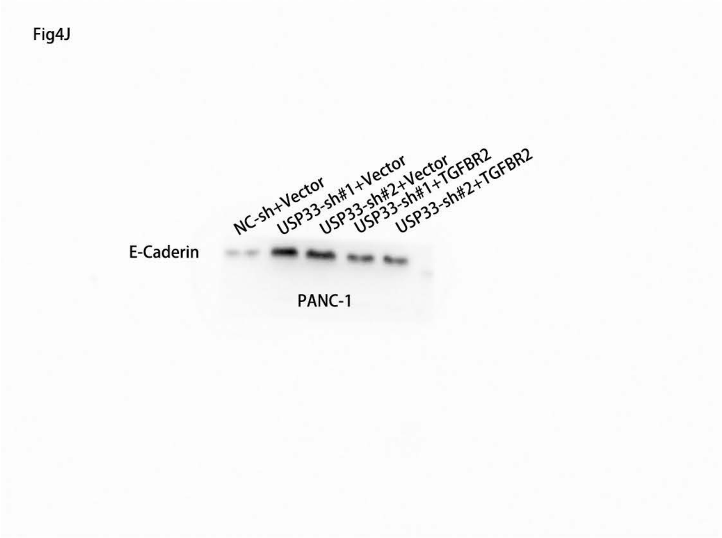
Fig4J
NC-sh+Vector
USP33-sh#1+Vector
USP33-sh#2+Vector
USP33-sh#1+TGFBR2
USP33-sh#2+TGFBR2
E-Caderin
PANC-1

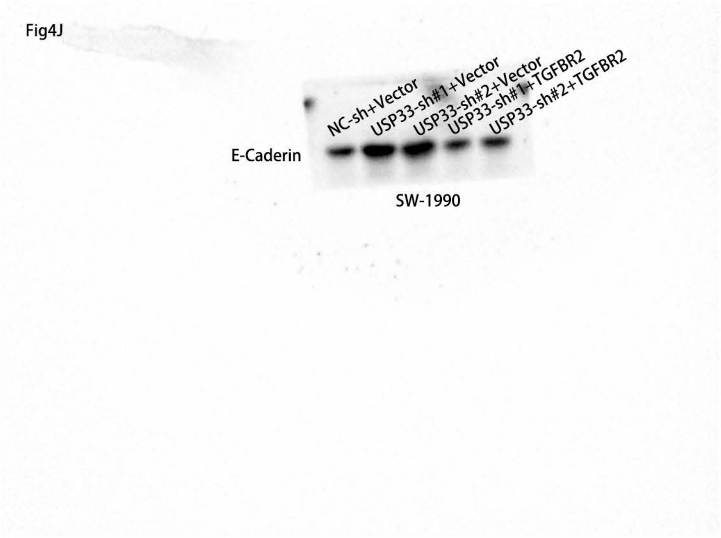
Fig4J
NC-sh+Vector
USP33-sh#1+Vector
USP33-sh#2+Vector
USP33-sh#1+TGFBR2
USP33-sh#2+TGFBR2
E-Caderin
SW-1990

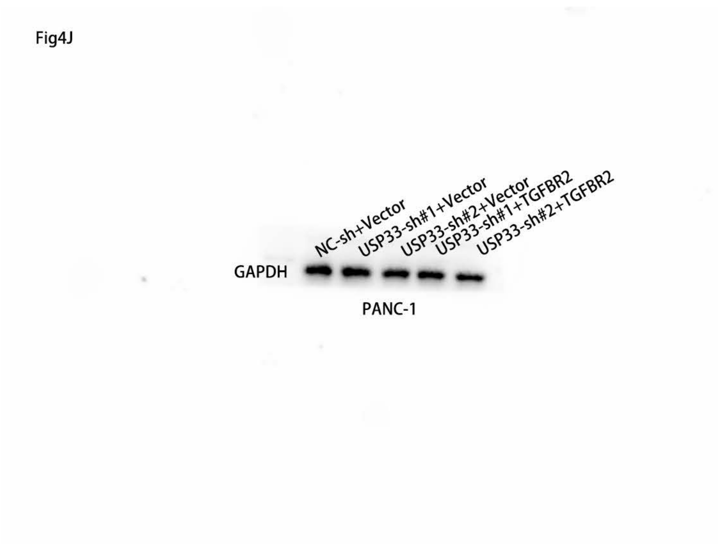
Fig4J
NC-sh+Vector
USP33-sh#1+Vector
USP33-sh#2+Vector
USP33-sh#1+TGFBR2
USP33-sh#2+TGFBR2
GAPDH
PANC-1

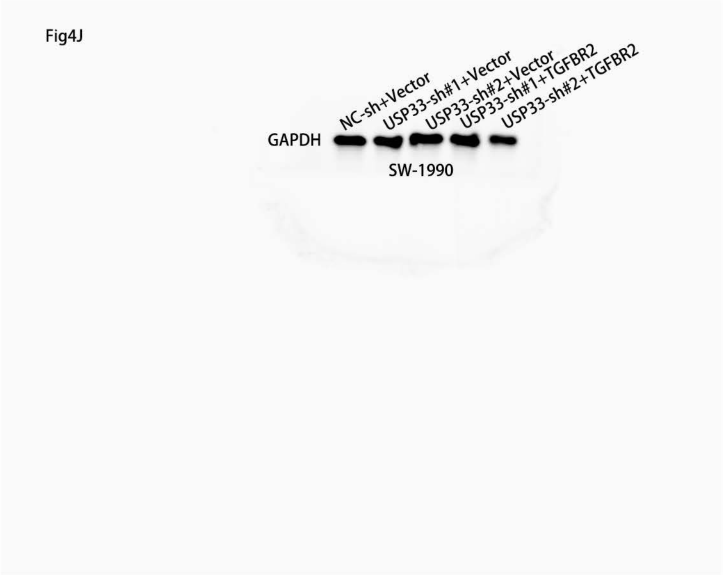
Fig4J
NC-sh+Vector
USP33-sh#1+Vector
USP33-sh#2+Vector
USP33-sh#1+TGFBR2
USP33-sh#2+TGFBR2
GAPDH
SW-1990

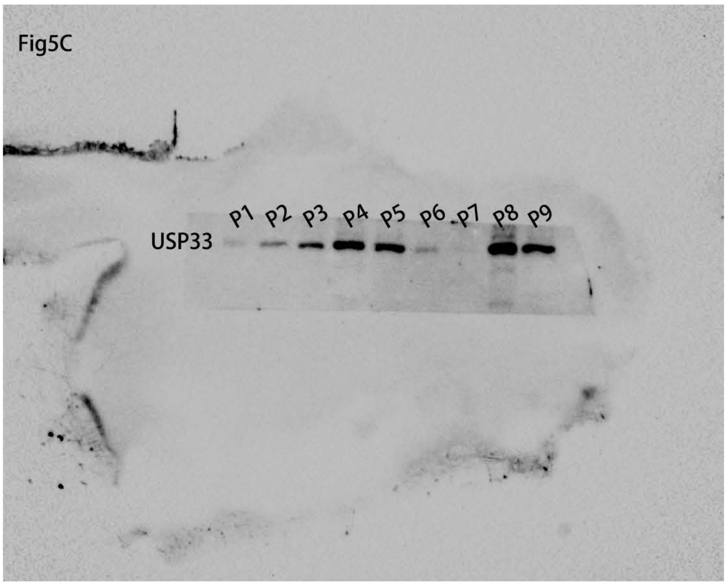
Fig5C
USP33
P1 P2 P3 P4 P5 P6 P7 P8 P9

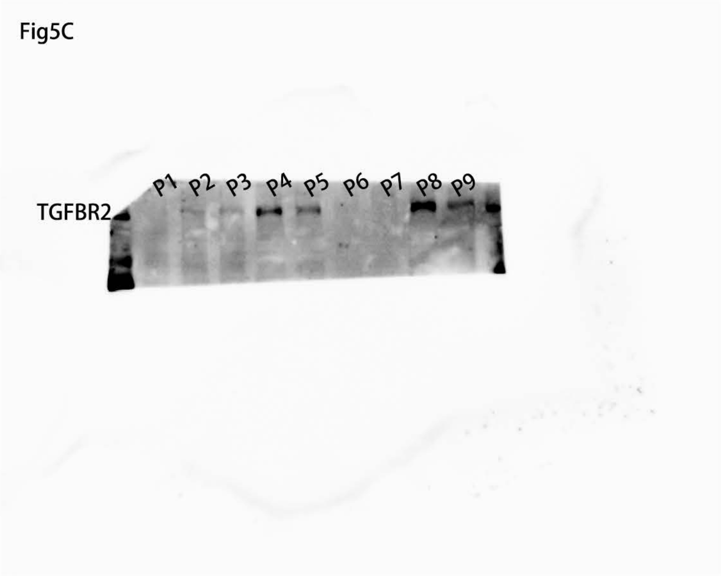
Fig5C
TGFBR2
P1 P2 P3 P4 P5 P6 P7 P8 P9

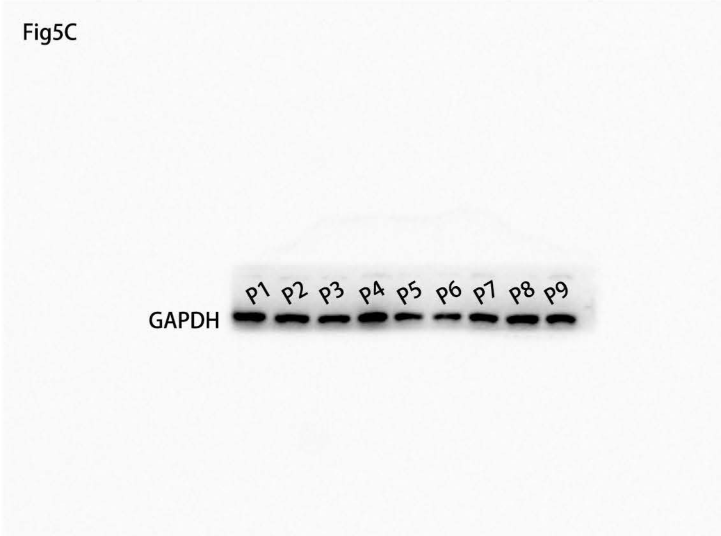
Fig5C
GAPDH
P1 P2 P3 P4 P5 P6 P7 P8 P9

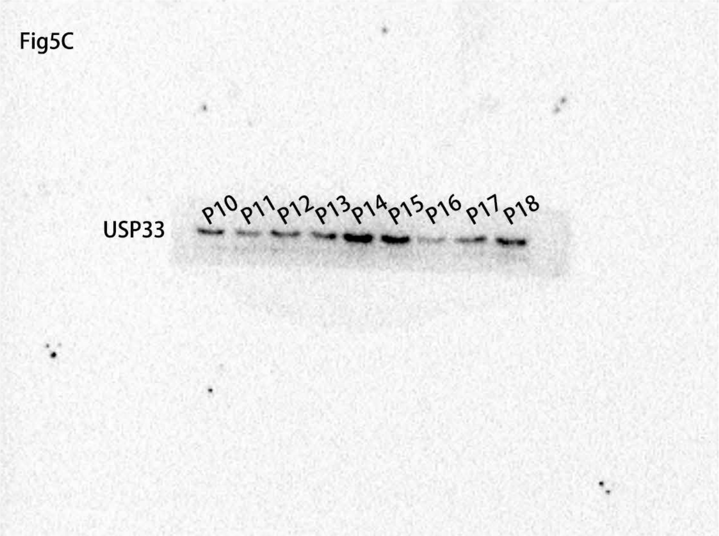
Fig5C
USP33
P10 P11 P12 P13 P14 P15 P16 P17 P18

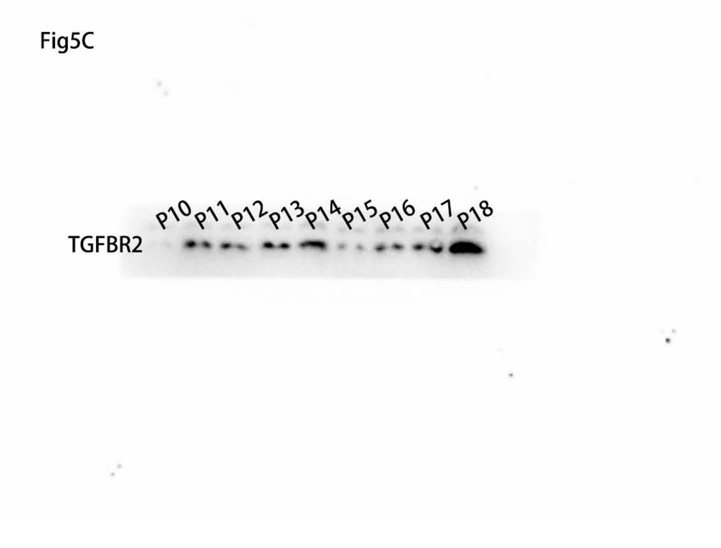
Fig5C
TGFBR2
P10 P11 P12 P13 P14 P15 P16 P17 P18

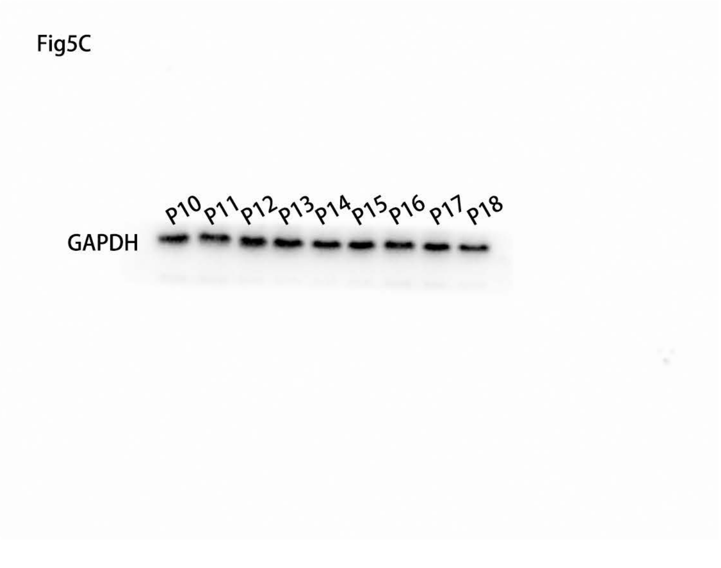
Fig5C
GAPDH
P10 P11 P12 P13 P14 P15 P16 P17 P18

Fig5F

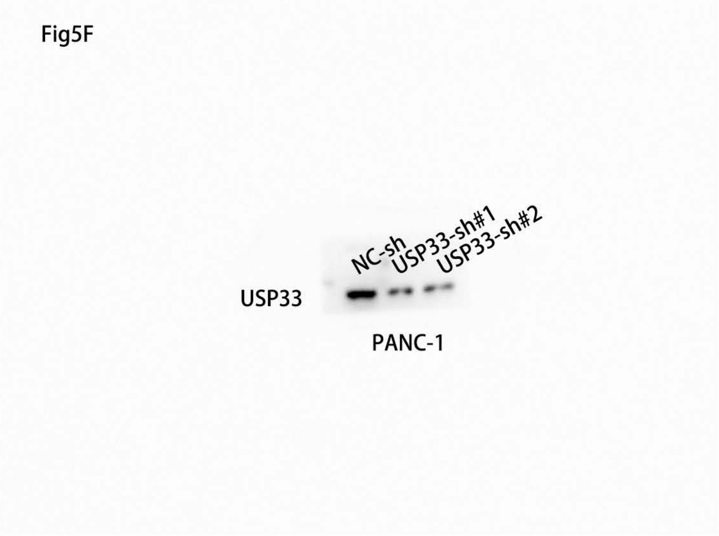

Fig5F

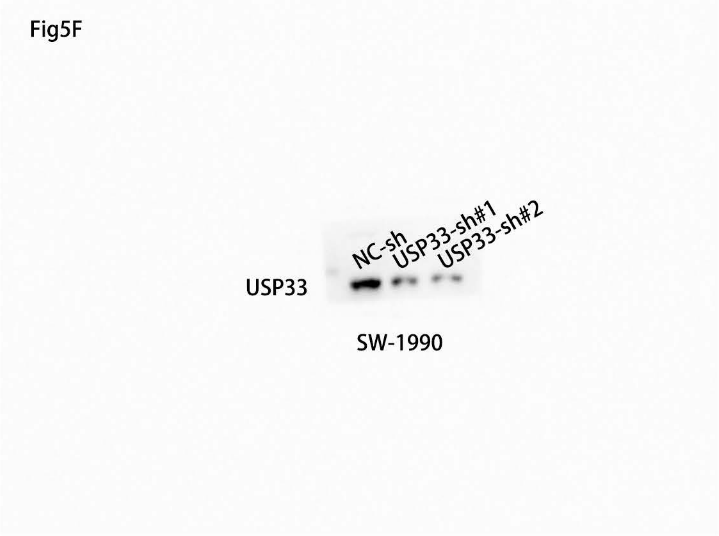

Fig5F

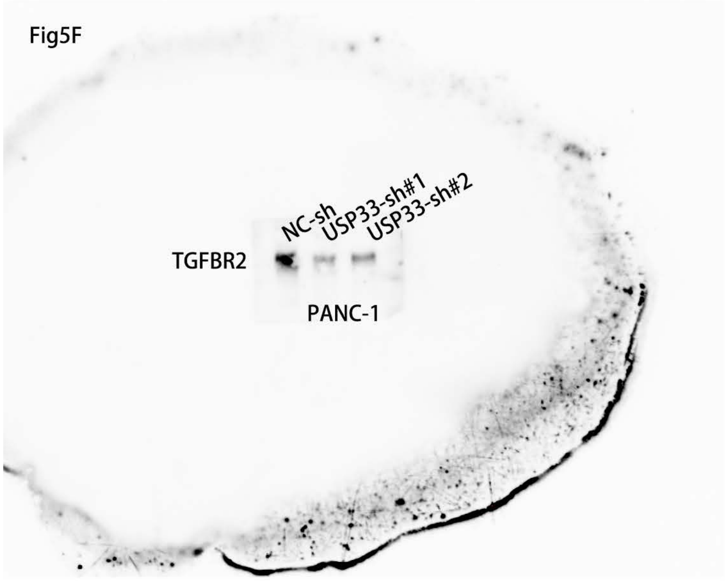

Fig5F

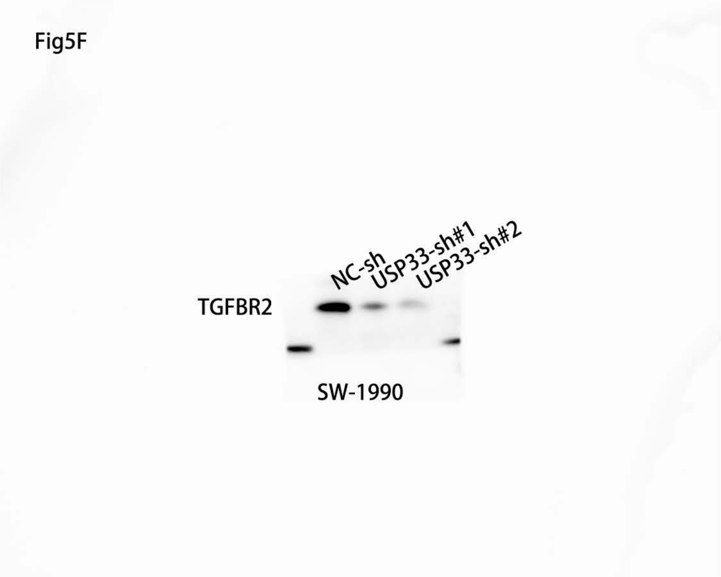

Fig5F

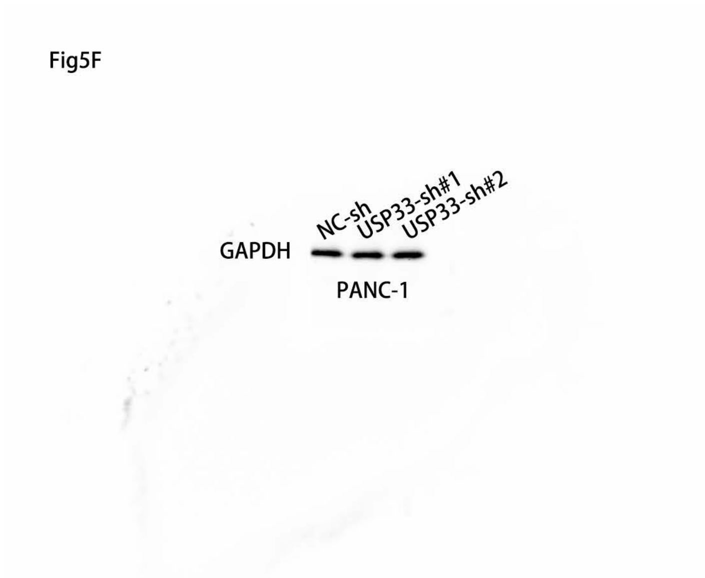

Fig5F

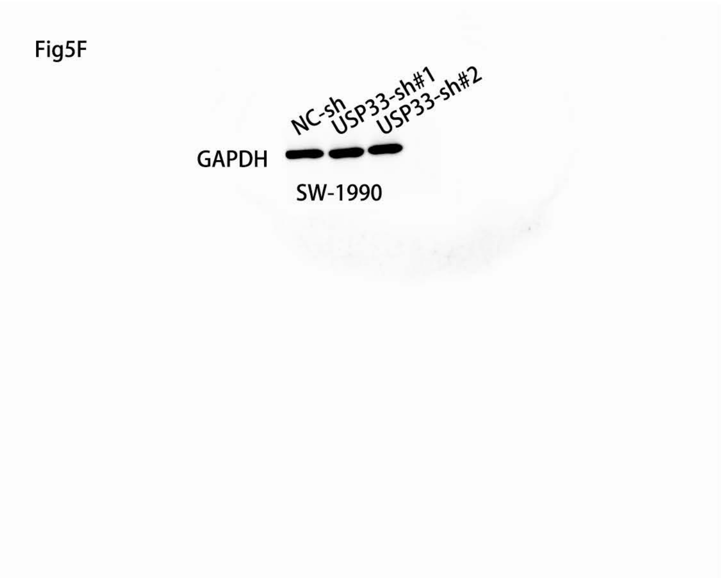

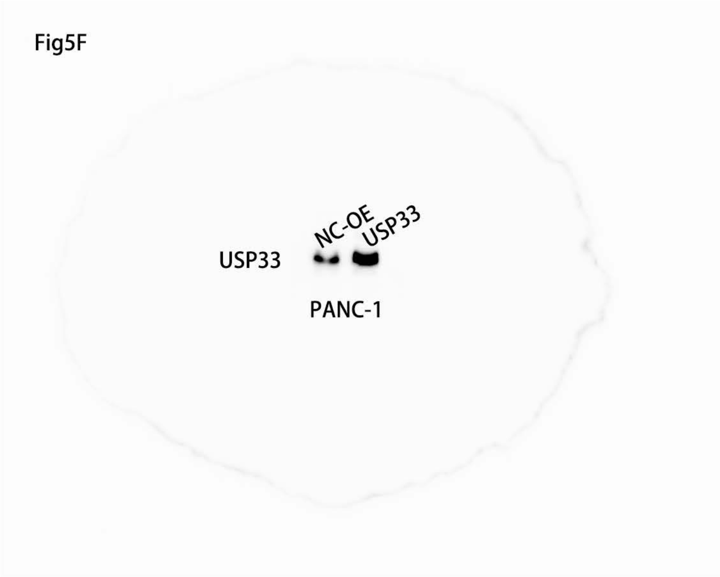
Fig5F
NC-OE
USP33
USP33
PANC-1

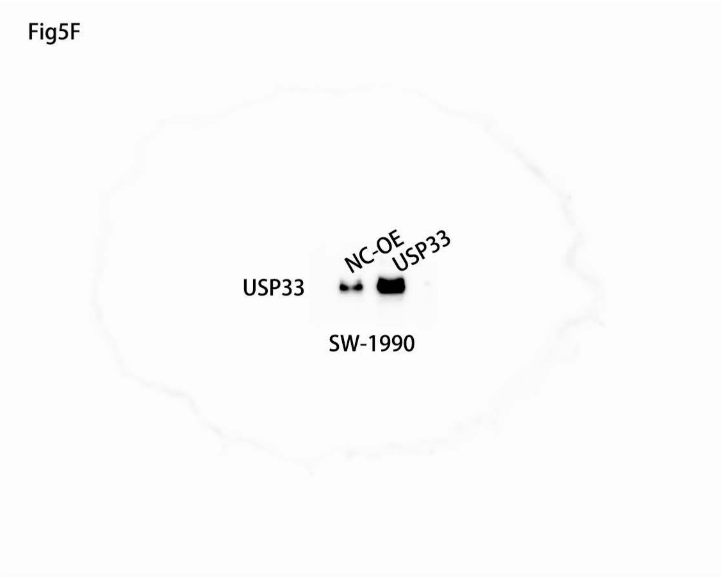
Fig5F
NC-OE
USP33
USP33
SW-1990

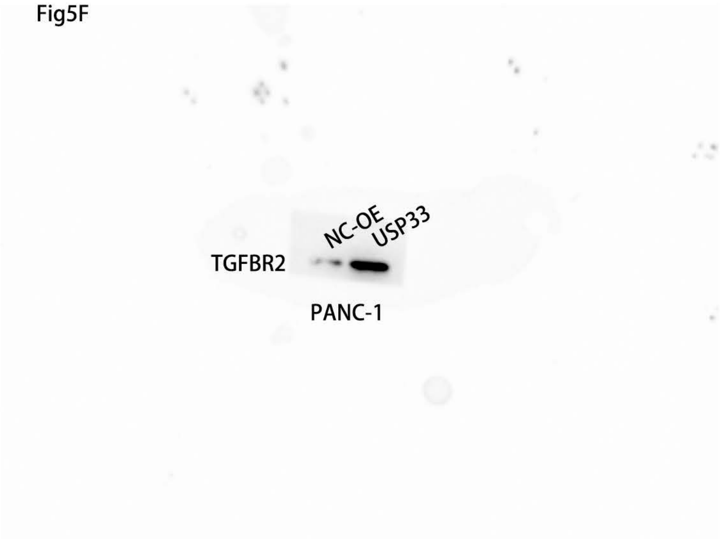
Fig5F
NC-OE
USP33
TGFBR2
PANC-1

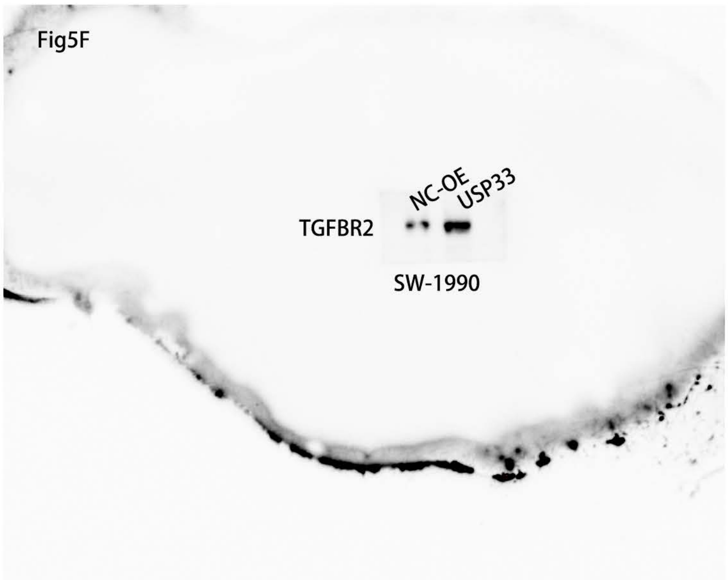
Fig5F
NC-OE
USP33
TGFBR2
SW-1990

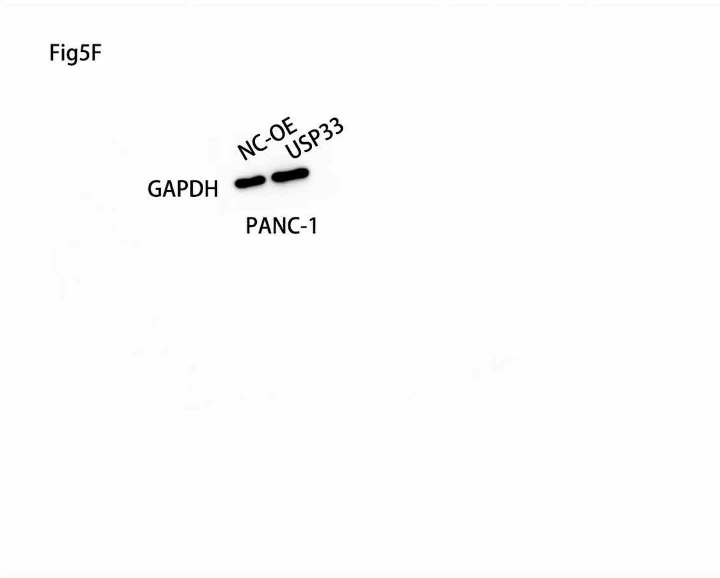
Fig5F
NC-OE
USP33
GAPDH
PANC-1

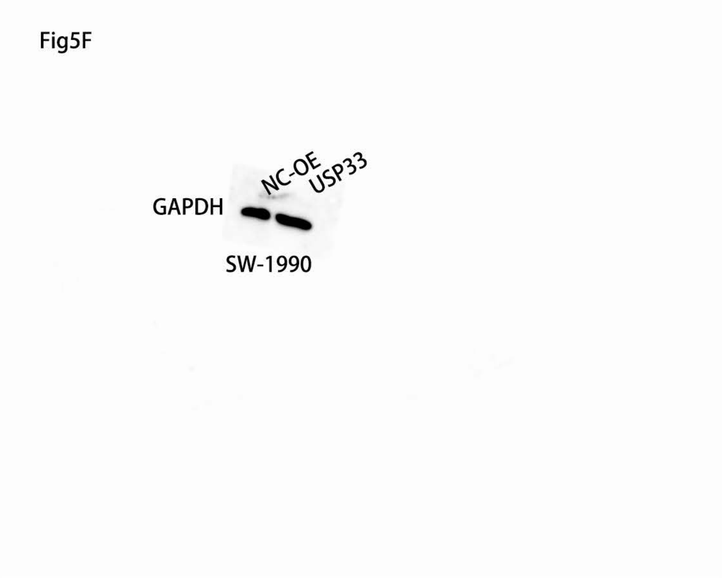
Fig5F
NC-OE
USP33
GAPDH
SW-1990

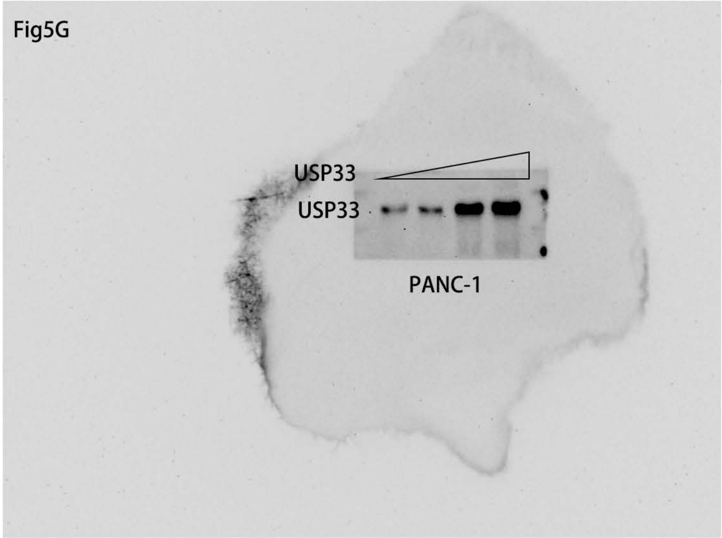
Fig5G
USP33
USP33
PANC-1

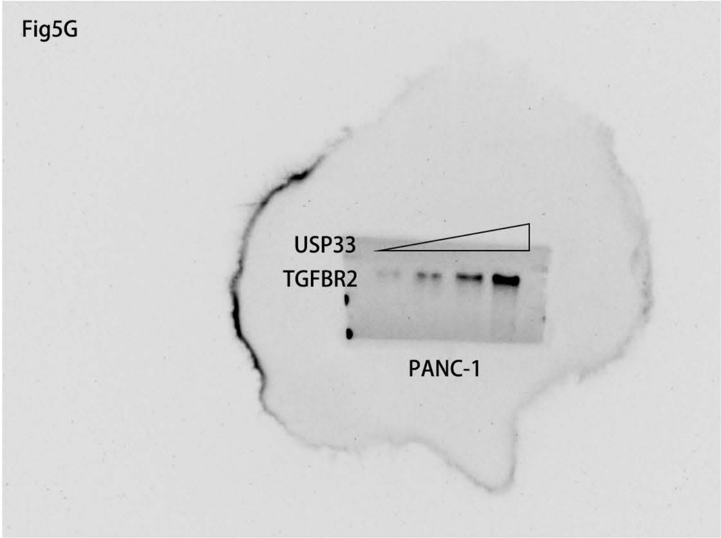
Fig5G
USP33
TGFBR2
PANC-1

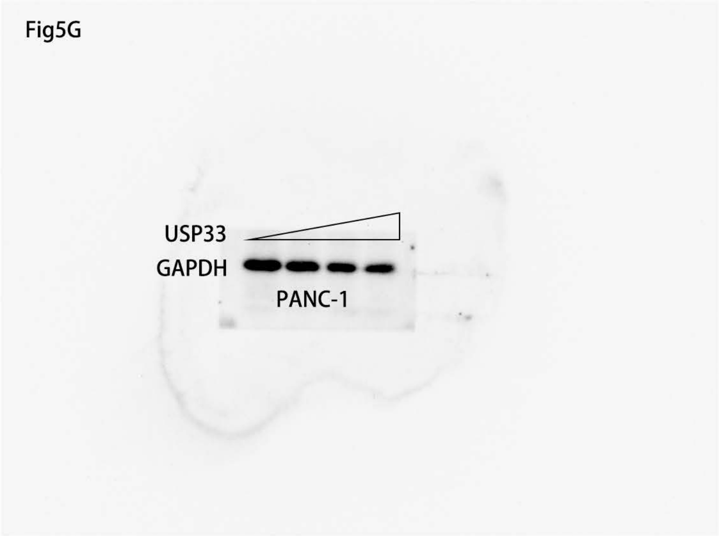
Fig5G
USP33
GAPDH
PANC-1

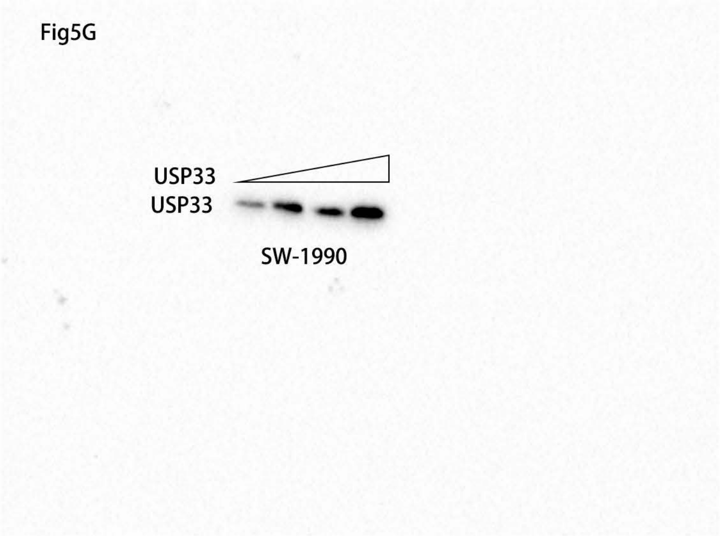
Fig5G
USP33
USP33
SW-1990

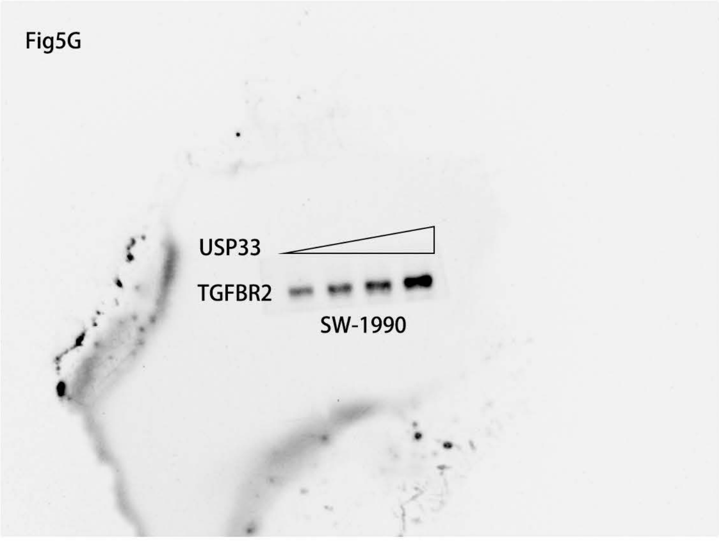
Fig5G
USP33
TGFBR2
SW-1990

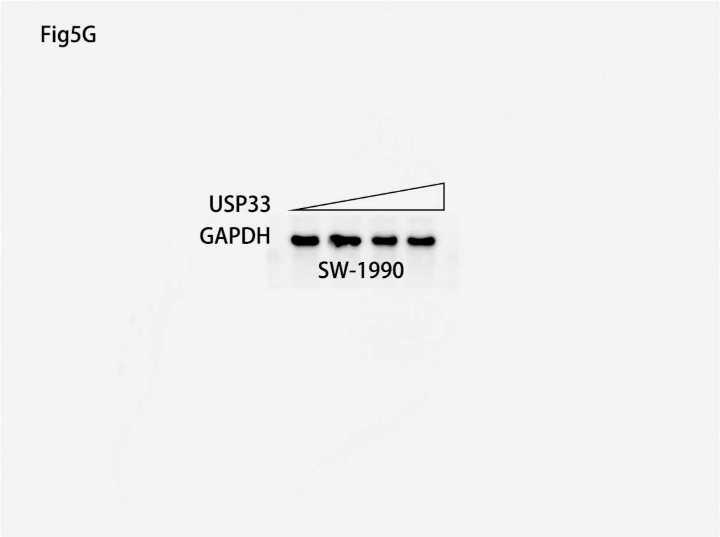
Fig5G
USP33
GAPDH
SW-1990

Fig5H

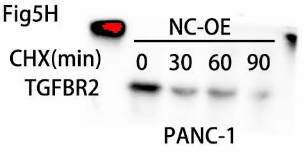

Fig5H

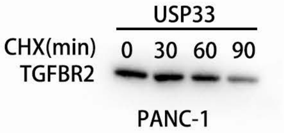

Fig5H

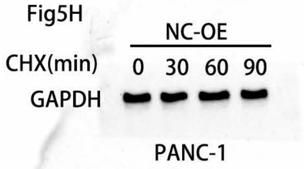

Fig5H

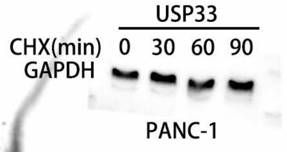

Fig5H

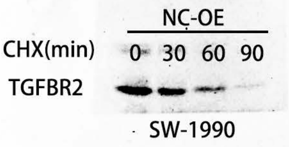

Fig5H

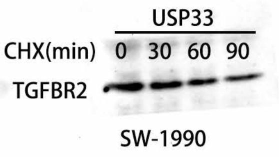

Fig5H

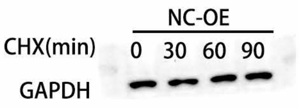

Fig5H

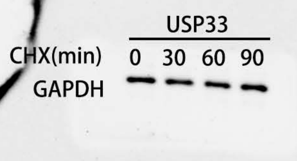

Fig5J

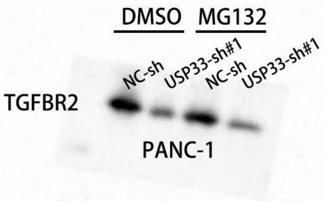

Fig5J

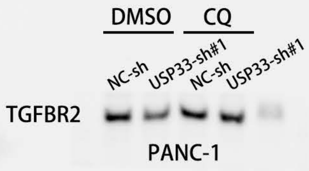

Fig5J

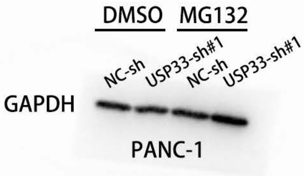

Fig5J

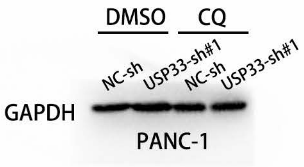

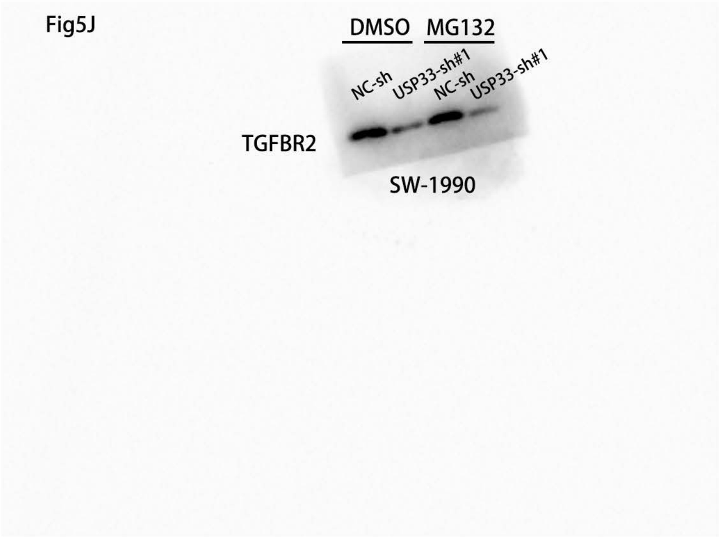
Fig5J
DMSO
MG132
NC-sh
USP33-sh#1
NC-sh
USP33-sh#1
TGFBR2
SW-1990

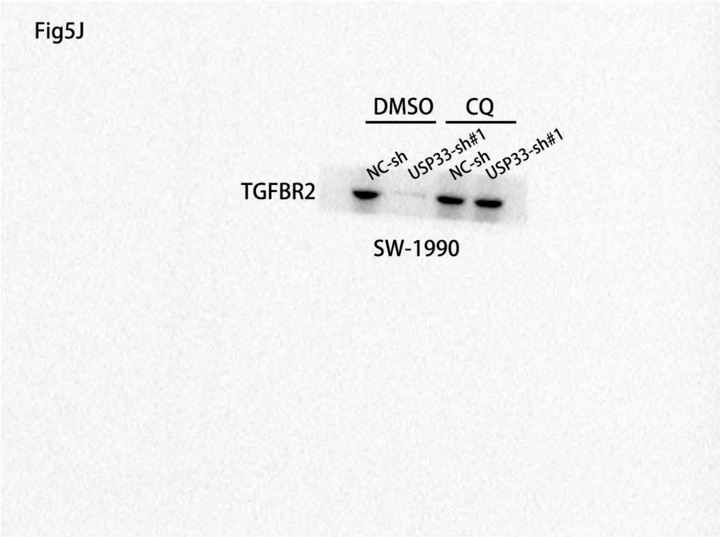
Fig5J
DMSO
CQ
NC-sh
USP33-sh#1
NC-sh
USP33-sh#1
TGFBR2
SW-1990

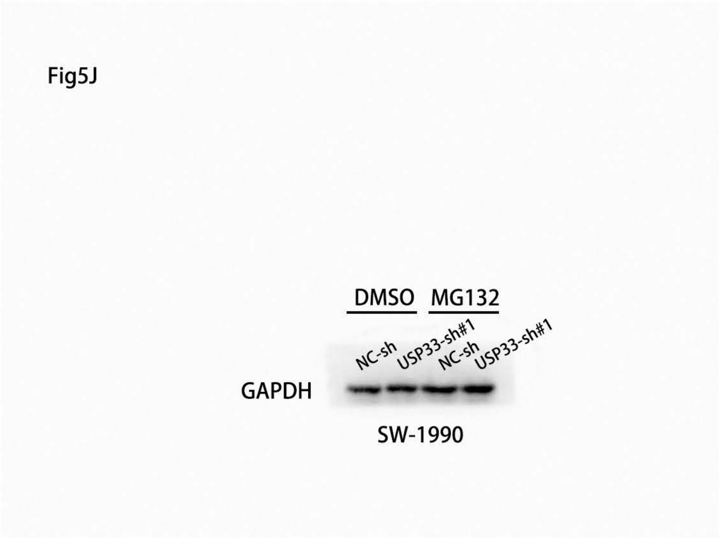
Fig5J
DMSO
MG132
NC-sh
USP33-sh#1
NC-sh
USP33-sh#1
GAPDH
SW-1990

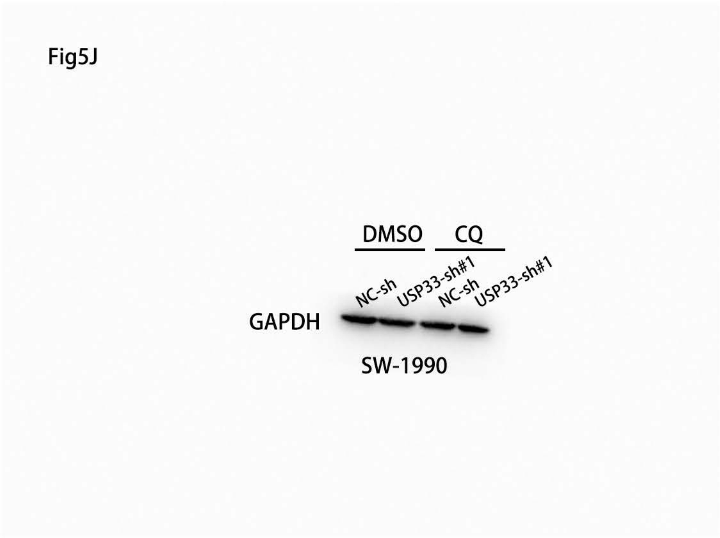
Fig5J
DMSO
CQ
NC-sh
USP33-sh#1
NC-sh
USP33-sh#1
GAPDH
SW-1990

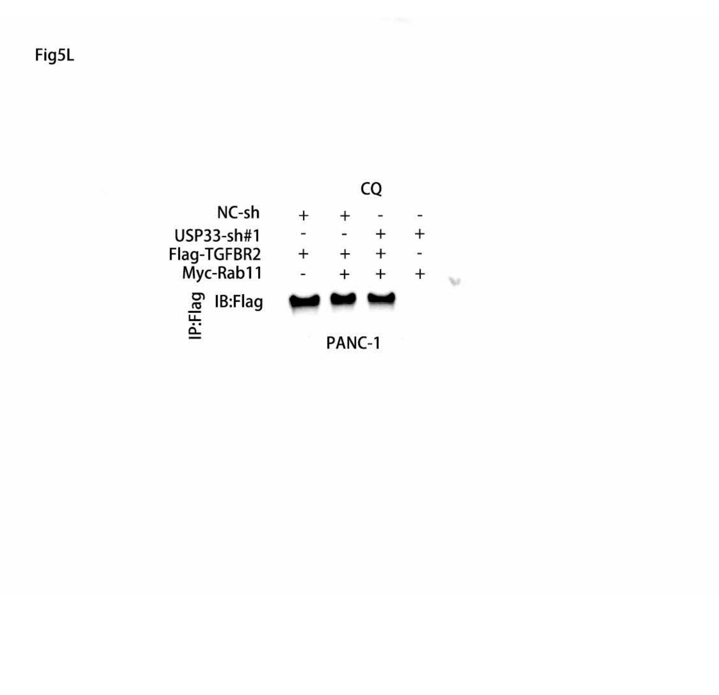
Fig5L
CQ
NC-sh + + - -
USP33-sh#1 - - + +
Flag-TGFBR2 + + + -
Myc-Rab11 - + + +
IP:Flag
IB:Flag
PANC-1

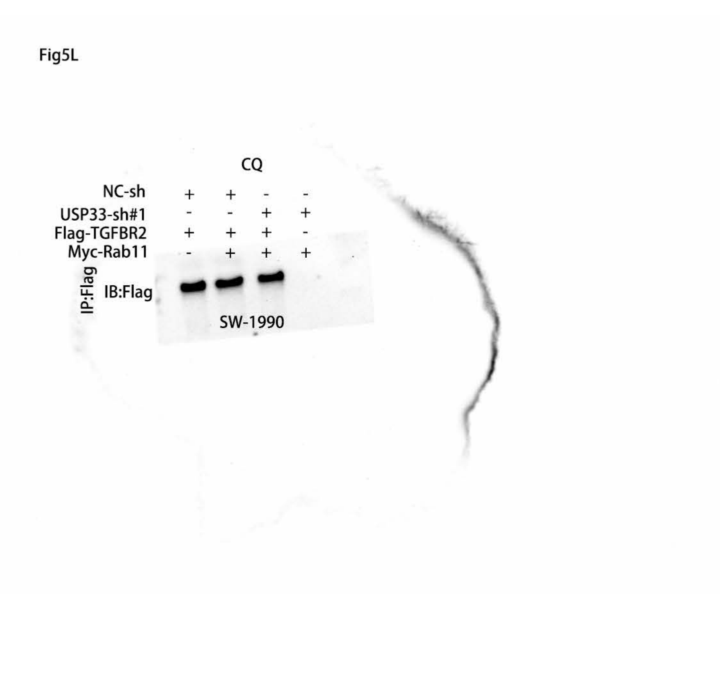
Fig5L
CQ
NC-sh + + - -
USP33-sh#1 - - + +
Flag-TGFBR2 + + + -
Myc-Rab11 - + + +
IP:Flag
IB:Flag
SW-1990

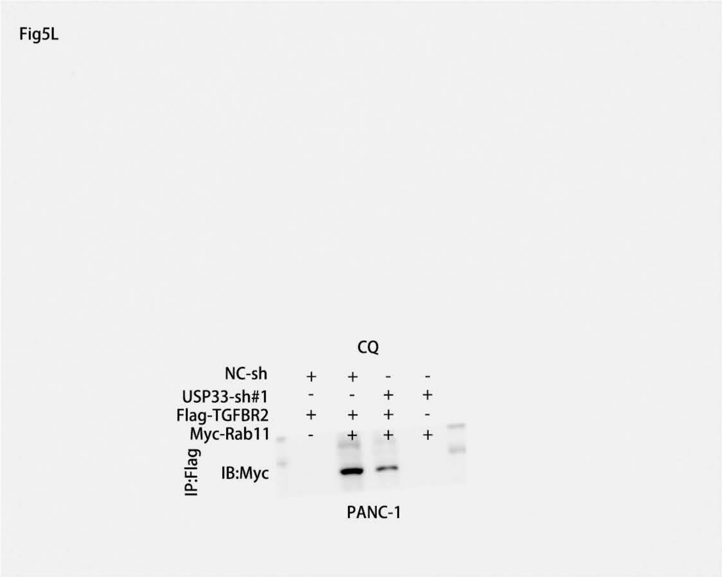
Fig5L
CQ
NC-sh + + - -
USP33-sh#1 - - + +
Flag-TGFBR2 + + + -
Myc-Rab11 - + + +
IP:Flag
IB:Myc
PANC-1

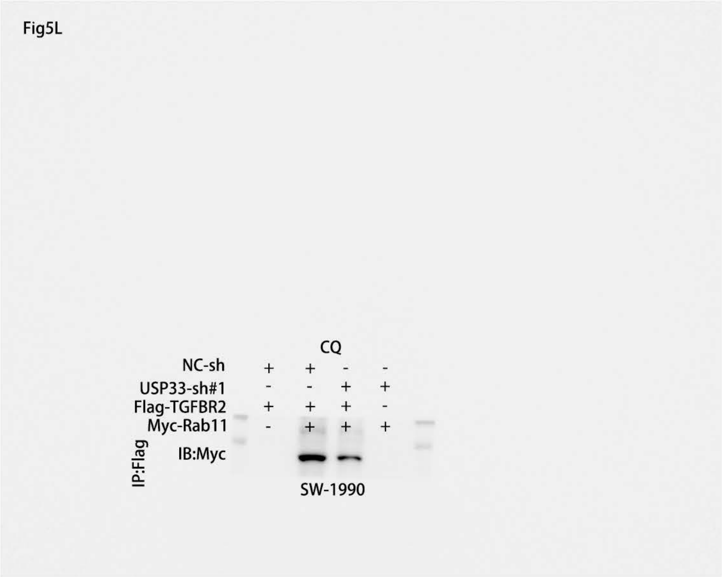
Fig5L
CQ
NC-sh + + - -
USP33-sh#1 - - + +
Flag-TGFBR2 + + + -
Myc-Rab11 - + + +
IP:Flag
IB:Myc
SW-1990

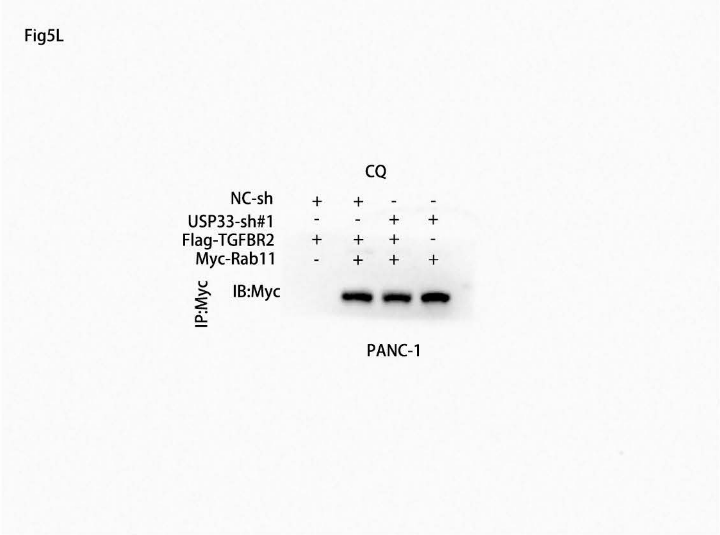
Fig5L
CQ
NC-sh + + - -
USP33-sh#1 - - + +
Flag-TGFBR2 + + + -
Myc-Rab11 - + + +
IP:Myc
IB:Myc
PANC-1

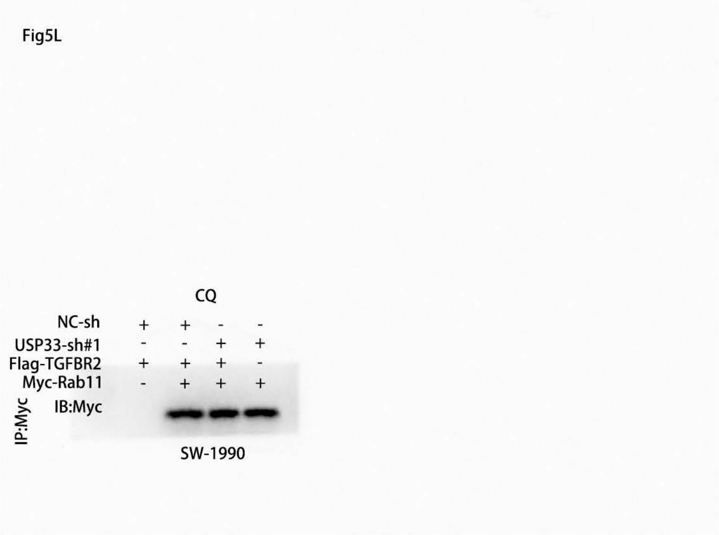
Fig5L
CQ
NC-sh + + - -
USP33-sh#1 - - + +
Flag-TGFBR2 + + + -
Myc-Rab11 - + + +
IP:Myc
IB:Myc
SW-1990

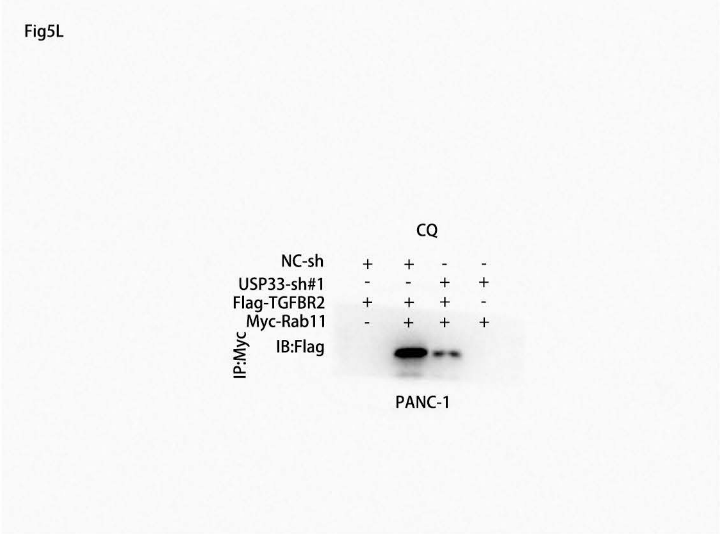
Fig5L
CQ
NC-sh + + - -
USP33-sh#1 - - + +
Flag-TGFBR2 + + + -
Myc-Rab11 - + + +
IP:Myc
IB:Flag
PANC-1

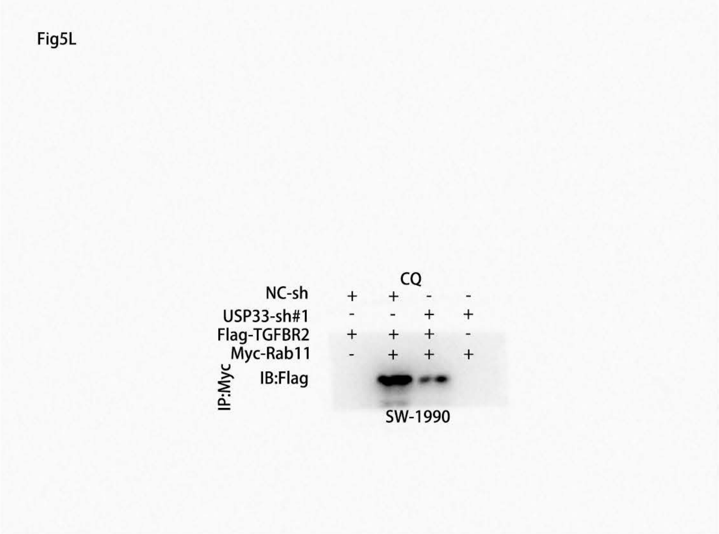
Fig5L
CQ
NC-sh + + - -
USP33-sh#1 - - + +
Flag-TGFBR2 + + + -
Myc-Rab11 - + + +
IP:Myc
IB:Flag
SW-1990

Fig5L

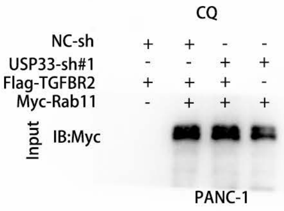

Fig5L

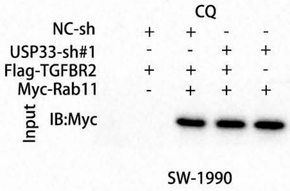

Fig5L

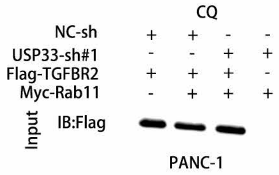

Fig5L

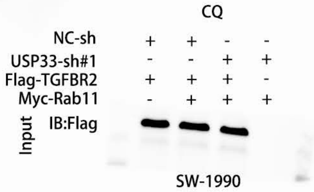

Fig5L

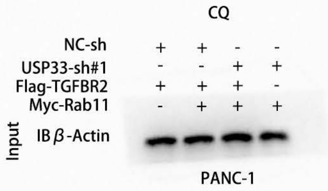

Fig5L

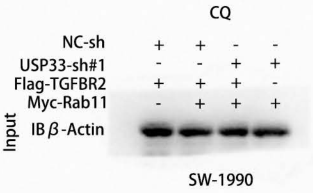

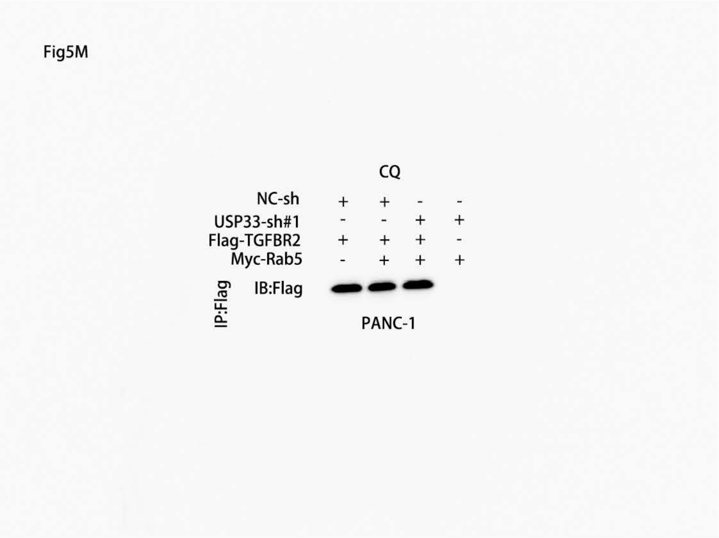
Fig5M
CQ
NC-sh + + - -
USP33-sh#1 - - + +
Flag-TGFBR2 + + + -
Myc-Rab5 - + + +
IP:Flag
IB:Flag
PANC-1

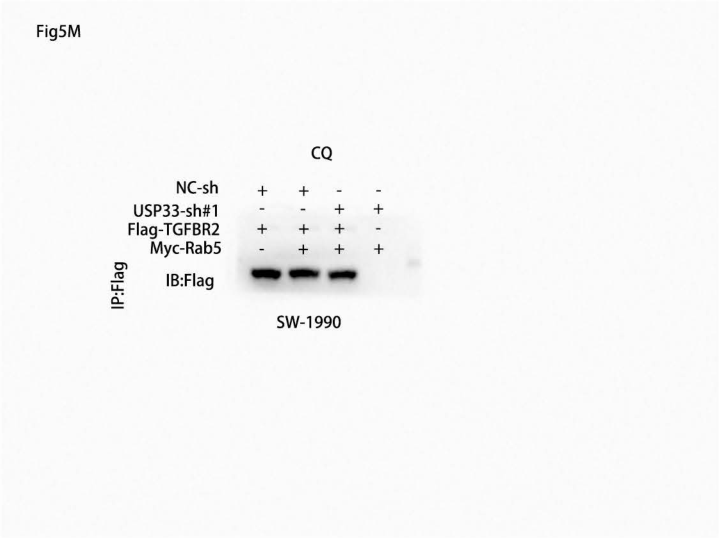
Fig5M
CQ
NC-sh + + - -
USP33-sh#1 - - + +
Flag-TGFBR2 + + + -
Myc-Rab5 - + + +
IP:Flag
IB:Flag
SW-1990

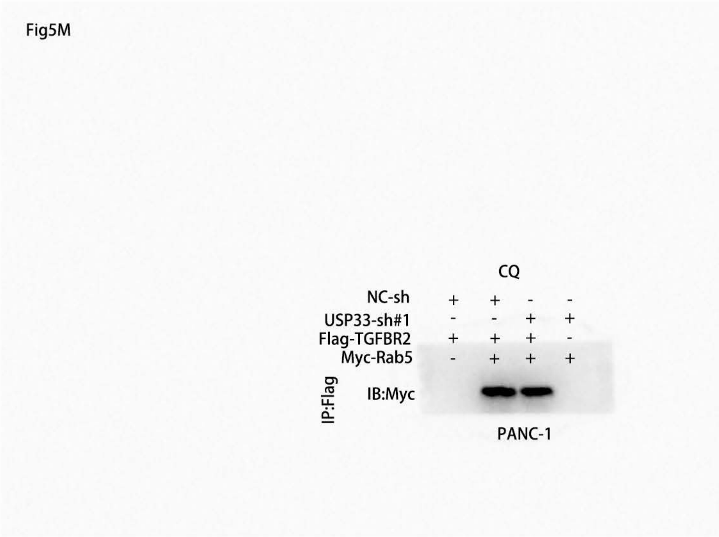
Fig5M
CQ
NC-sh + + - -
USP33-sh#1 - - + +
Flag-TGFBR2 + + + -
Myc-Rab5 - + + +
IP:Flag
IB:Myc
PANC-1

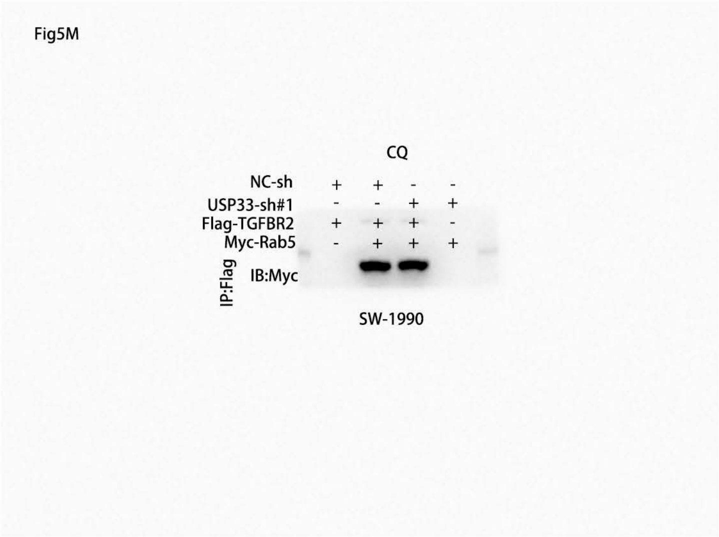
Fig5M
CQ
NC-sh + + - -
USP33-sh#1 - - + +
Flag-TGFBR2 + + + -
Myc-Rab5 - + + +
IP:Flag
IB:Myc
SW-1990

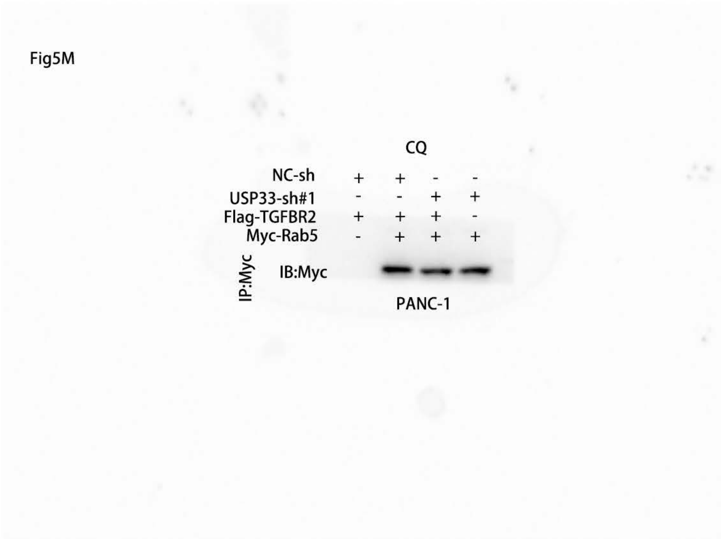
Fig5M
CQ
NC-sh + + - -
USP33-sh#1 - - + +
Flag-TGFBR2 + + + -
Myc-Rab5 - + + +
IP:Myc
IB:Myc
PANC-1

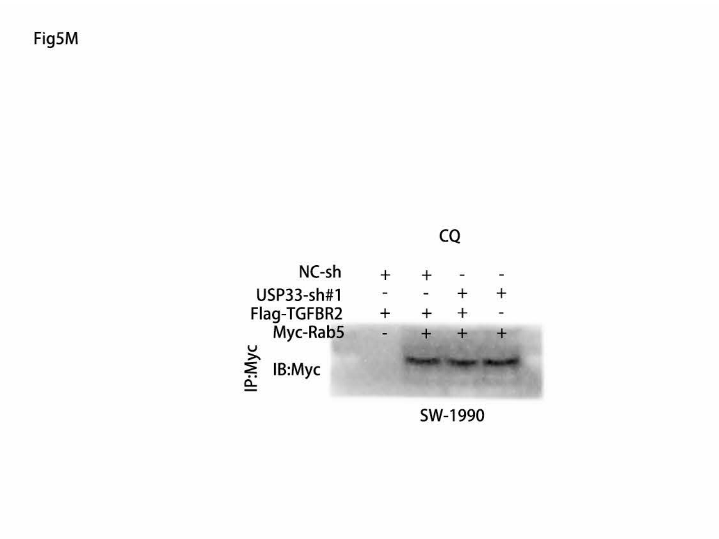
Fig5M
CQ
NC-sh + + - -
USP33-sh#1 - - + +
Flag-TGFBR2 + + + -
Myc-Rab5 - + + +
IP:Myc
IB:Myc
SW-1990

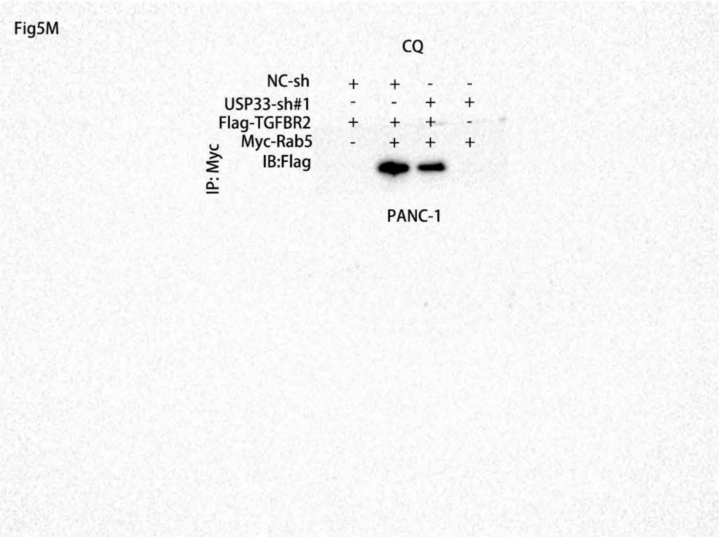
Fig5M
CQ
NC-sh + + - -
USP33-sh#1 - - + +
Flag-TGFBR2 + + + -
Myc-Rab5 - + + +
IP: Myc
IB:Flag
PANC-1

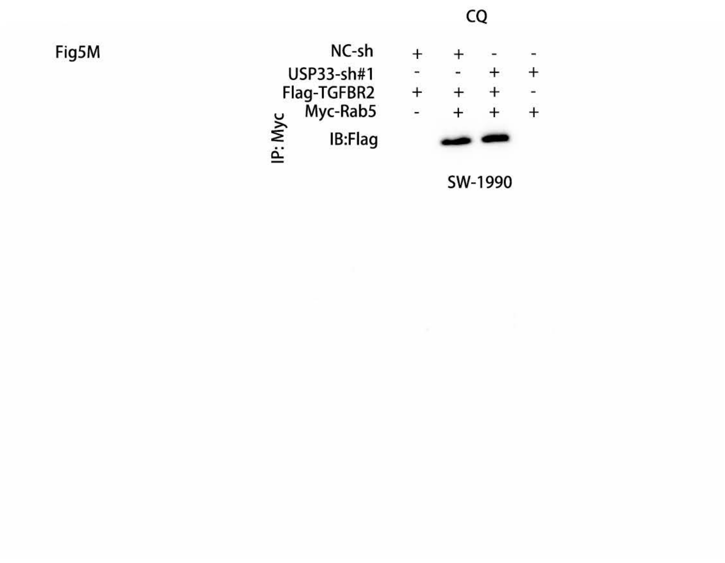
Fig5M
CQ
NC-sh + + - -
USP33-sh#1 - - + +
Flag-TGFBR2 + + + -
Myc-Rab5 - + + +
IP: Myc
IB:Flag
SW-1990

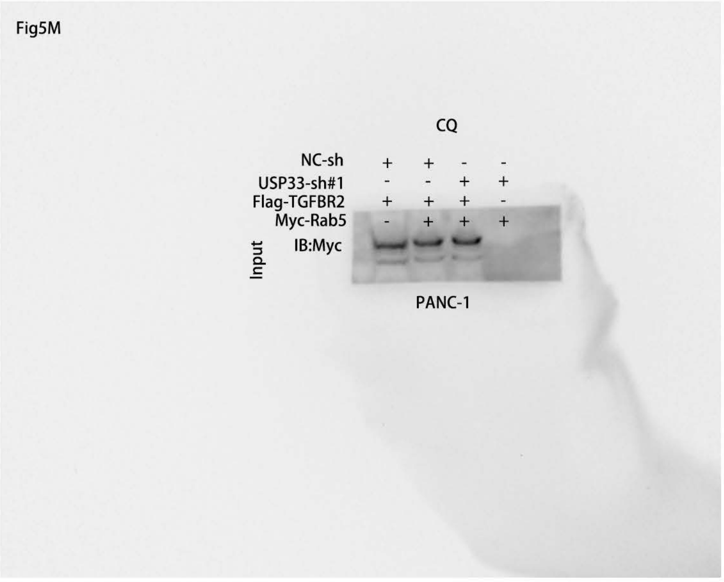
Fig5M
CQ
NC-sh + + - -
USP33-sh#1 - - + +
Flag-TGFBR2 + + + -
Myc-Rab5 - + + +
Input
IB:Myc
PANC-1

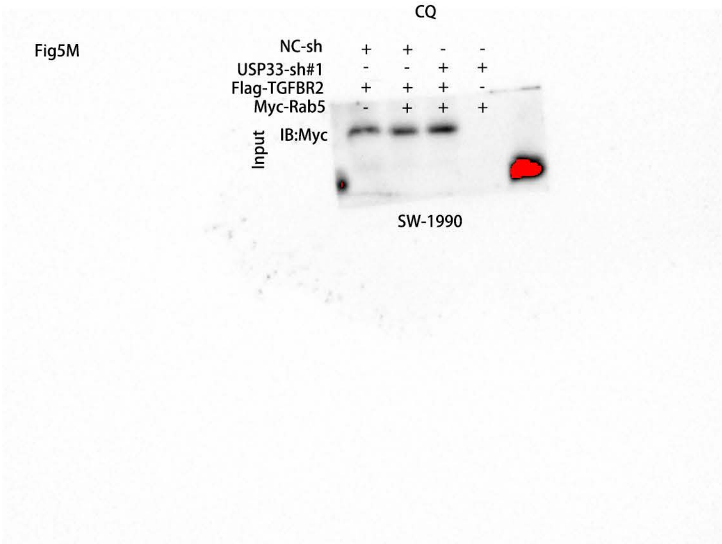
Fig5M
CQ
NC-sh + + - -
USP33-sh#1 - - + +
Flag-TGFBR2 + + + -
Myc-Rab5 - + + +
Input
IB:Myc
SW-1990

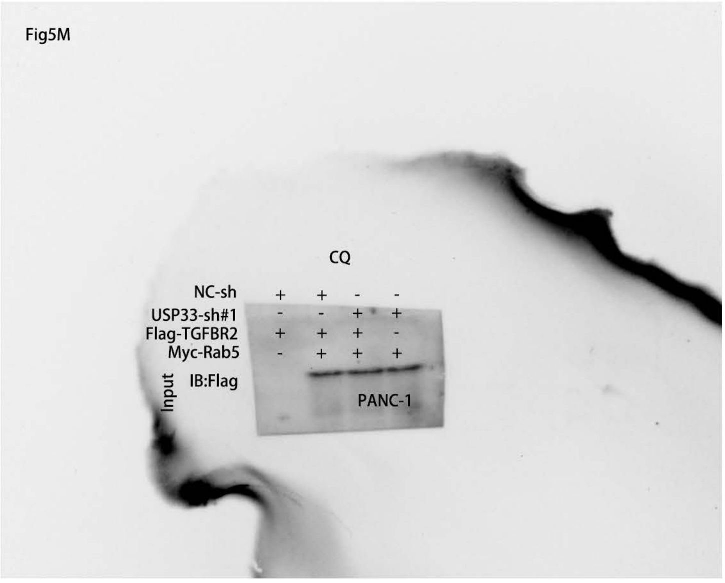
Fig5M
CQ
NC-sh + + - -
USP33-sh#1 - - + +
Flag-TGFBR2 + + + -
Myc-Rab5 - + + +
Input
IB:Flag
PANC-1

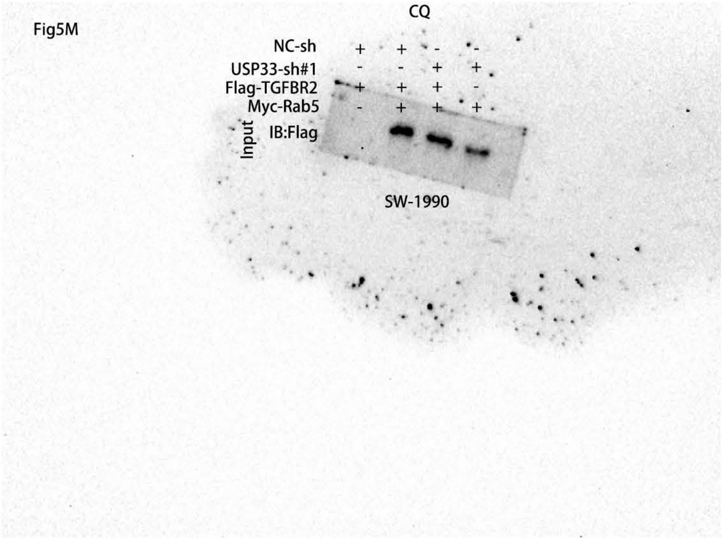
Fig5M
CQ
NC-sh + + - -
USP33-sh#1 - - + +
Flag-TGFBR2 + + + -
Myc-Rab5 - + + +
Input
IB:Flag
SW-1990

Fig5M

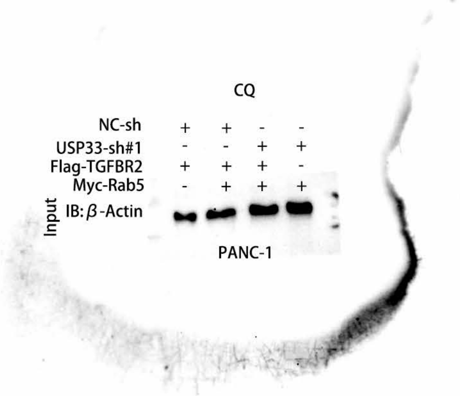

Fig5M

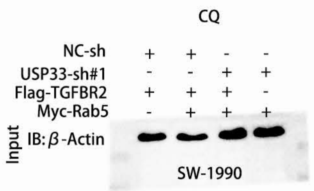

Fig5N

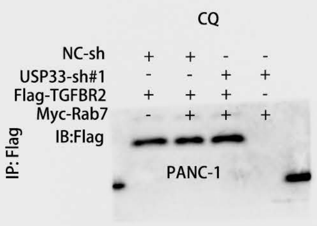

Fig5N

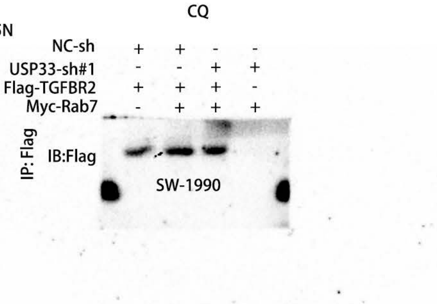

Fig5N

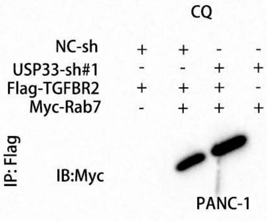

Fig5N

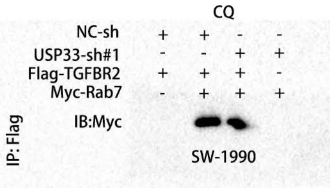

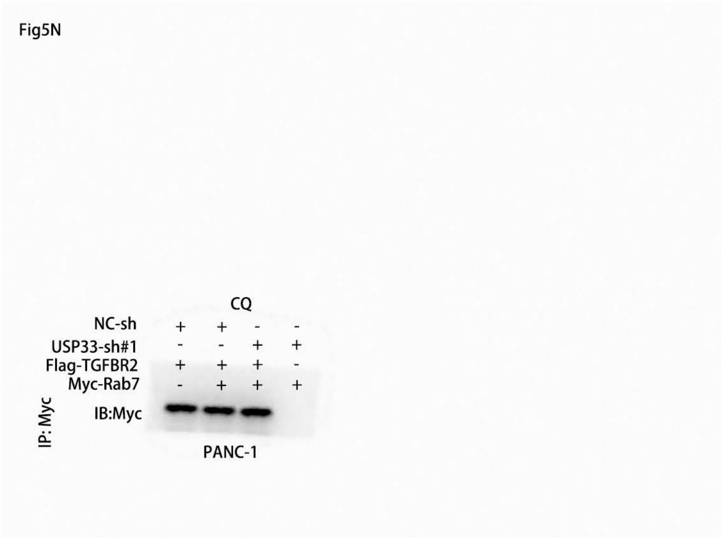
Fig5N
CQ
NC-sh + + - -
USP33-sh#1 - - + +
Flag-TGFBR2 + + + -
Myc-Rab7 - + + +
IP: Myc
IB:Myc
PANC-1

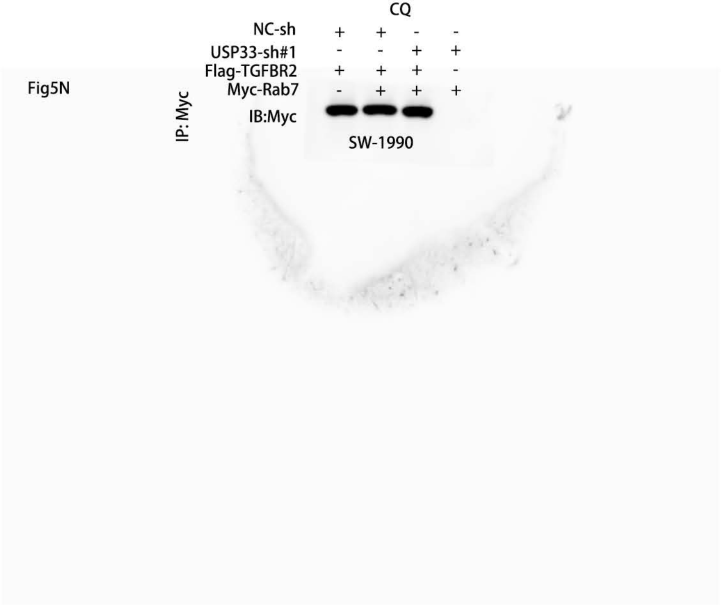
Fig5N
CQ
NC-sh + + - -
USP33-sh#1 - - + +
Flag-TGFBR2 + + + -
Myc-Rab7 - + + +
IP: Myc
IB:Myc
SW-1990

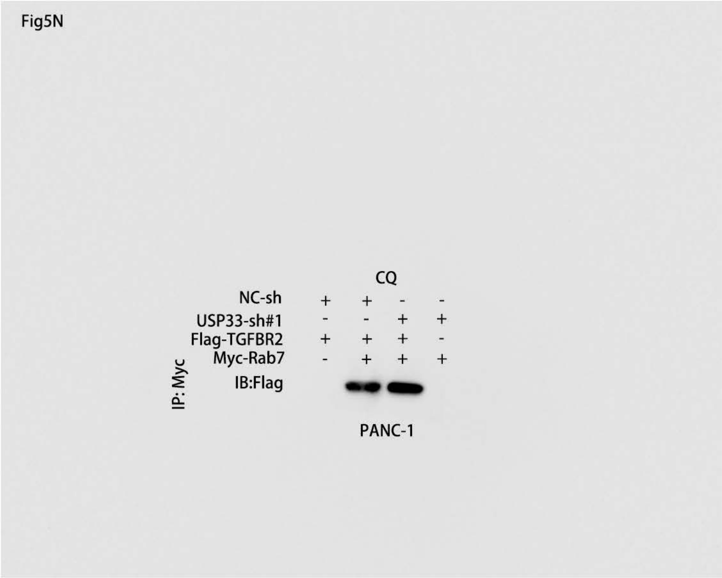
Fig5N
CQ
NC-sh + + - -
USP33-sh#1 - - + +
Flag-TGFBR2 + + + -
Myc-Rab7 - + + +
IP: Myc
IB:Flag
PANC-1

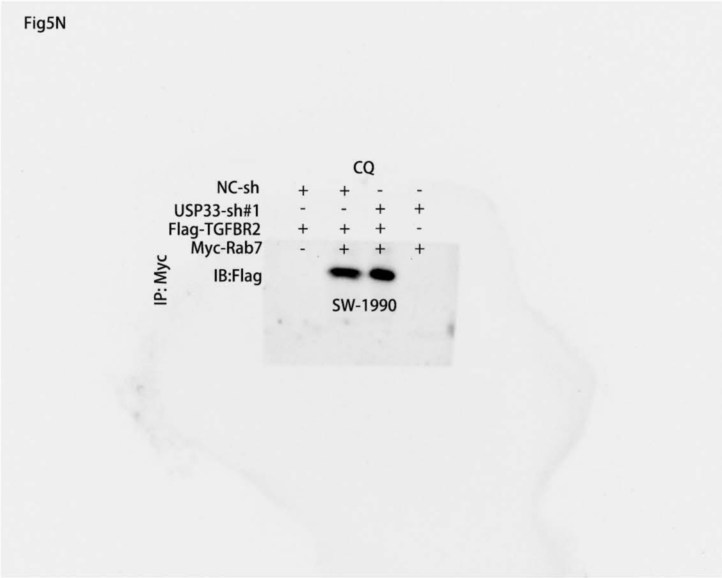
Fig5N
CQ
NC-sh + + - -
USP33-sh#1 - - + +
Flag-TGFBR2 + + + -
Myc-Rab7 - + + +
IP: Myc
IB:Flag
SW-1990

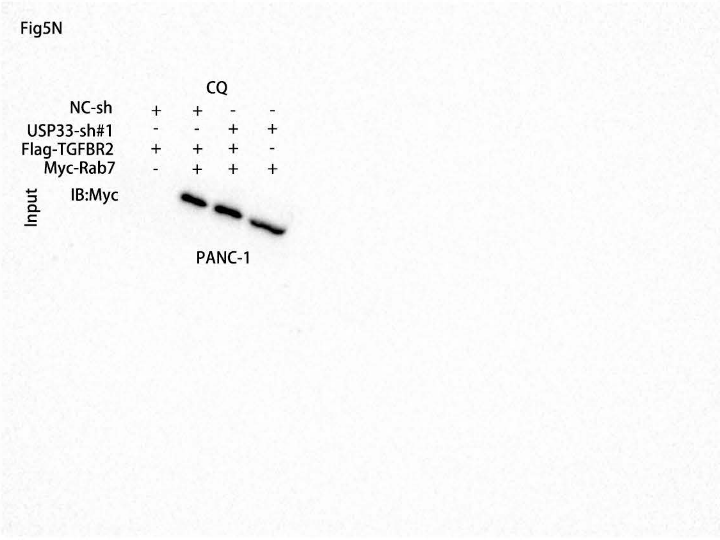
Fig5N
CQ
NC-sh + + - -
USP33-sh#1 - - + +
Flag-TGFBR2 + + + -
Myc-Rab7 - + + +
Input
IB:Myc
PANC-1

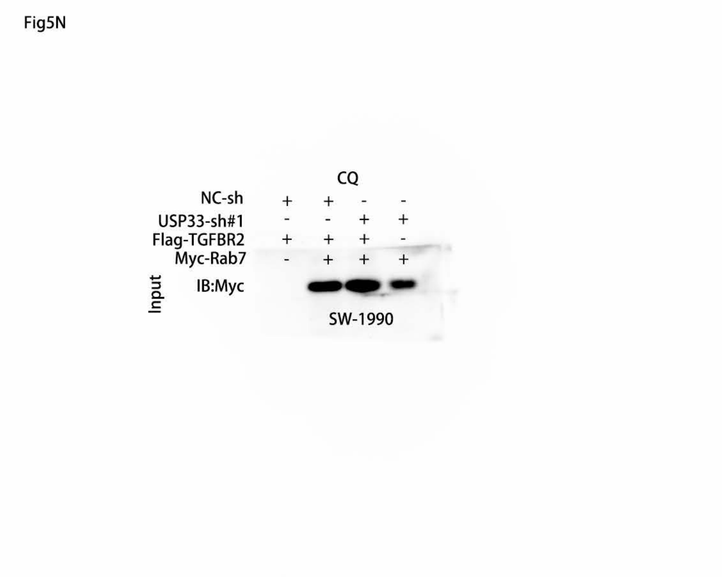
Fig5N
CQ
NC-sh + + - -
USP33-sh#1 - - + +
Flag-TGFBR2 + + + -
Myc-Rab7 - + + +
Input
IB:Myc
SW-1990

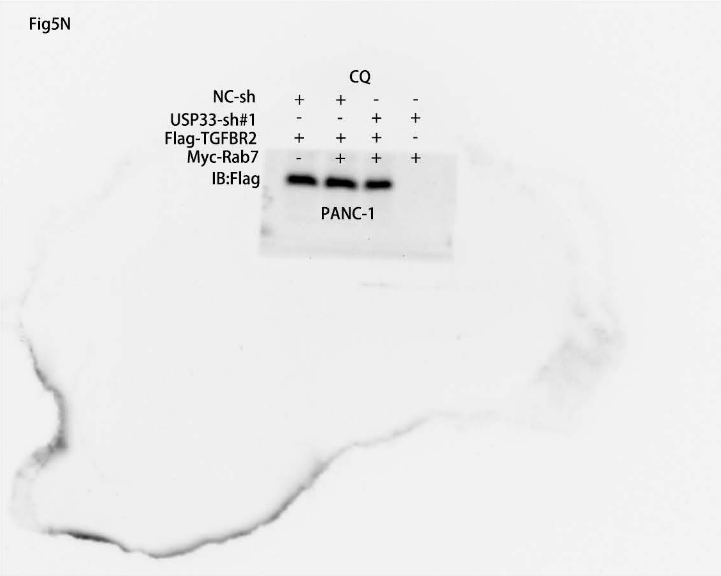
Fig5N
CQ
NC-sh + + - -
USP33-sh#1 - - + +
Flag-TGFBR2 + + + -
Myc-Rab7 - + + +
IB:Flag
PANC-1

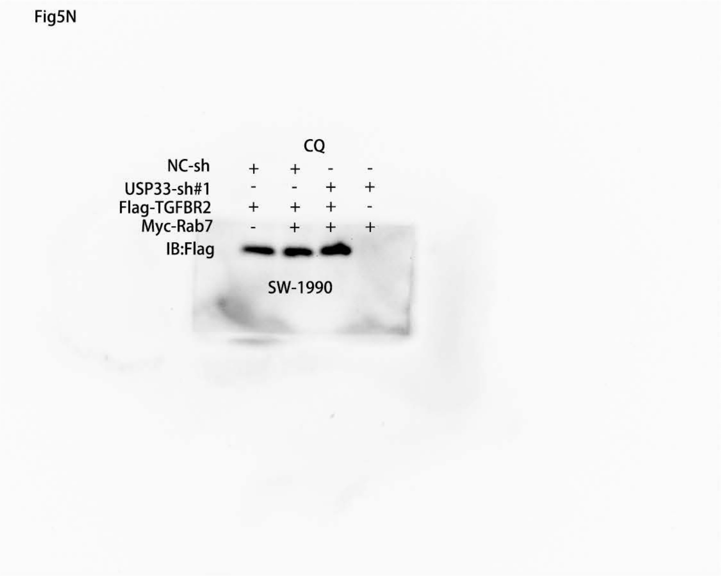
Fig5N
CQ
NC-sh + + - -
USP33-sh#1 - - + +
Flag-TGFBR2 + + + -
Myc-Rab7 - + + +
IB:Flag
SW-1990

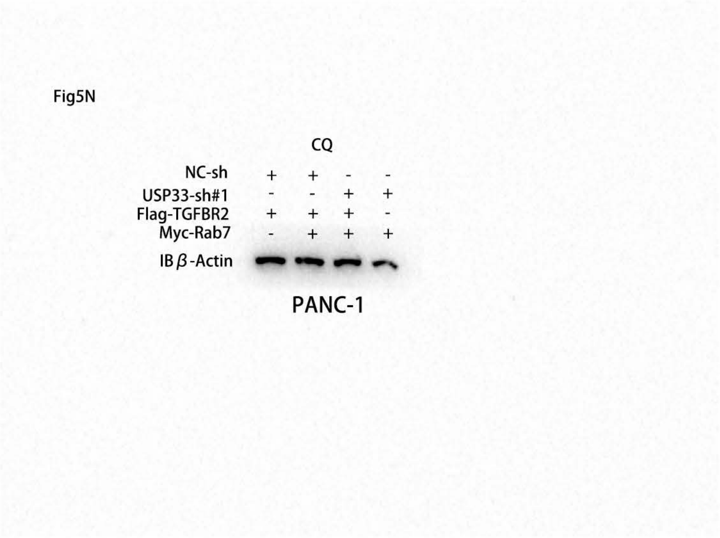
Fig5N
CQ
NC-sh + + - -
USP33-sh#1 - - + +
Flag-TGFBR2 + + + -
Myc-Rab7 - + + +
IB β-Actin
PANC-1

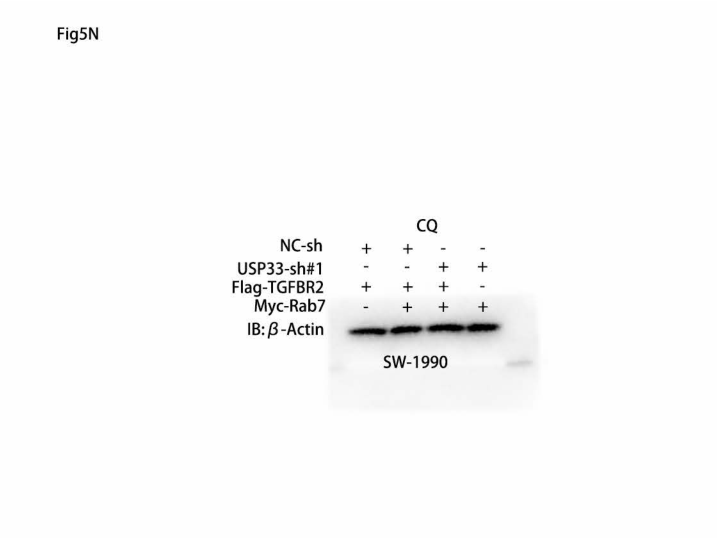
Fig5N
CQ
NC-sh + + - -
USP33-sh#1 - - + +
Flag-TGFBR2 + + + -
Myc-Rab7 - + + +
IB: β-Actin
SW-1990

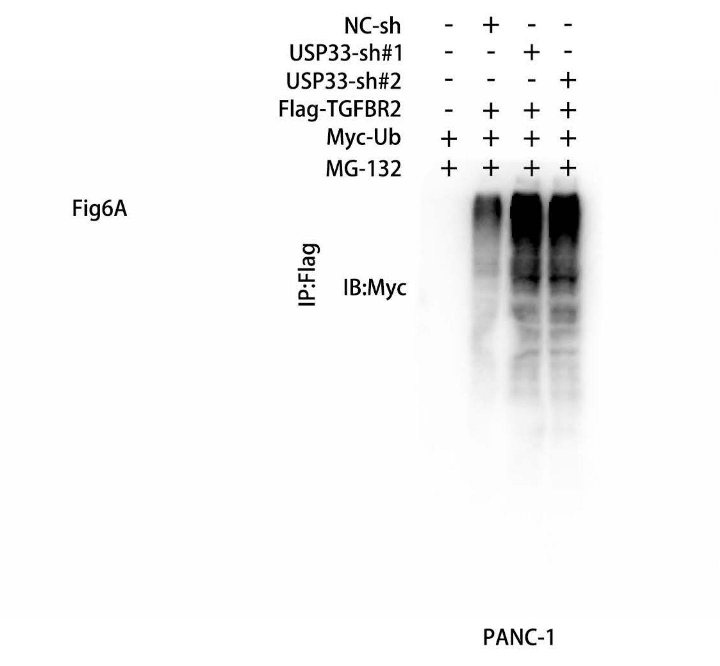
NC-sh - + - -
USP33-sh#1 - - + -
USP33-sh#2 - - - +
Flag-TGFBR2 - + + +
Myc-Ub + + + +
MG-132 + + + +
Fig6A
IP:Flag
IB:Myc
PANC-1

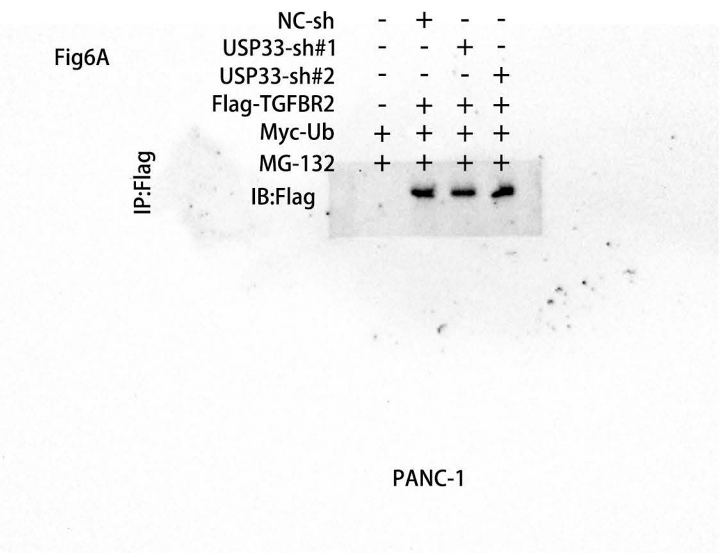
NC-sh - + - -
USP33-sh#1 - - + -
USP33-sh#2 - - - +
Flag-TGFBR2 - + + +
Myc-Ub + + + +
MG-132 + + + +
Fig6A
IP:Flag
IB:Flag
PANC-1

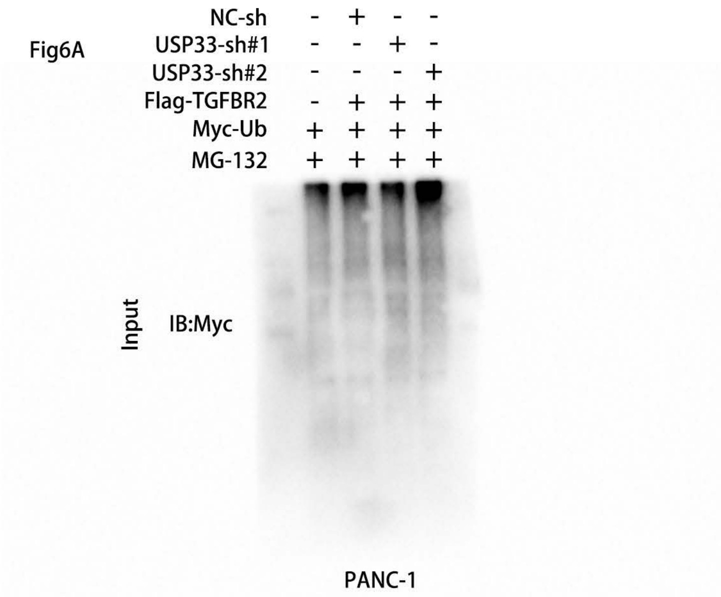
Fig6A
NC-sh - + - -
USP33-sh#1 - - + -
USP33-sh#2 - - - +
Flag-TGFBR2 - + + +
Myc-Ub + + + +
MG-132 + + + +
Input
IB:Myc
PANC-1

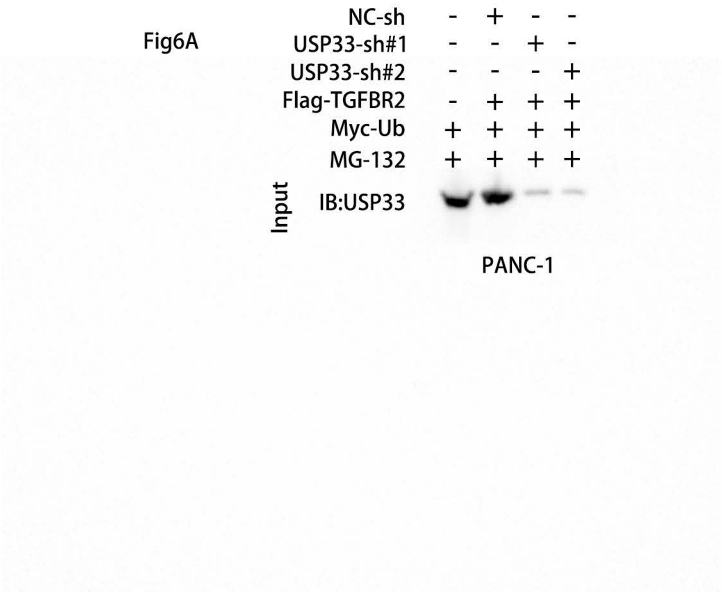
Fig6A
NC-sh - + - -
USP33-sh#1 - - + -
USP33-sh#2 - - - +
Flag-TGFBR2 - + + +
Myc-Ub + + + +
MG-132 + + + +
Input
IB:USP33
PANC-1

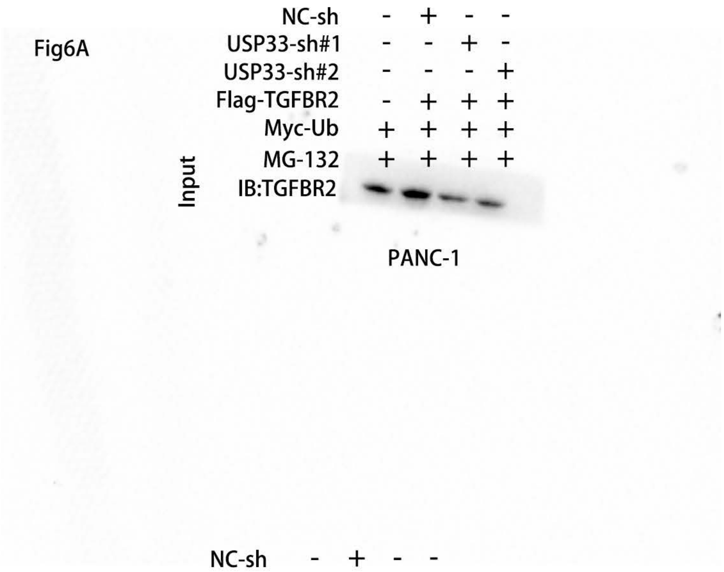
Fig6A
NC-sh - + - -
USP33-sh#1 - - + -
USP33-sh#2 - - - +
Flag-TGFBR2 - + + +
Myc-Ub + + + +
MG-132 + + + +
Input
IB:TGFBR2
PANC-1

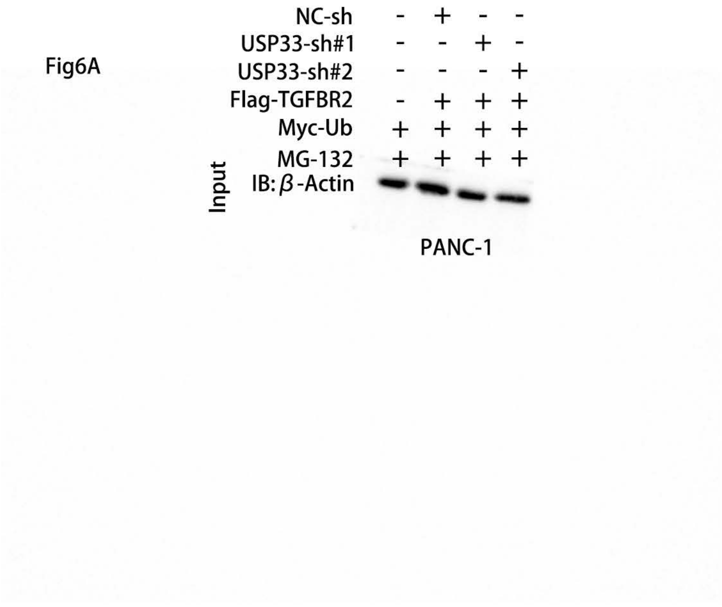
Fig6A
NC-sh - + - -
USP33-sh#1 - - + -
USP33-sh#2 - - - +
Flag-TGFBR2 - + + +
Myc-Ub + + + +
MG-132 + + + +
Input
IB:β-Actin
PANC-1

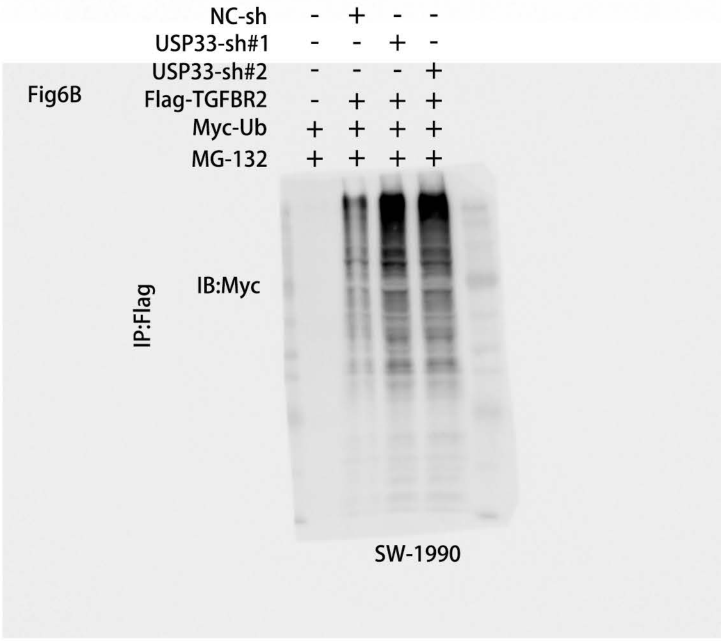
Fig6B
NC-sh - + - -
USP33-sh#1 - - + -
USP33-sh#2 - - - +
Flag-TGFBR2 - + + +
Myc-Ub + + + +
MG-132 + + + +
IP:Flag
IB:Myc
SW-1990

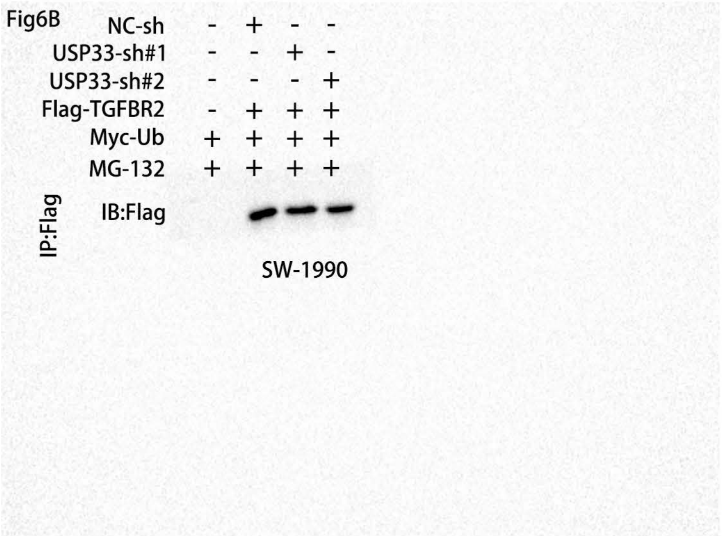
Fig6B
NC-sh - + - -
USP33-sh#1 - - + -
USP33-sh#2 - - - +
Flag-TGFBR2 - + + +
Myc-Ub + + + +
MG-132 + + + +
IP:Flag
IB:Flag
SW-1990

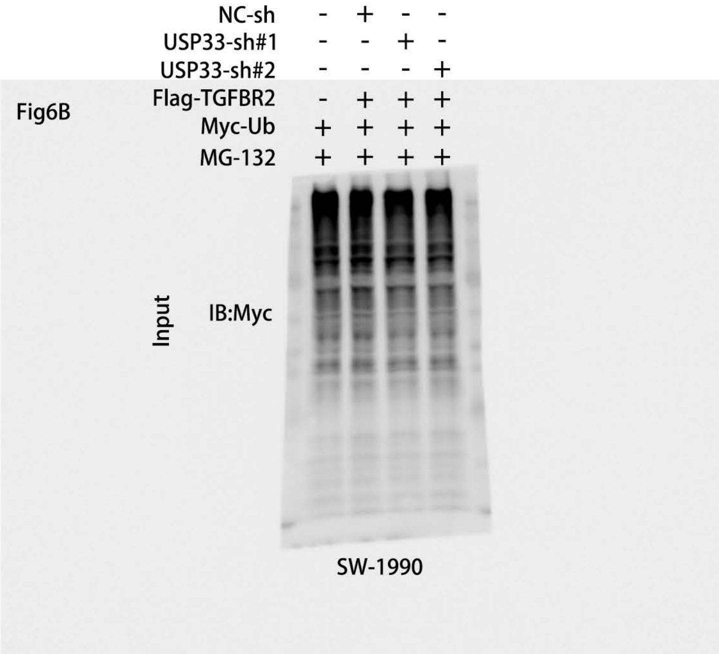
Fig6B
NC-sh - + - -
USP33-sh#1 - - + -
USP33-sh#2 - - - +
Flag-TGFBR2 - + + +
Myc-Ub + + + +
MG-132 + + + +
Input
IB:Myc
SW-1990

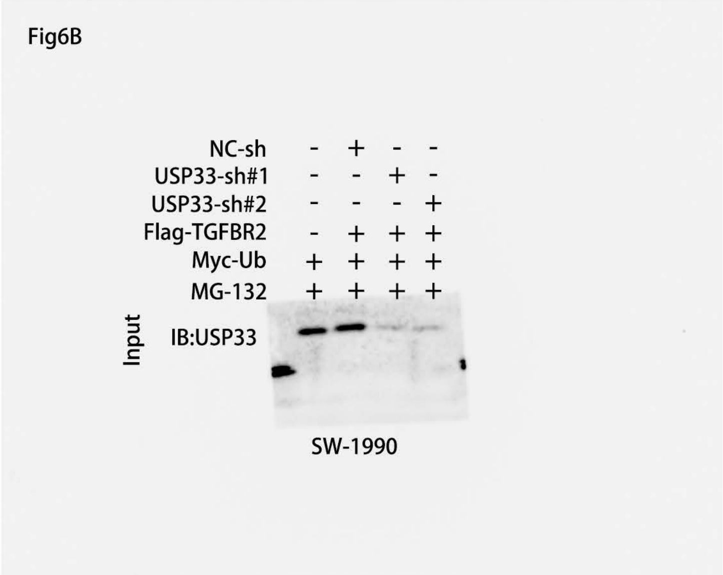
Fig6B
NC-sh - + - -
USP33-sh#1 - - + -
USP33-sh#2 - - - +
Flag-TGFBR2 - + + +
Myc-Ub + + + +
MG-132 + + + +
Input
IB:USP33
SW-1990

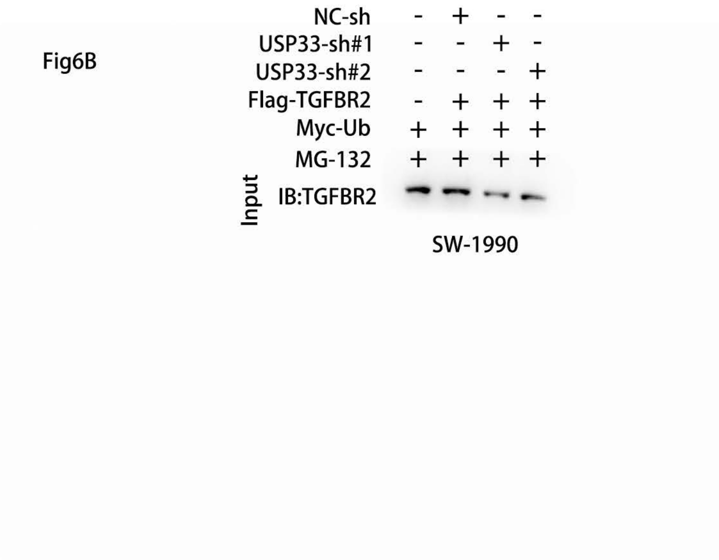
Fig6B
NC-sh - + - -
USP33-sh#1 - - + -
USP33-sh#2 - - - +
Flag-TGFBR2 - + + +
Myc-Ub + + + +
MG-132 + + + +
Input
IB:TGFBR2
SW-1990

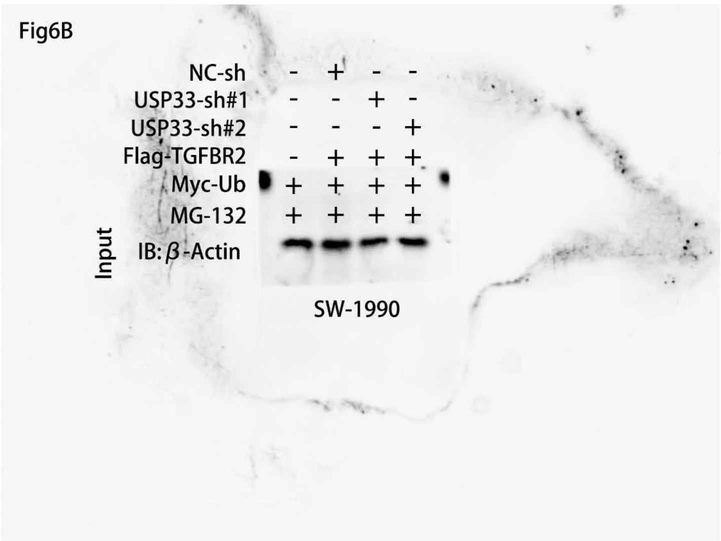
Fig6B
NC-sh - + - -
USP33-sh#1 - - + -
USP33-sh#2 - - - +
Flag-TGFBR2 - + + +
Myc-Ub + + + +
MG-132 + + + +
Input
IB: β-Actin
SW-1990

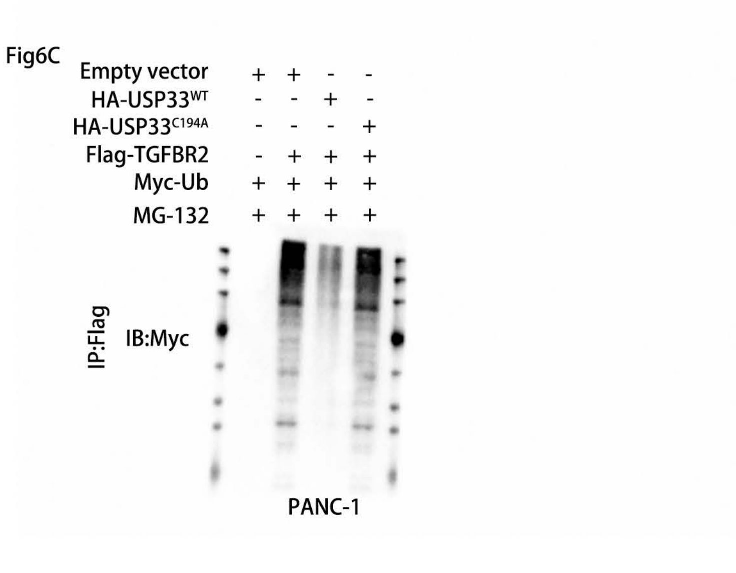
Fig6C
Empty vector + + - -
HA-USP33WT - - + -
HA-USP33C194A - - - +
Flag-TGFBR2 - + + +
Myc-Ub + + + +
MG-132 + + + +
IP:Flag
IB:Myc
PANC-1

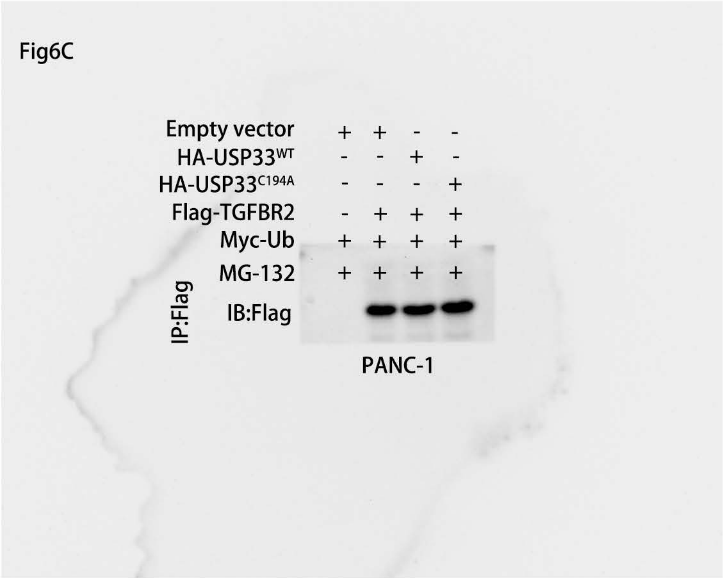
Fig6C
Empty vector + + - -
HA-USP33WT - - + -
HA-USP33C194A - - - +
Flag-TGFBR2 - + + +
Myc-Ub + + + +
MG-132 + + + +
IP:Flag
IB:Flag
PANC-1

Fig6C

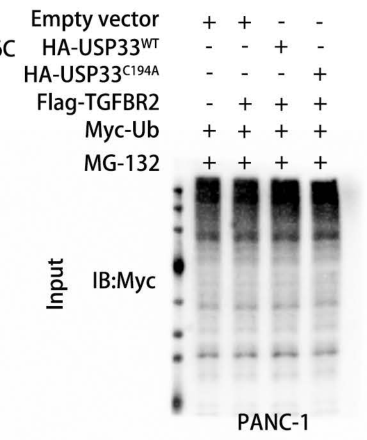

Fig6C

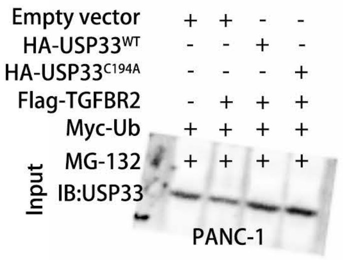

Fig6C

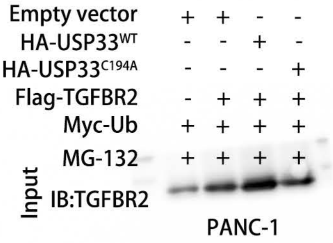

Fig6C

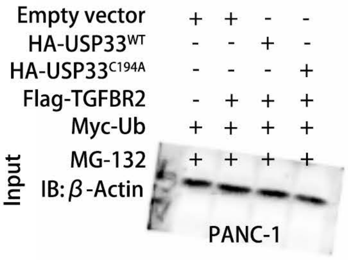

Fig6D

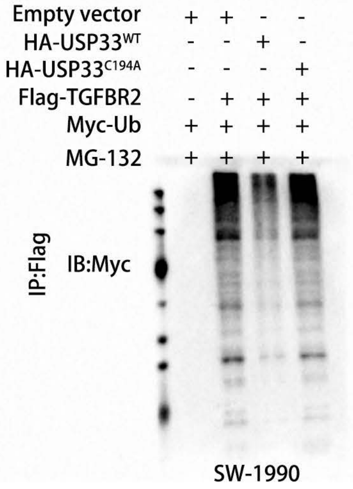

Fig6D

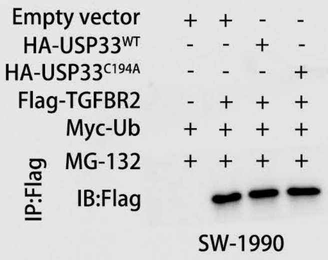

Fig6D

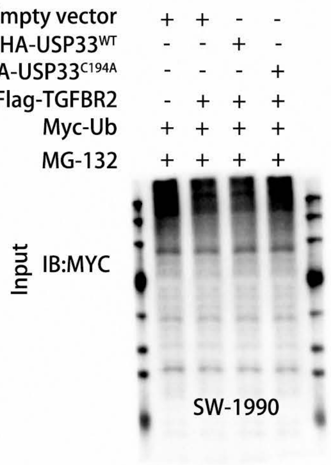

Fig6D

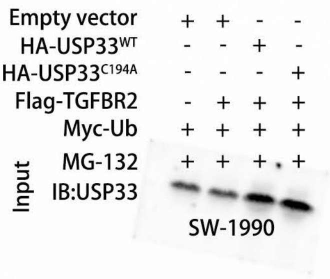

Fig6D

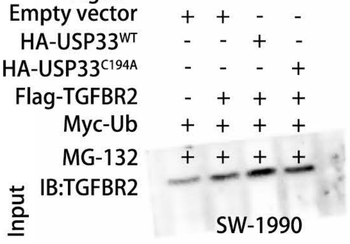

Fig6D

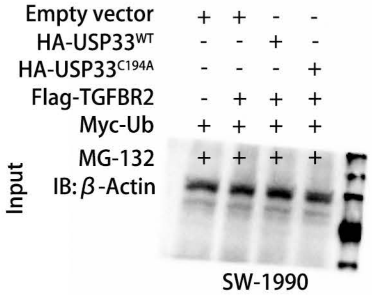

Fig6E

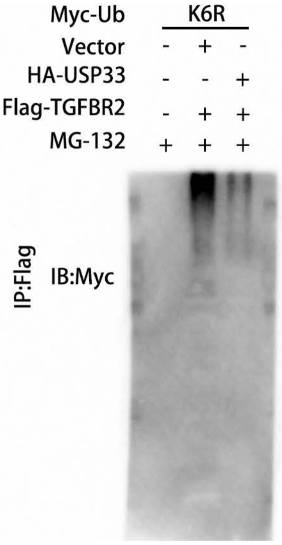

Fig6E

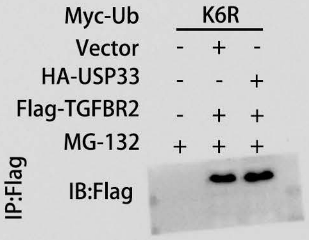

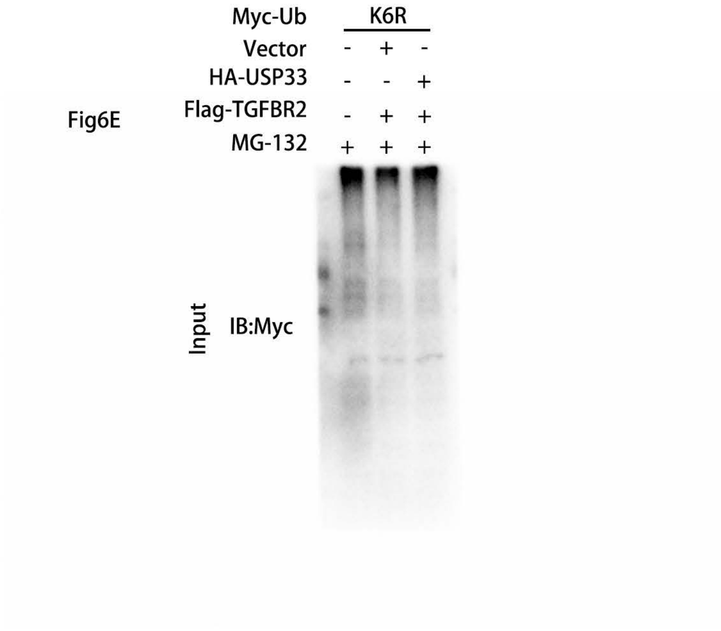
Fig6E
Myc-Ub K6R
Vector - + -
HA-USP33 - - +
Flag-TGFBR2 - + +
MG-132 + + +
Input
IB:Myc

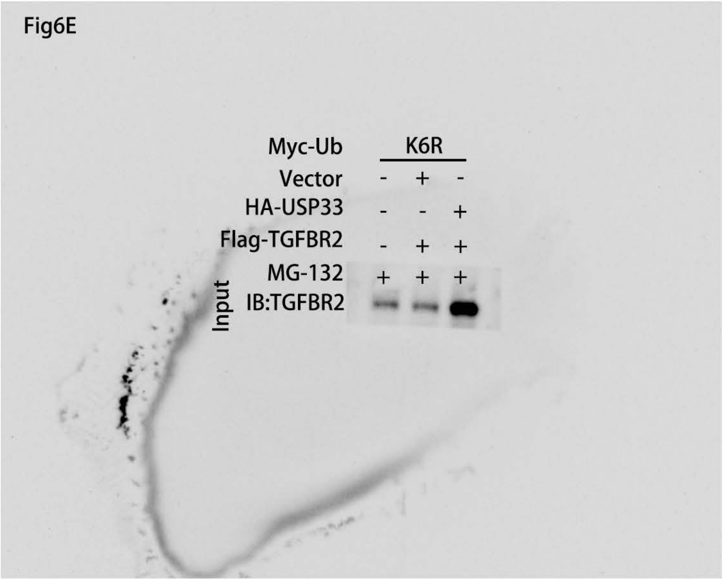
Fig6E
Myc-Ub K6R
Vector - + -
HA-USP33 - - +
Flag-TGFBR2 - + +
MG-132 + + +
Input
IB:TGFBR2

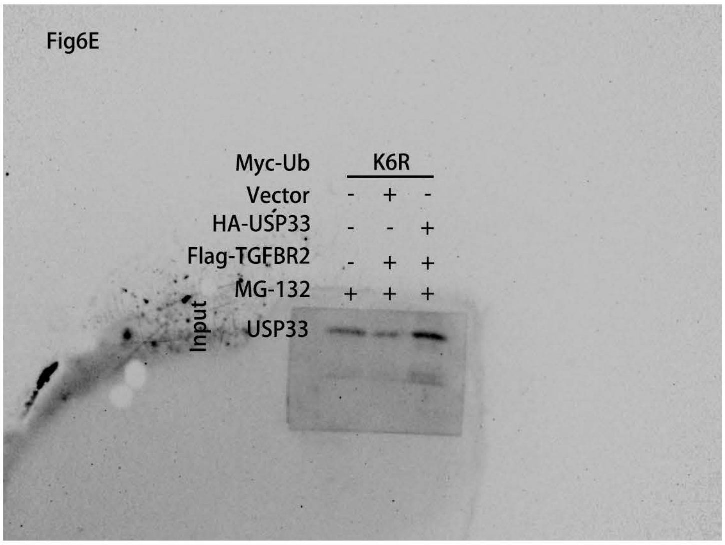
Fig6E
Myc-Ub K6R
Vector - + -
HA-USP33 - - +
Flag-TGFBR2 - + +
MG-132 + + +
Input
USP33

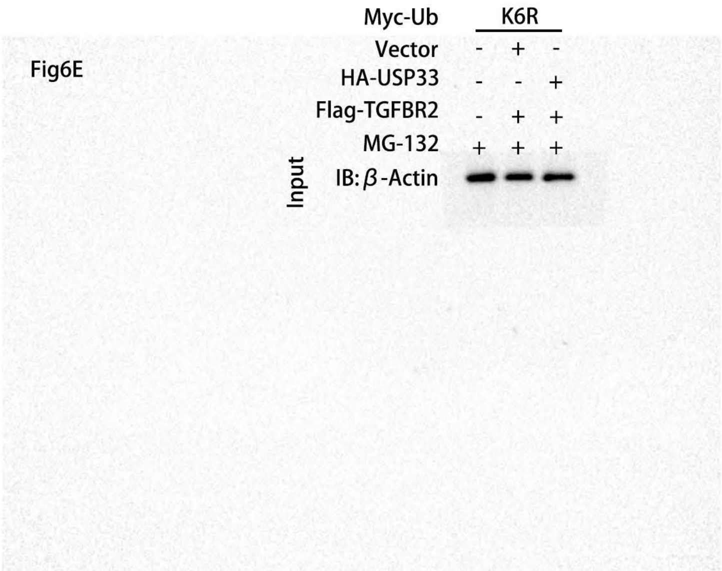
Fig6E
Myc-Ub K6R
Vector - + -
HA-USP33 - - +
Flag-TGFBR2 - + +
MG-132 + + +
Input
IB: β-Actin

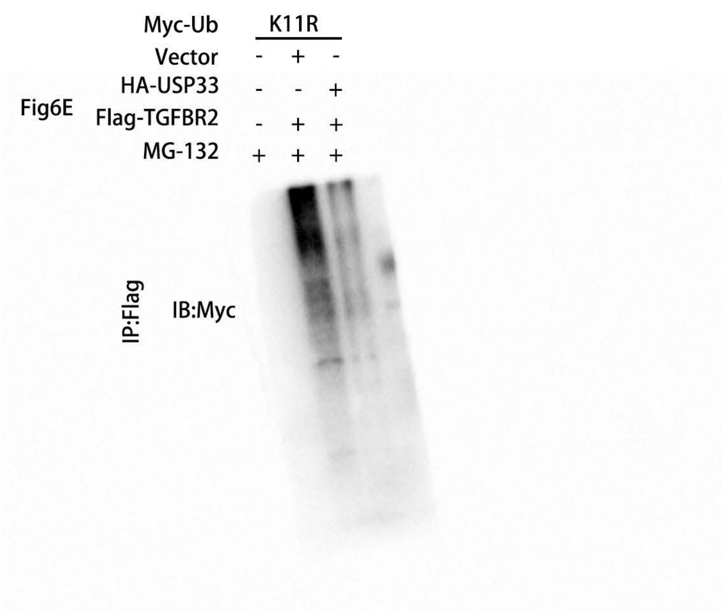
Fig6E
Myc-Ub K11R
Vector - + -
HA-USP33 - - +
Flag-TGFBR2 - + +
MG-132 + + +
IP:Flag
IB:Myc

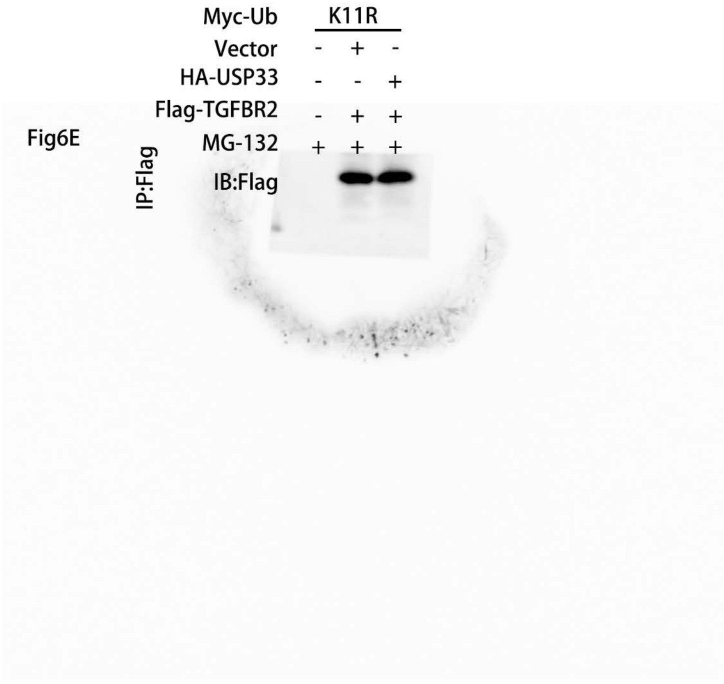
Fig6E
Myc-Ub K11R
Vector - + -
HA-USP33 - - +
Flag-TGFBR2 - + +
MG-132 + + +
IP:Flag
IB:Flag

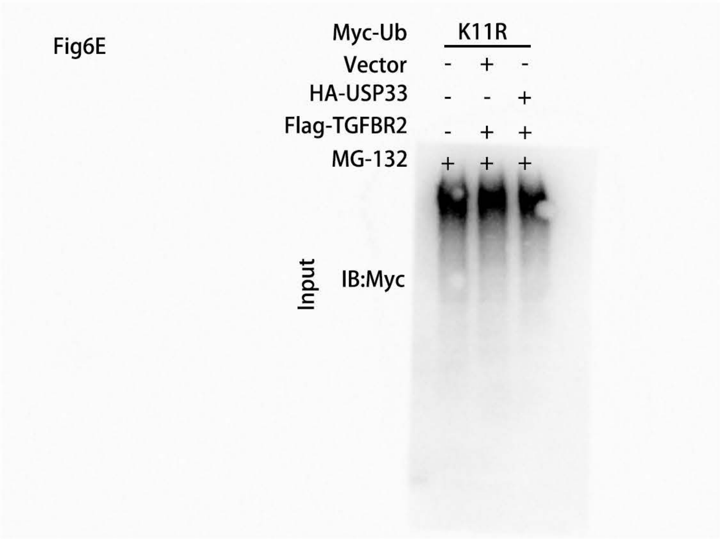
Fig6E
Myc-Ub K11R
Vector - + -
HA-USP33 - - +
Flag-TGFBR2 - + +
MG-132 + + +
Input
IB:Myc

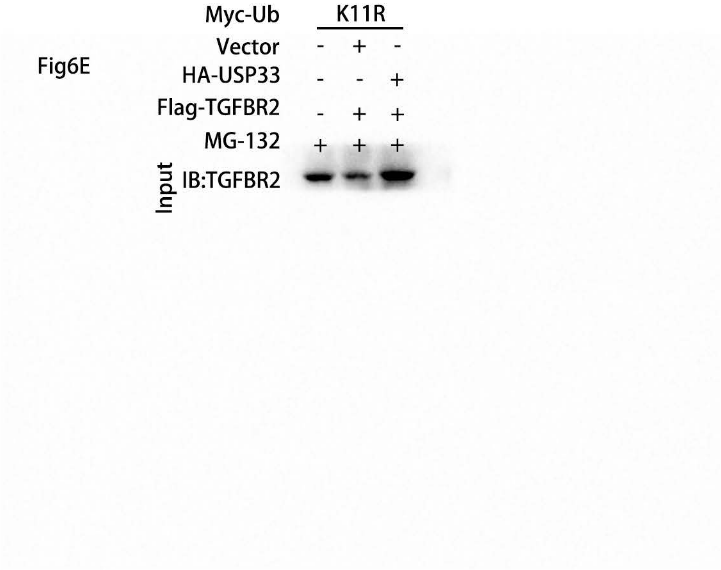
Fig6E
Myc-Ub K11R
Vector - + -
HA-USP33 - - +
Flag-TGFBR2 - + +
MG-132 + + +
Input
IB:TGFBR2

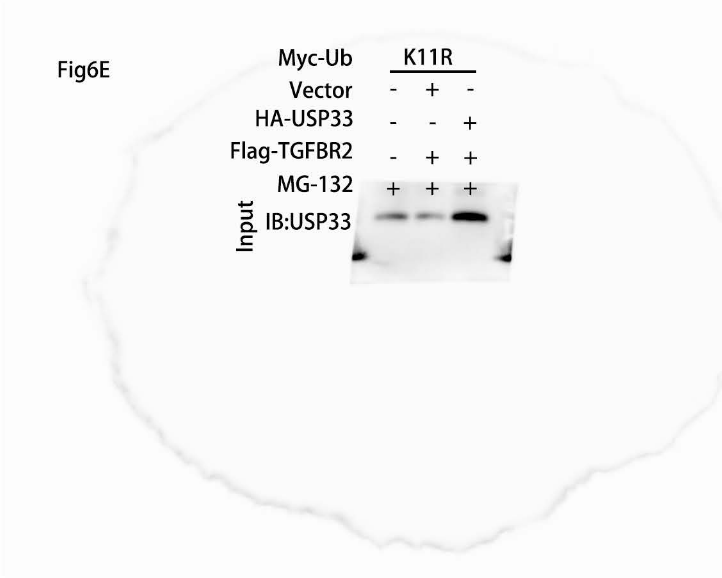
Fig6E
Myc-Ub K11R
Vector - + -
HA-USP33 - - +
Flag-TGFBR2 - + +
MG-132 + + +
Input
IB:USP33

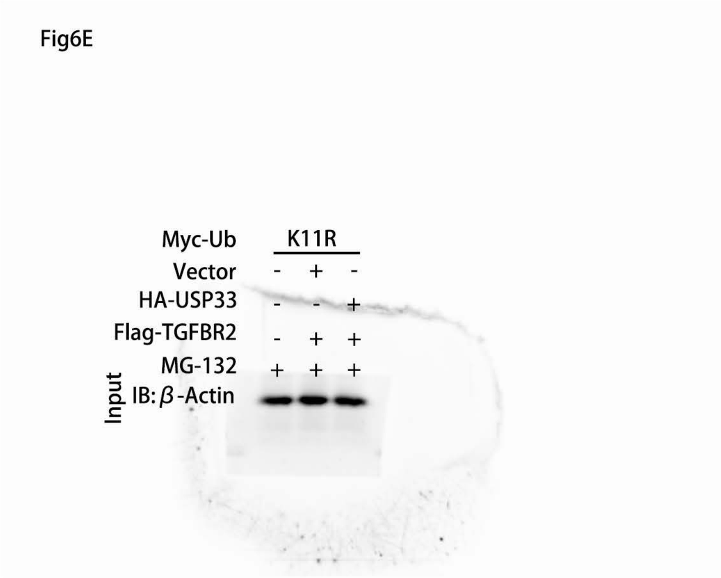
Fig6E
Myc-Ub K11R
Vector - + -
HA-USP33 - - +
Flag-TGFBR2 - + +
MG-132 + + +
Input
IB: β -Actin

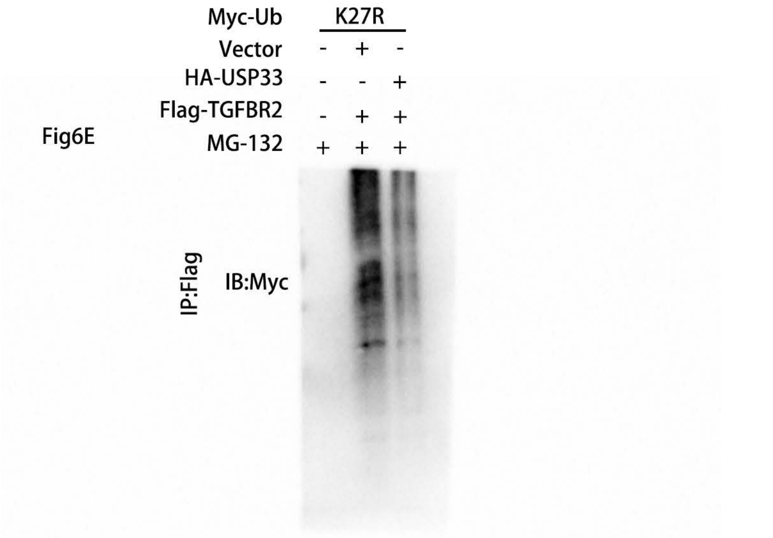
Fig6E
Myc-Ub K27R
Vector - + -
HA-USP33 - - +
Flag-TGFBR2 - + +
MG-132 + + +
IP:Flag
IB:Myc

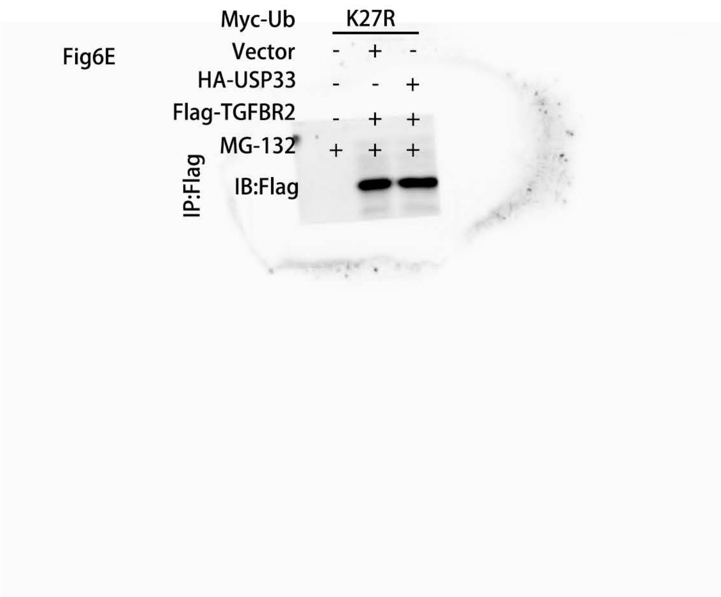
Fig6E
Myc-Ub K27R
Vector - + -
HA-USP33 - - +
Flag-TGFBR2 - + +
MG-132 + + +
IP:Flag
IB:Flag

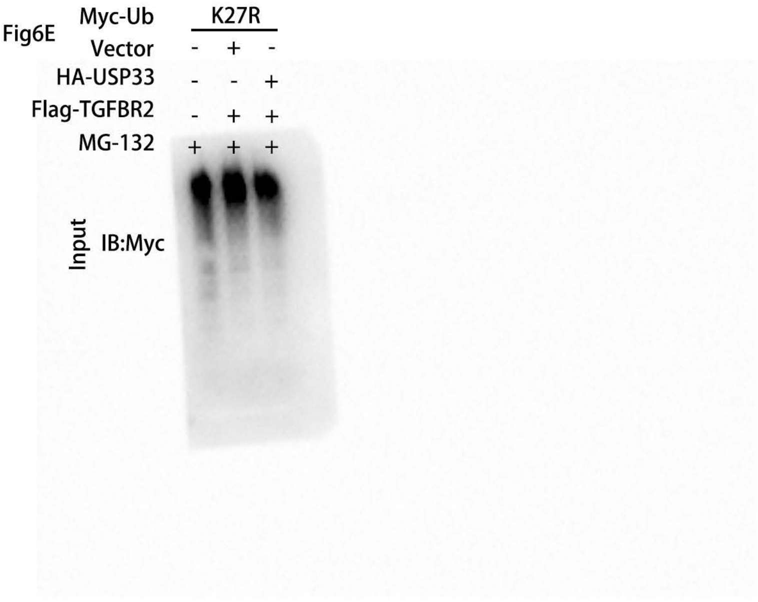
Fig6E
Myc-Ub K27R
Vector - + -
HA-USP33 - - +
Flag-TGFBR2 - + +
MG-132 + + +
Input IB:Myc

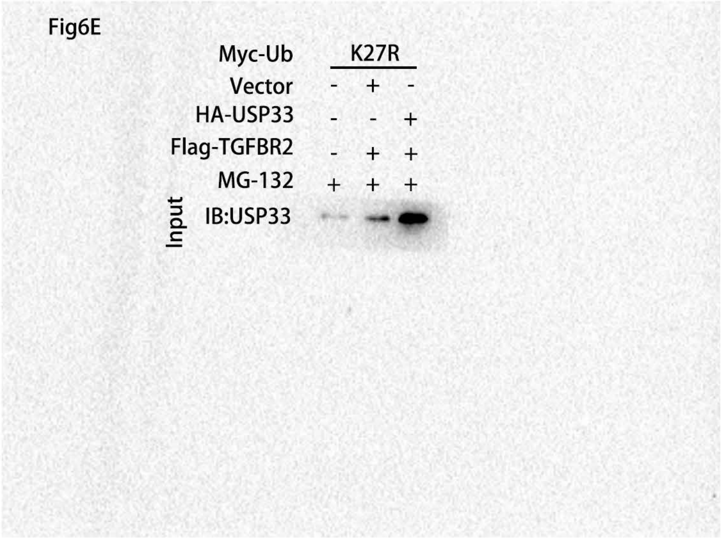
Fig6E
Myc-Ub K27R
Vector - + -
HA-USP33 - - +
Flag-TGFBR2 - + +
MG-132 + + +
Input IB:USP33

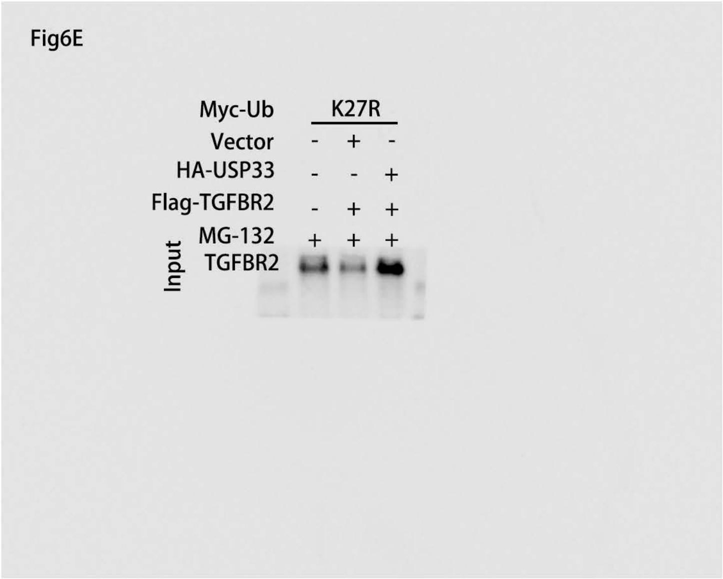
Fig6E
Myc-Ub K27R
Vector - + -
HA-USP33 - - +
Flag-TGFBR2 - + +
MG-132 + + +
Input TGFBR2

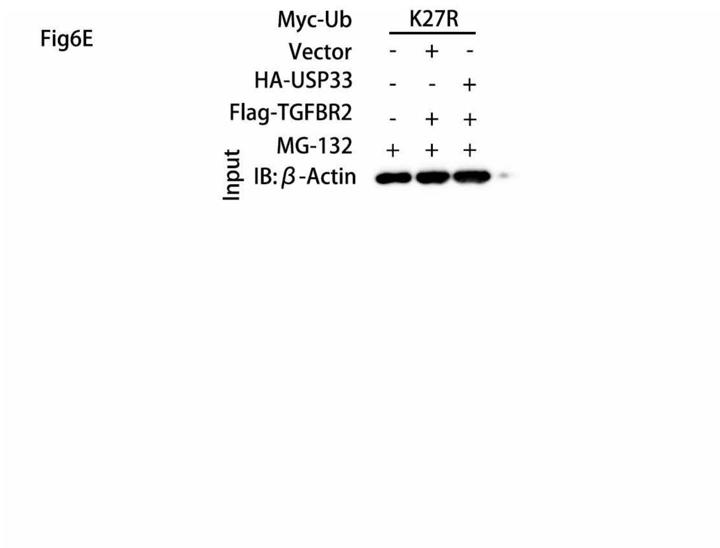
Fig6E
Myc-Ub K27R
Vector - + -
HA-USP33 - - +
Flag-TGFBR2 - + +
MG-132 + + +
Input IB: β-Actin

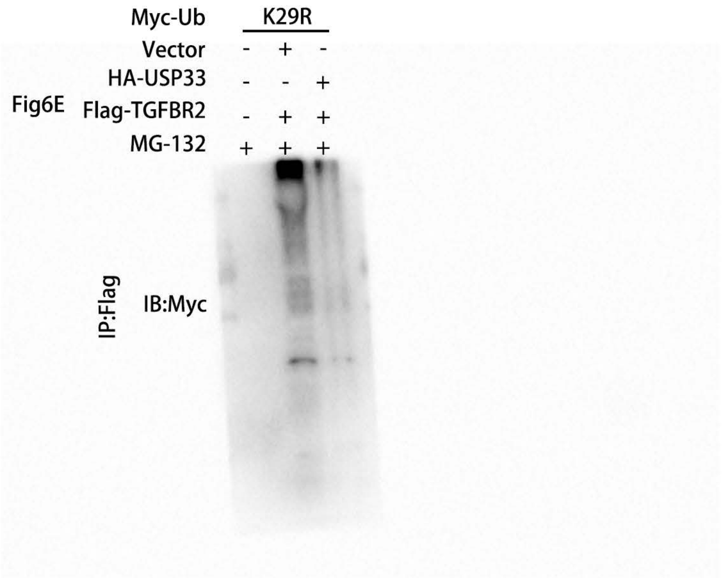
Fig6E
Myc-Ub K29R
Vector - + -
HA-USP33 - - +
Flag-TGFBR2 - + +
MG-132 + + +
IP:Flag IB:Myc

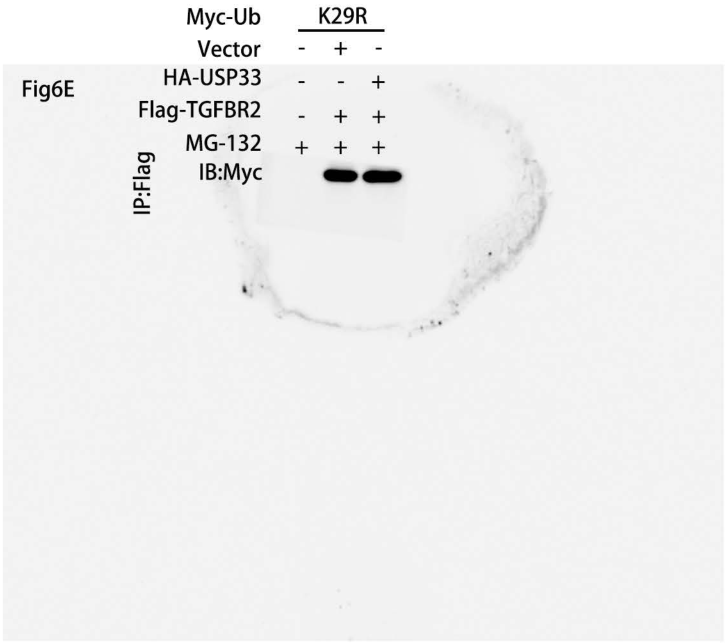
Fig6E
Myc-Ub K29R
Vector - + -
HA-USP33 - - +
Flag-TGFBR2 - + +
MG-132 + + +
IP:Flag IB:Myc

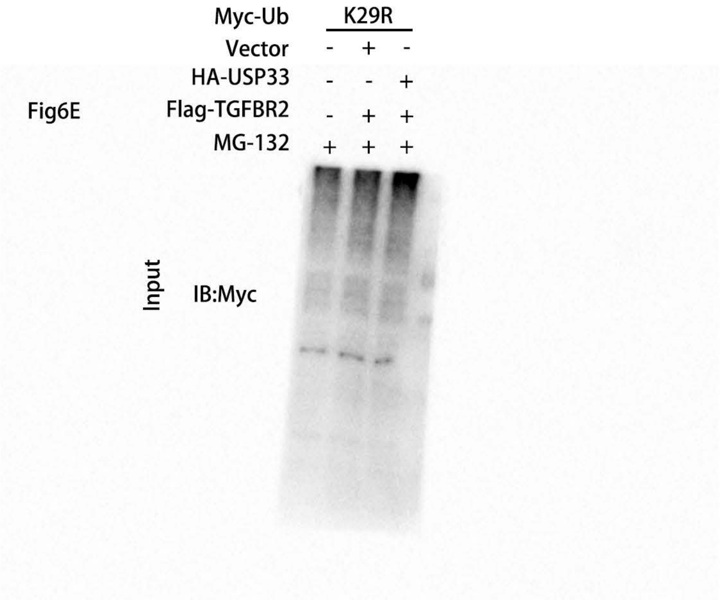
Fig6E
Myc-Ub
K29R
Vector
HA-USP33
Flag-TGFBR2
MG-132
Input
IB:Myc

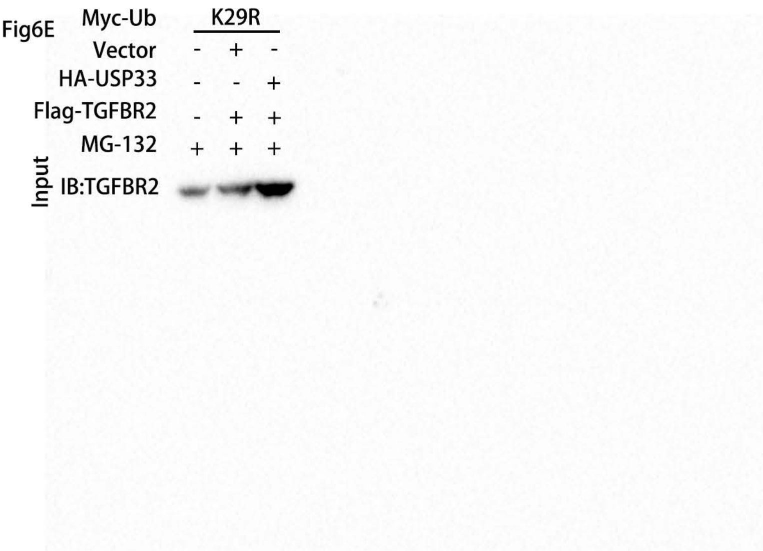
Fig6E
Myc-Ub
K29R
Vector
HA-USP33
Flag-TGFBR2
MG-132
Input
IB:TGFBR2

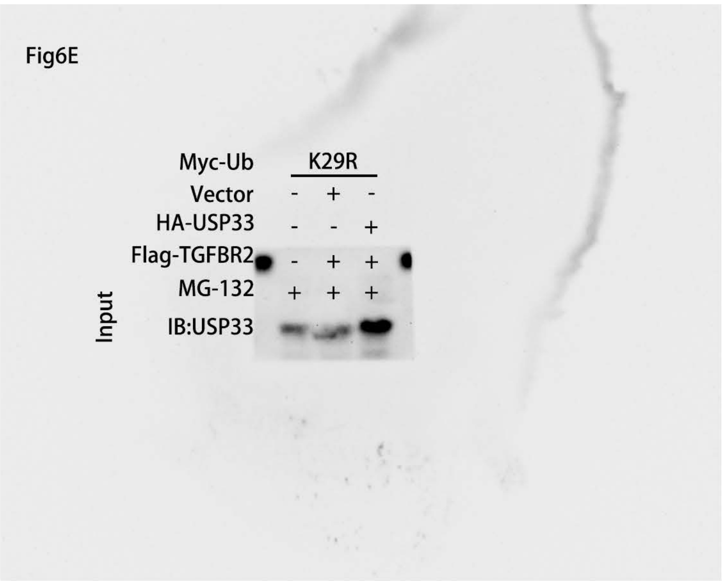
Fig6E
Myc-Ub
K29R
Vector
HA-USP33
Flag-TGFBR2
MG-132
Input
IB:USP33

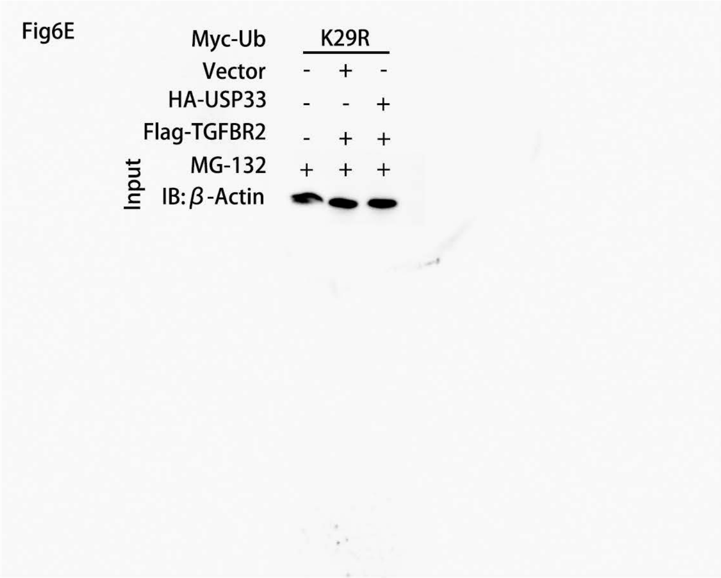
Fig6E
Myc-Ub
K29R
Vector
HA-USP33
Flag-TGFBR2
MG-132
Input
IB: β-Actin

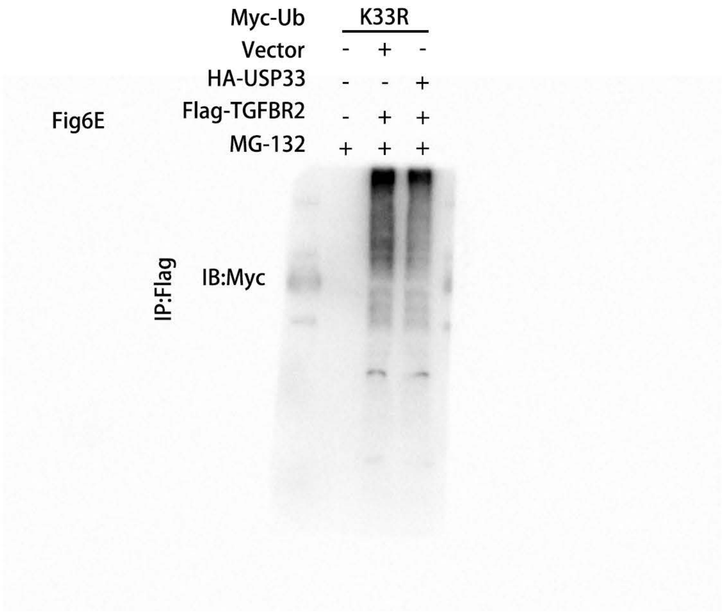
Fig6E
Myc-Ub
K33R
Vector
HA-USP33
Flag-TGFBR2
MG-132
IP:Flag
IB:Myc

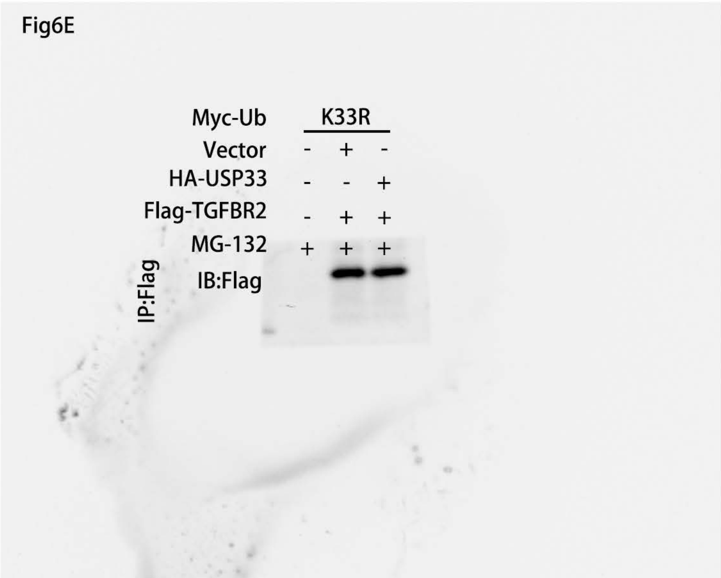
Fig6E
Myc-Ub
K33R
Vector
HA-USP33
Flag-TGFBR2
MG-132
IP:Flag
IB:Flag

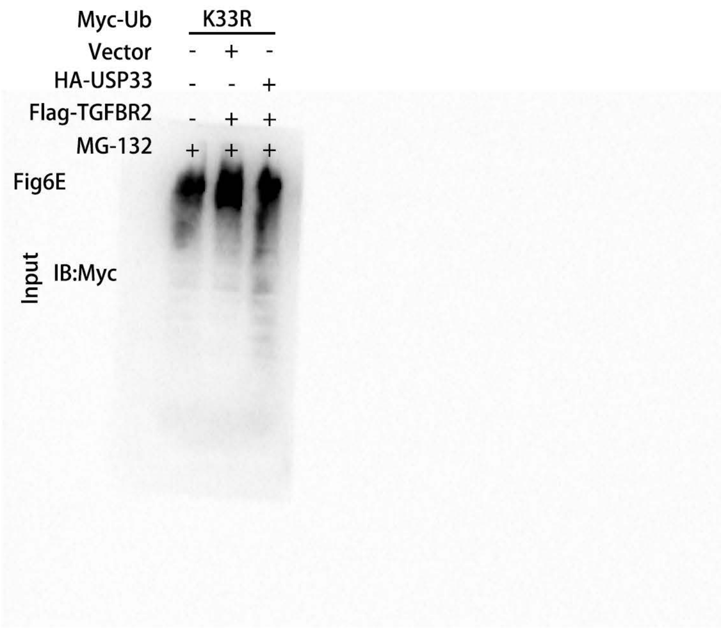
Myc-Ub K33R
Vector - + -
HA-USP33 - - +
Flag-TGFBR2 - + +
MG-132 + + +
Fig6E
Input
IB:Myc

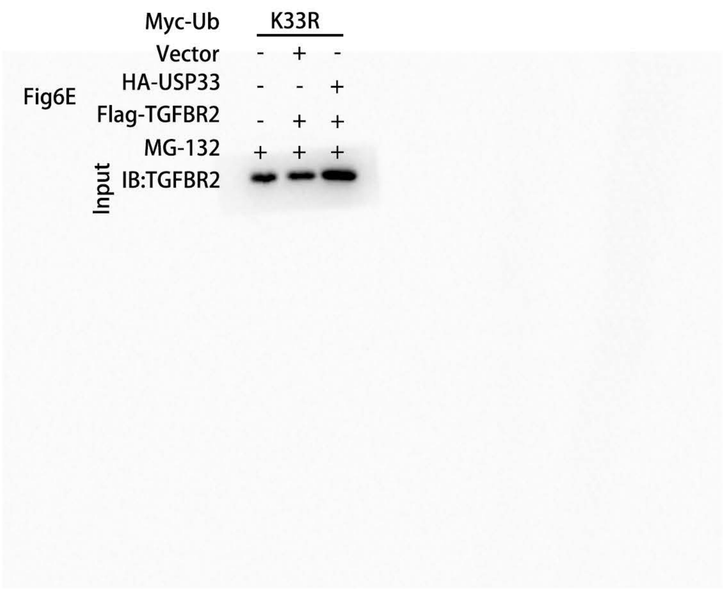
Myc-Ub K33R
Vector - + -
HA-USP33 - - +
Fig6E
Flag-TGFBR2 - + +
MG-132 + + +
Input
IB:TGFBR2

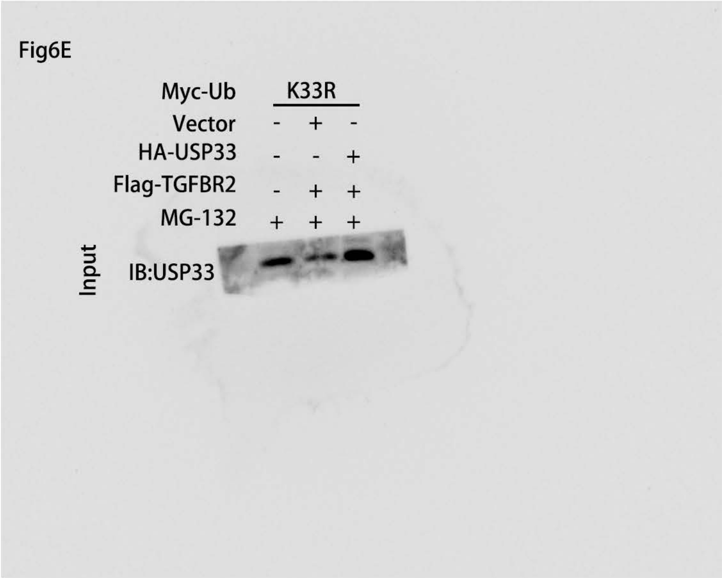
Fig6E
Myc-Ub K33R
Vector - + -
HA-USP33 - - +
Flag-TGFBR2 - + +
MG-132 + + +
Input
IB:USP33

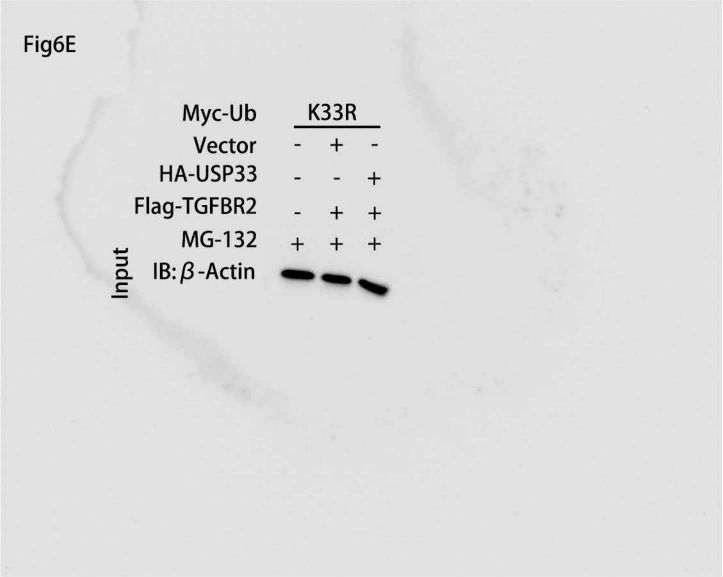
Fig6E
Myc-Ub K33R
Vector - + -
HA-USP33 - - +
Flag-TGFBR2 - + +
MG-132 + + +
Input
IB: β-Actin

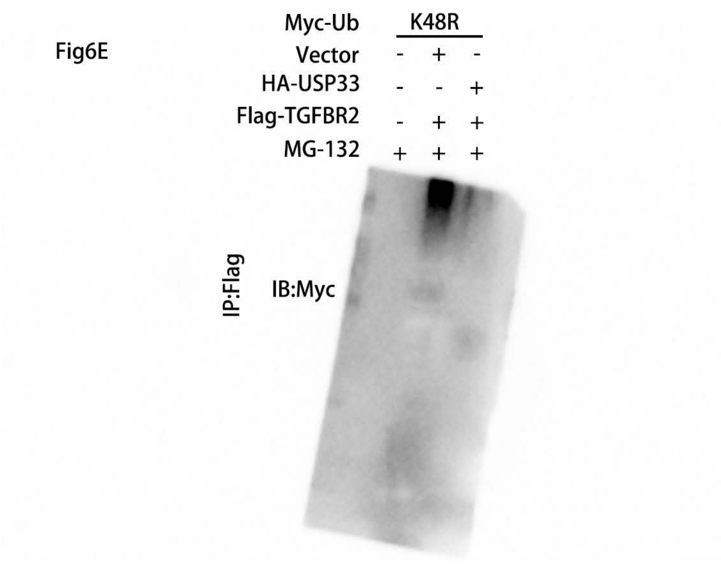
Fig6E
Myc-Ub K48R
Vector - + -
HA-USP33 - - +
Flag-TGFBR2 - + +
MG-132 + + +
IP:Flag
IB:Myc

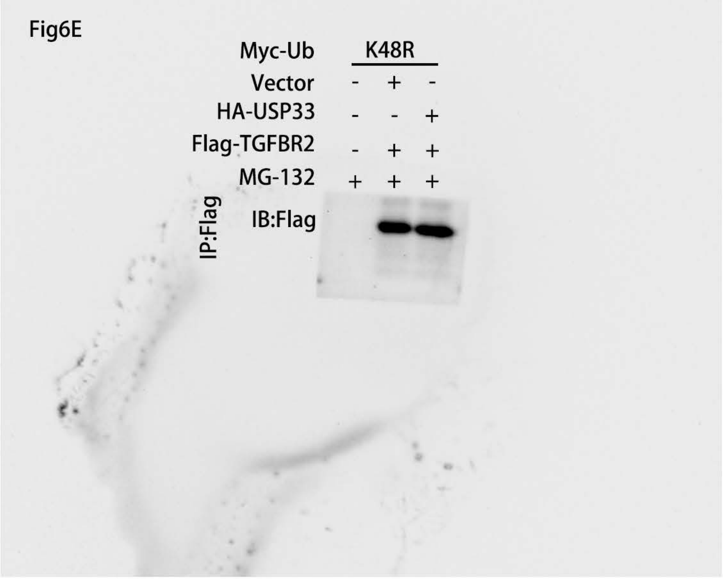
Fig6E
Myc-Ub K48R
Vector - + -
HA-USP33 - - +
Flag-TGFBR2 - + +
MG-132 + + +
IP:Flag
IB:Flag

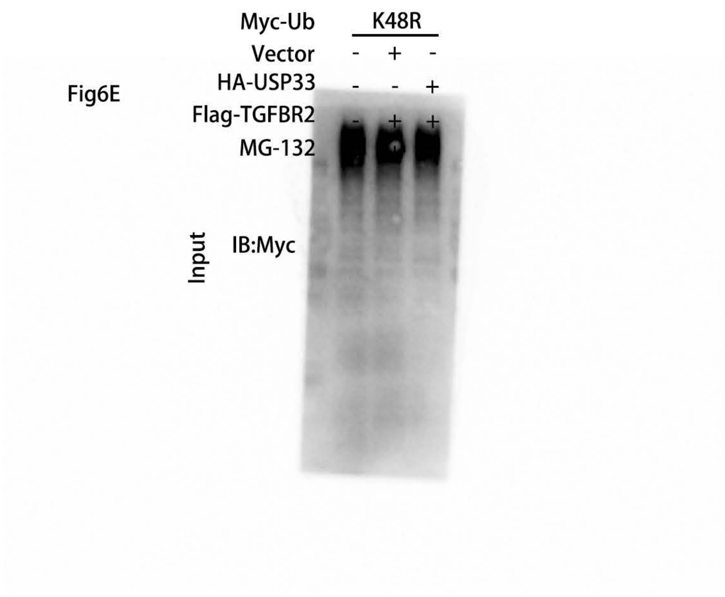
Fig6E
Myc-Ub K48R
Vector - + -
HA-USP33 - - +
Flag-TGFBR2 - + +
MG-132
Input
IB:Myc

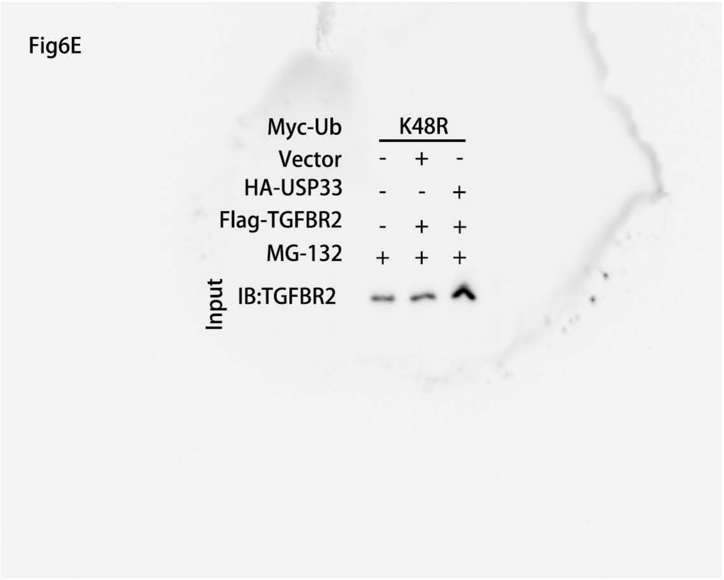
Fig6E
Myc-Ub K48R
Vector - + -
HA-USP33 - - +
Flag-TGFBR2 - + +
MG-132 + + +
Input
IB:TGFBR2

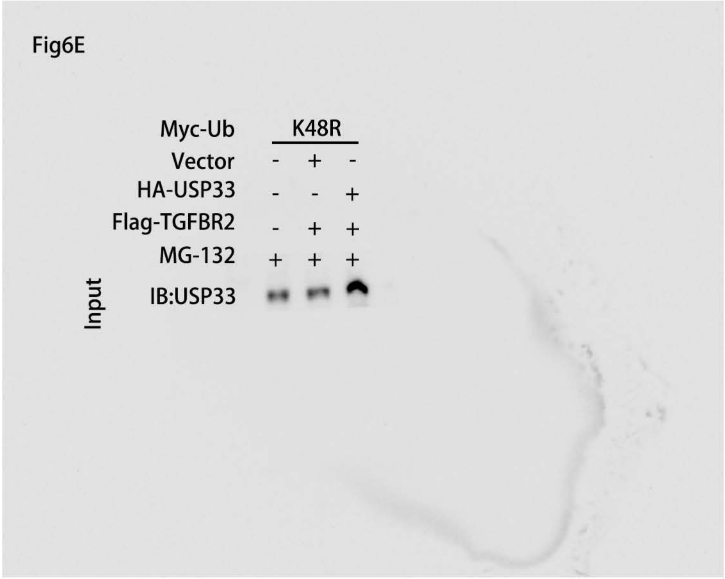
Fig6E
Myc-Ub K48R
Vector - + -
HA-USP33 - - +
Flag-TGFBR2 - + +
MG-132 + + +
Input
IB:USP33

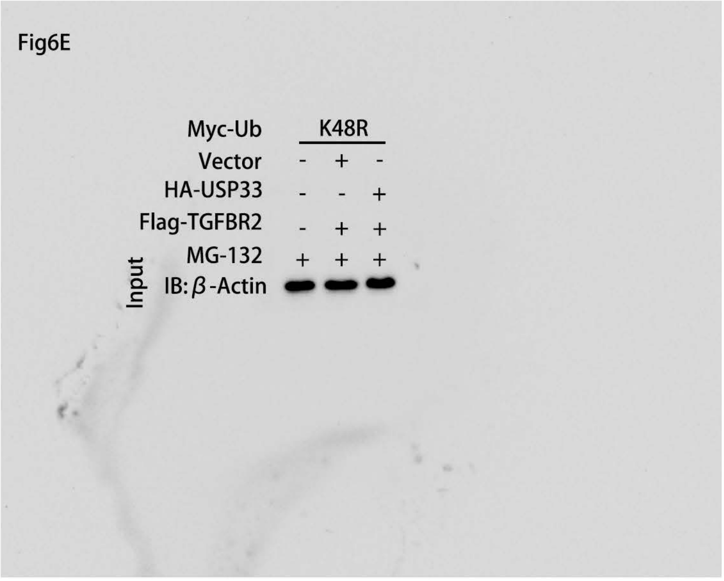
Fig6E
Myc-Ub K48R
Vector - + -
HA-USP33 - - +
Flag-TGFBR2 - + +
MG-132 + + +
Input
IB: β-Actin

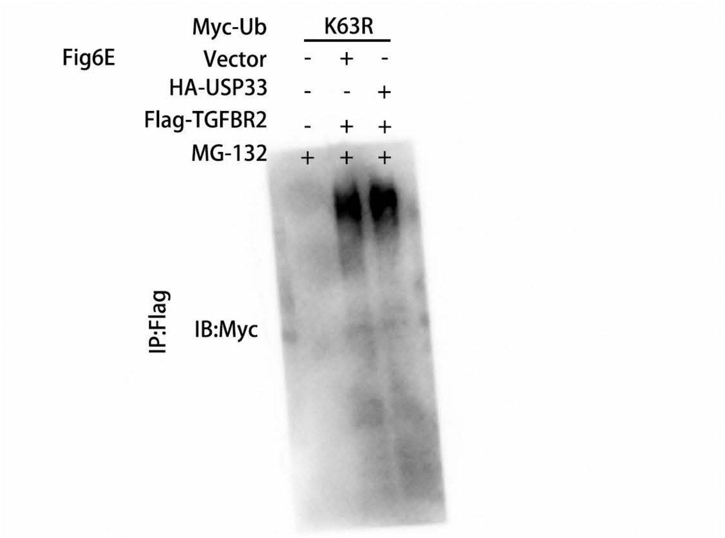
Fig6E
Myc-Ub K63R
Vector - + -
HA-USP33 - - +
Flag-TGFBR2 - + +
MG-132 + + +
IP:Flag
IB:Myc

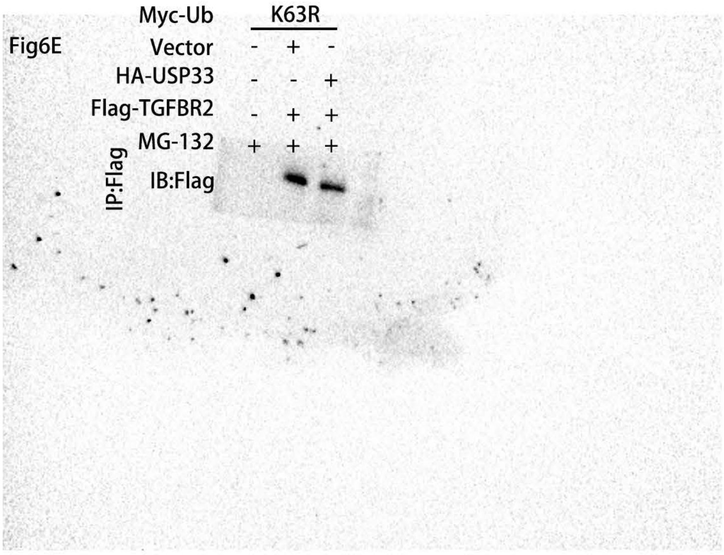
Fig6E
Myc-Ub K63R
Vector - + -
HA-USP33 - - +
Flag-TGFBR2 - + +
MG-132 + + +
IP:Flag
IB:Flag

Fig6E

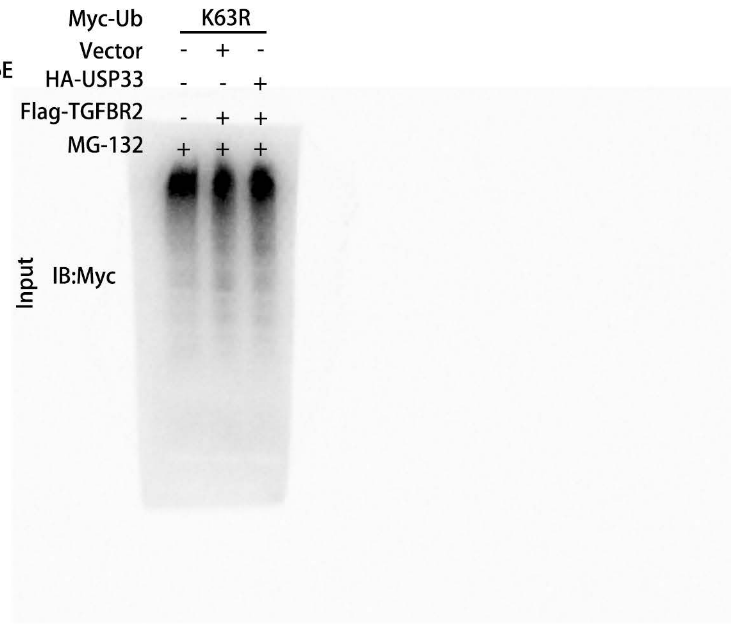

Fig6E

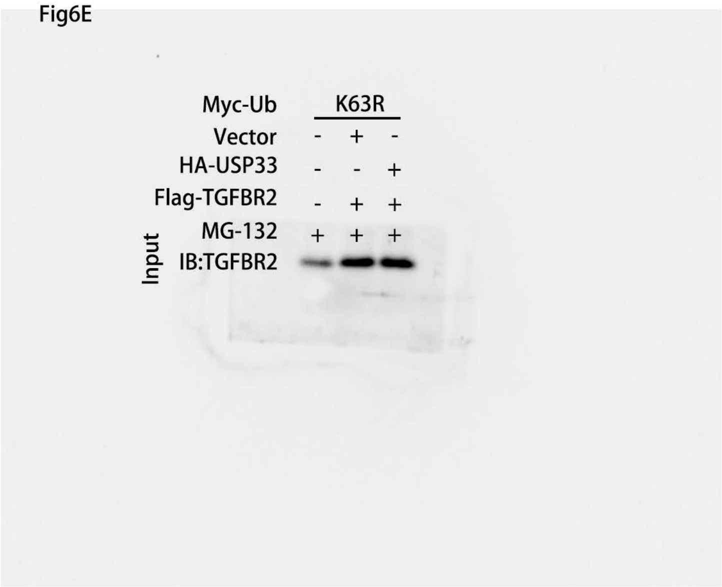

Fig6E

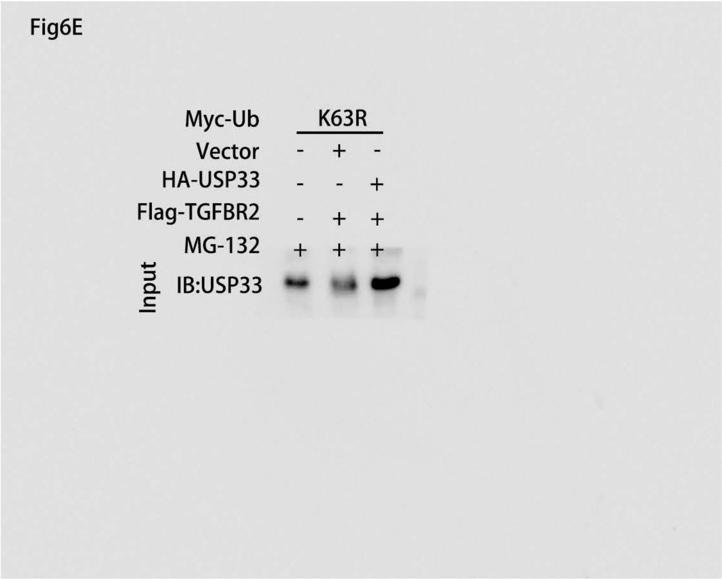

Fig6E

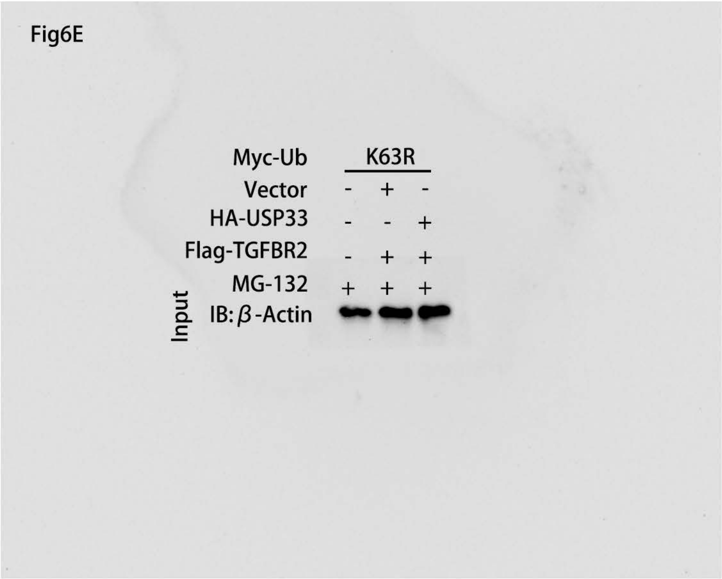

Fig6E

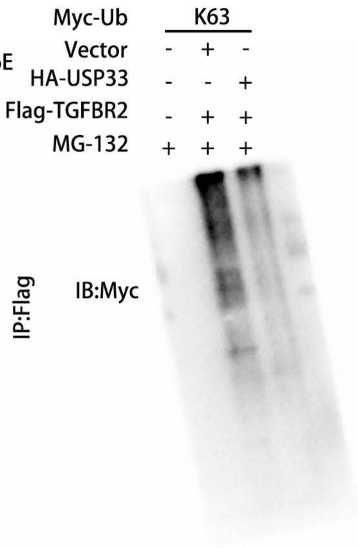

Fig6E

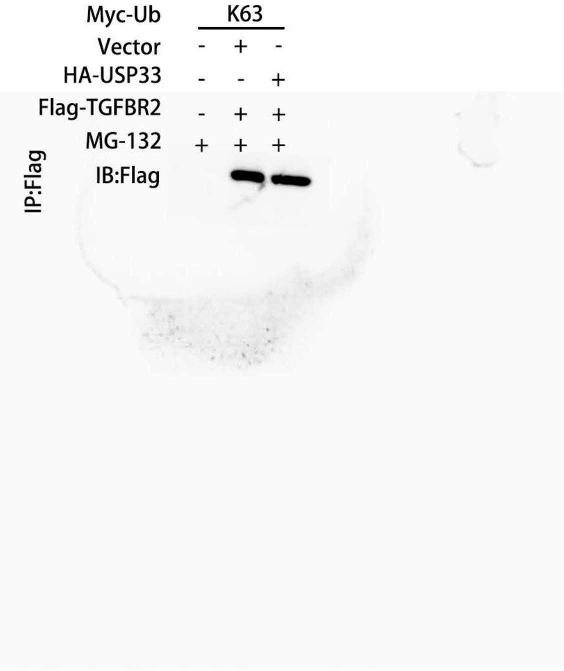

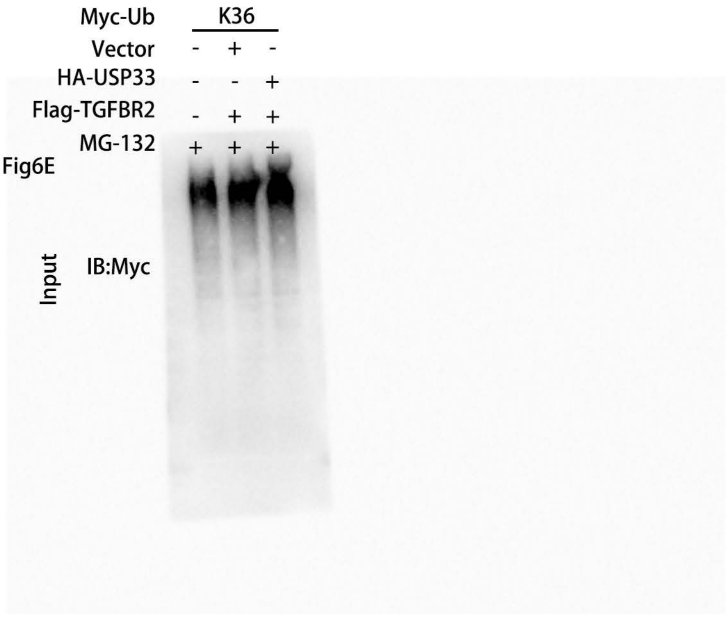
Myc-Ub K36
Vector - + -
HA-USP33 - - +
Flag-TGFBR2 - + +
MG-132 + + +
Fig6E
Input
IB:Myc

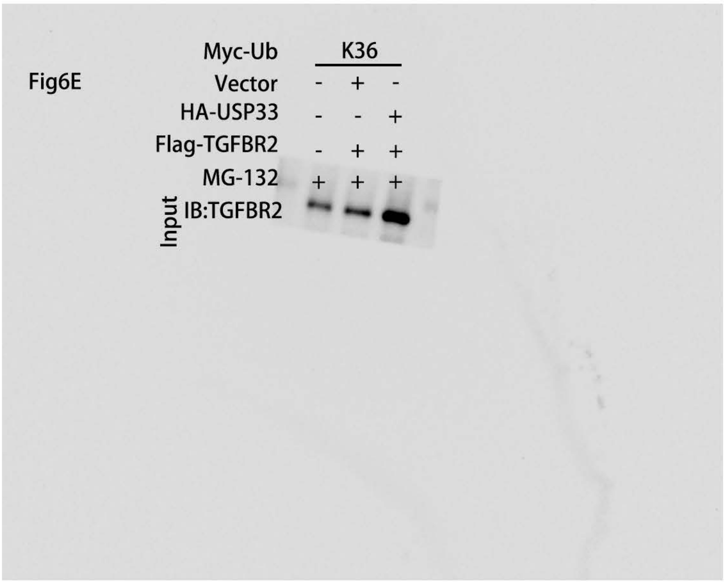
Fig6E
Myc-Ub K36
Vector - + -
HA-USP33 - - +
Flag-TGFBR2 - + +
MG-132 + + +
Input
IB:TGFBR2

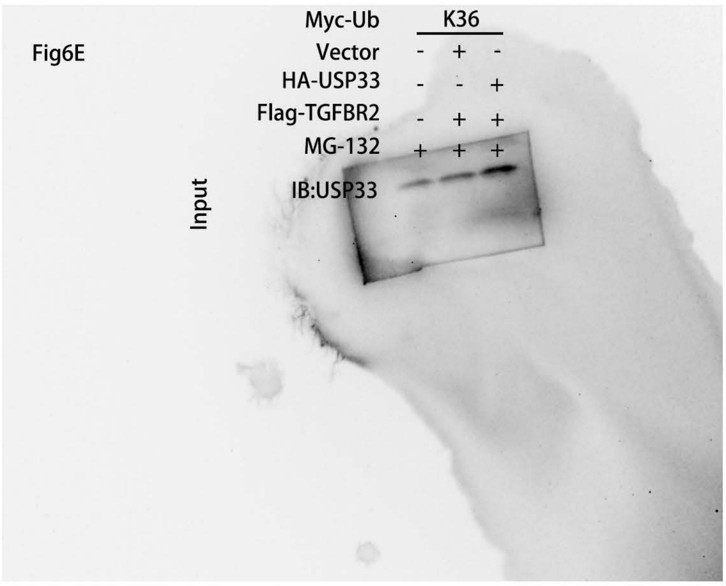
Fig6E
Myc-Ub K36
Vector - + -
HA-USP33 - - +
Flag-TGFBR2 - + +
MG-132 + + +
Input
IB:USP33

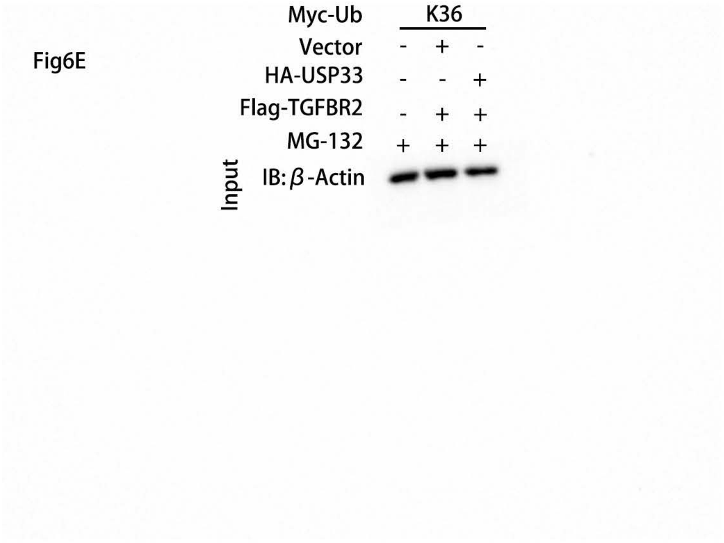
Fig6E
Myc-Ub K36
Vector - + -
HA-USP33 - - +
Flag-TGFBR2 - + +
MG-132 + + +
Input
IB: β -Actin

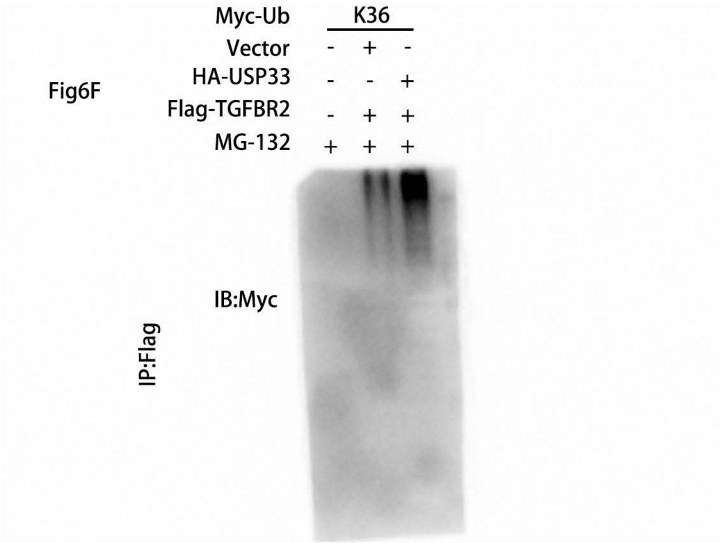
Fig6F
Myc-Ub K36
Vector - + -
HA-USP33 - - +
Flag-TGFBR2 - + +
MG-132 + + +
IB:Myc
IP:Flag

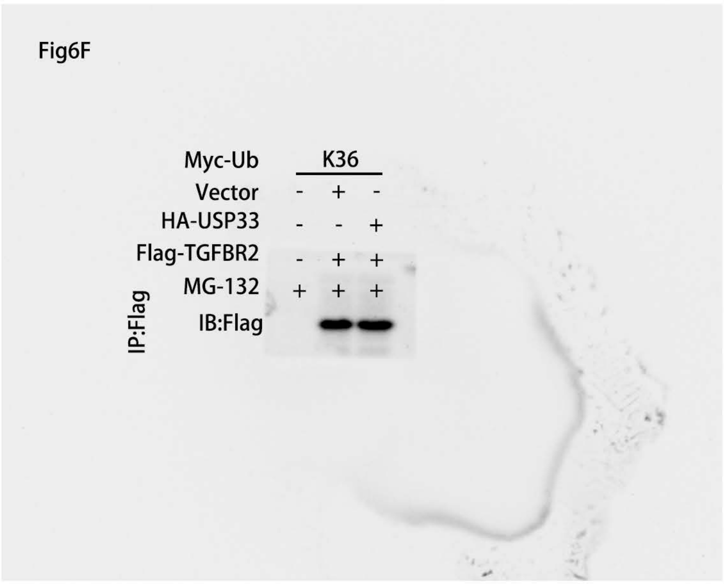
Fig6F
Myc-Ub K36
Vector - + -
HA-USP33 - - +
Flag-TGFBR2 - + +
MG-132 + + +
IP:Flag
IB:Flag

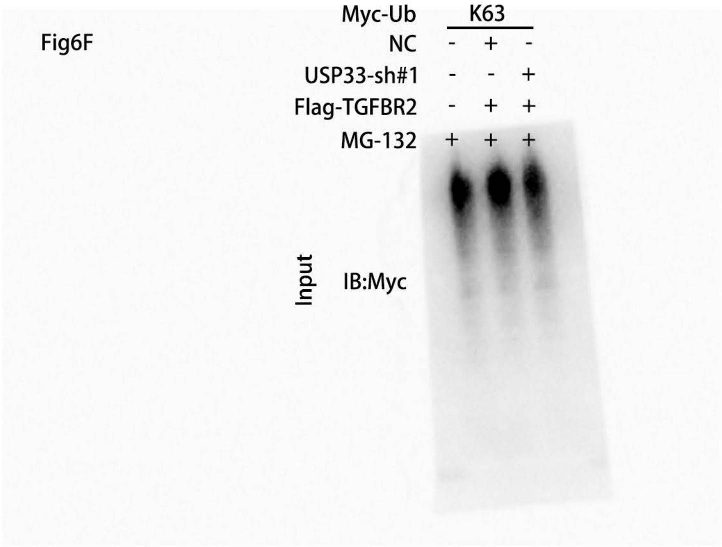
Fig6F
Myc-Ub
K63
NC - + -
USP33-sh#1 - - +
Flag-TGFBR2 - + +
MG-132 + + +
Input
IB:Myc

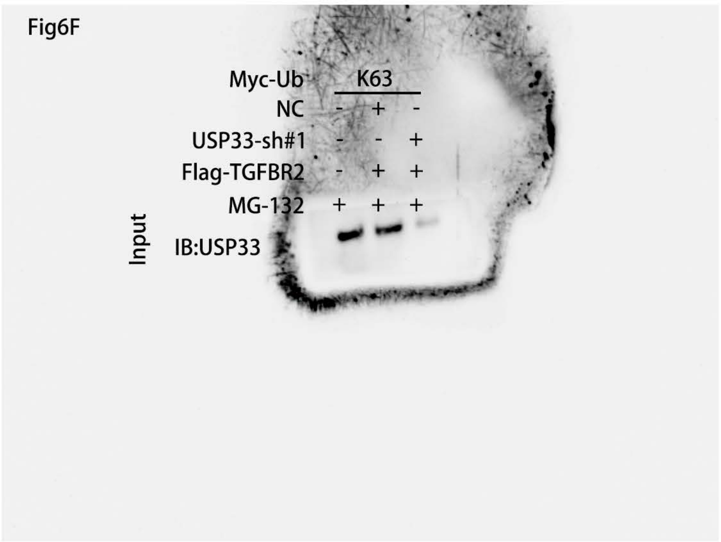
Fig6F
Myc-Ub
K63
NC - + -
USP33-sh#1 - - +
Flag-TGFBR2 - + +
MG-132 + + +
Input
IB:USP33

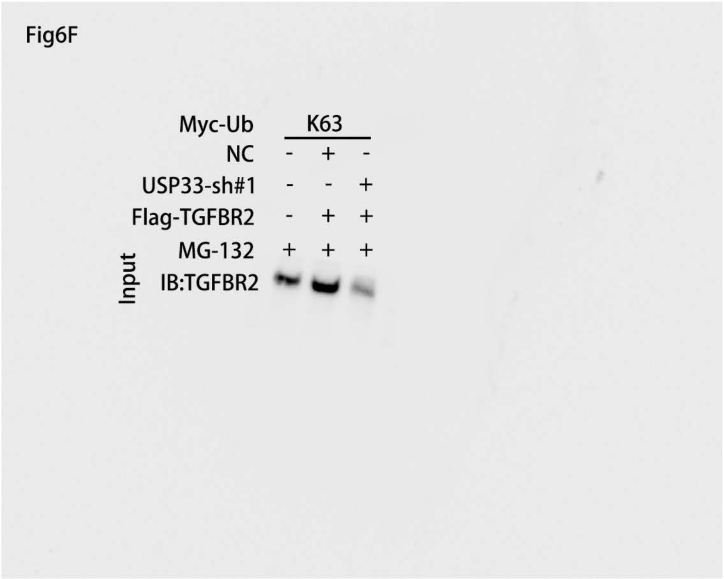
Fig6F
Myc-Ub
K63
NC - + -
USP33-sh#1 - - +
Flag-TGFBR2 - + +
MG-132 + + +
Input
IB:TGFBR2

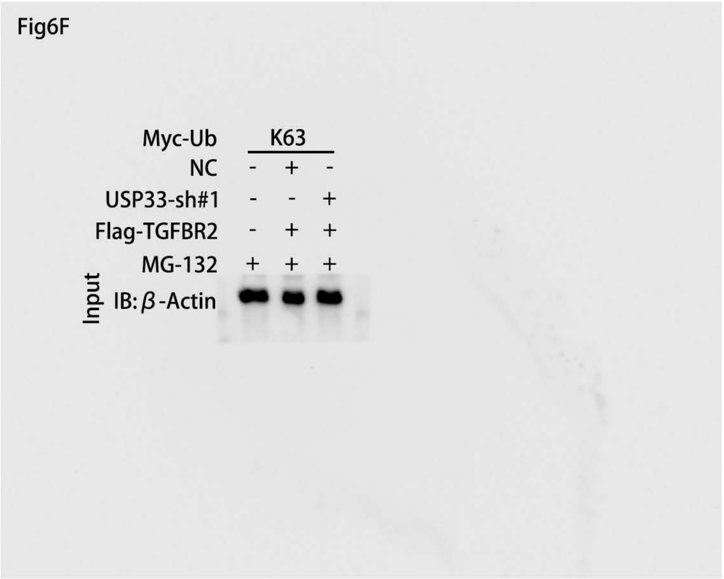
Fig6F
Myc-Ub
K63
NC - + -
USP33-sh#1 - - +
Flag-TGFBR2 - + +
MG-132 + + +
Input
IB: β -Actin

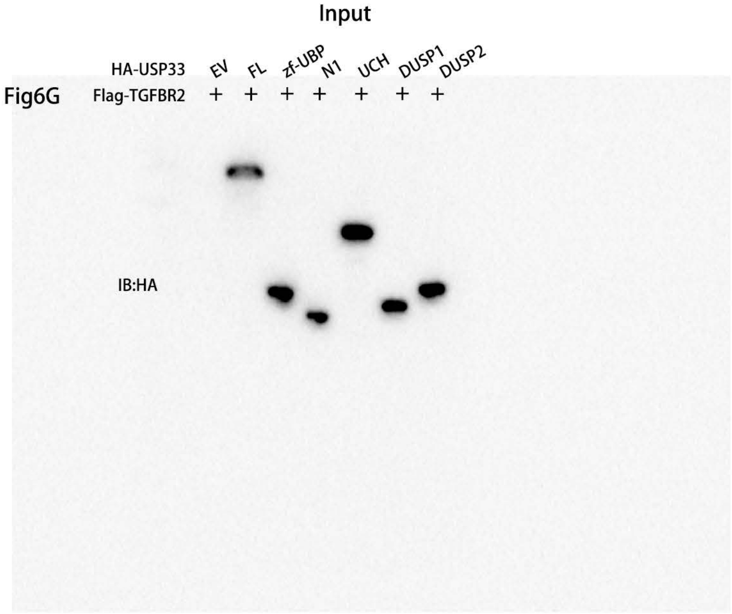
Input
HA-USP33
EV
FL
zf-UBP
N1
UCH
DUSP1
DUSP2
Fig6G
Flag-TGFBR2 + + + + + + +
IB:HA

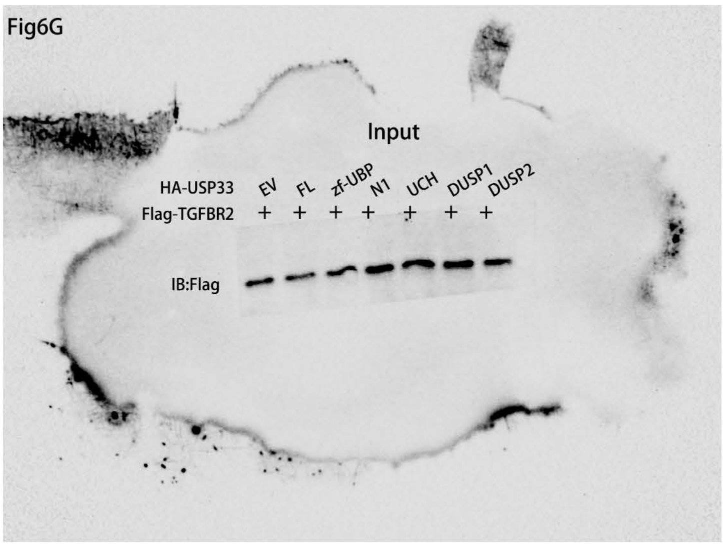
Fig6G
Input
HA-USP33
EV
FL
zf-UBP
N1
UCH
DUSP1
DUSP2
Flag-TGFBR2 + + + + + + +
IB:Flag

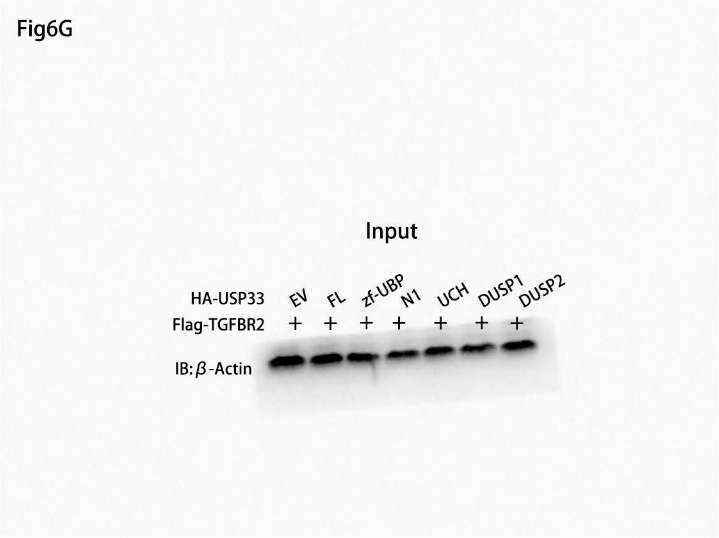
Fig6G
Input
HA-USP33 EV FL zf-UBP N1 UCH DUSP1 DUSP2
Flag-TGFBR2 + + + + + + +
IB: β-Actin

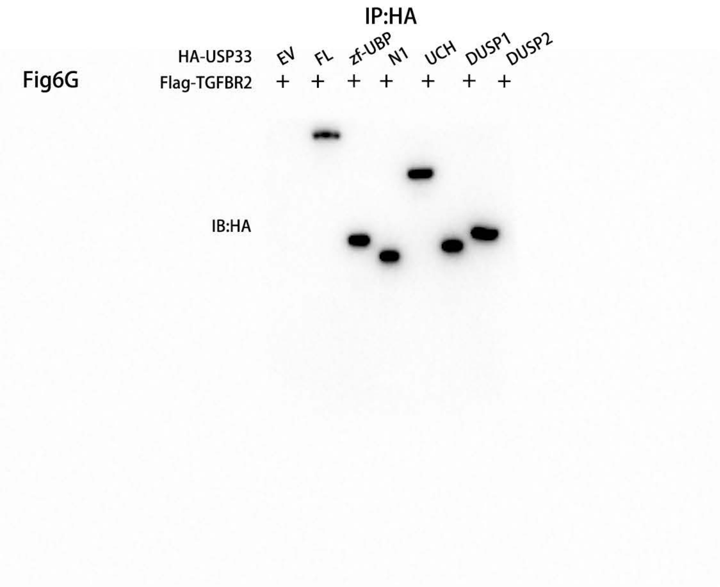
IP:HA
Fig6G
HA-USP33 EV FL zf-UBP N1 UCH DUSP1 DUSP2
Flag-TGFBR2 + + + + + + +
IB:HA

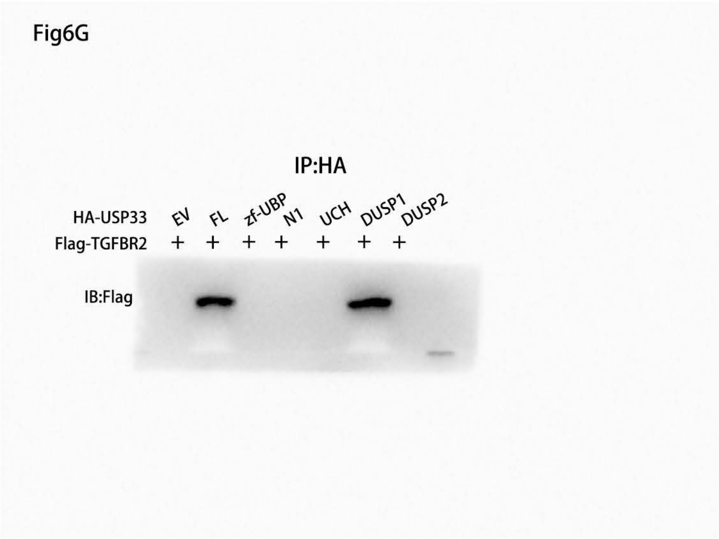
Fig6G
IP:HA
HA-USP33 EV FL zf-UBP N1 UCH DUSP1 DUSP2
Flag-TGFBR2 + + + + + + +
IB:Flag

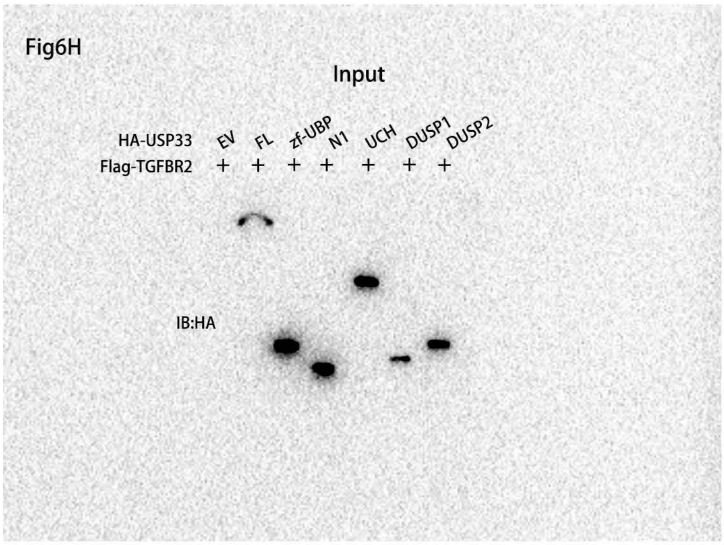
Fig6H
Input
HA-USP33 EV FL zf-UBP N1 UCH DUSP1 DUSP2
Flag-TGFBR2 + + + + + + +
IB:HA

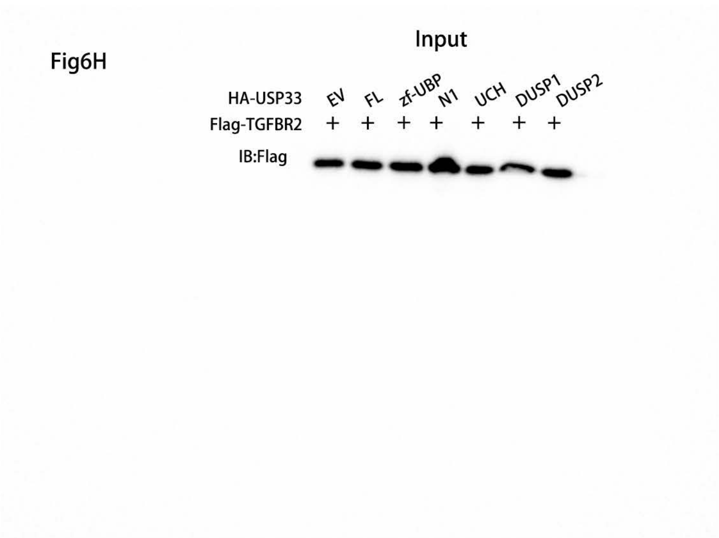
Fig6H
Input
HA-USP33 EV FL zf-UBP N1 UCH DUSP1 DUSP2
Flag-TGFBR2 + + + + + + +
IB:Flag

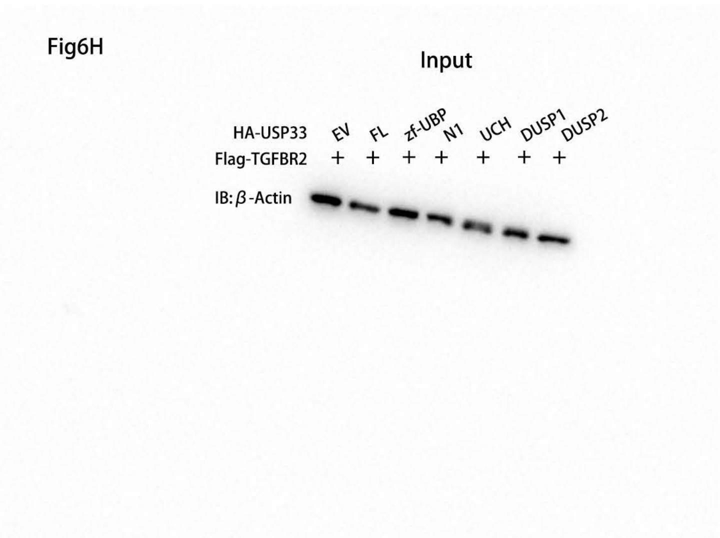
Fig6H
Input
HA-USP33 EV FL zf-UBP N1 UCH DUSP1 DUSP2
Flag-TGFBR2 + + + + + + +
IB: β-Actin

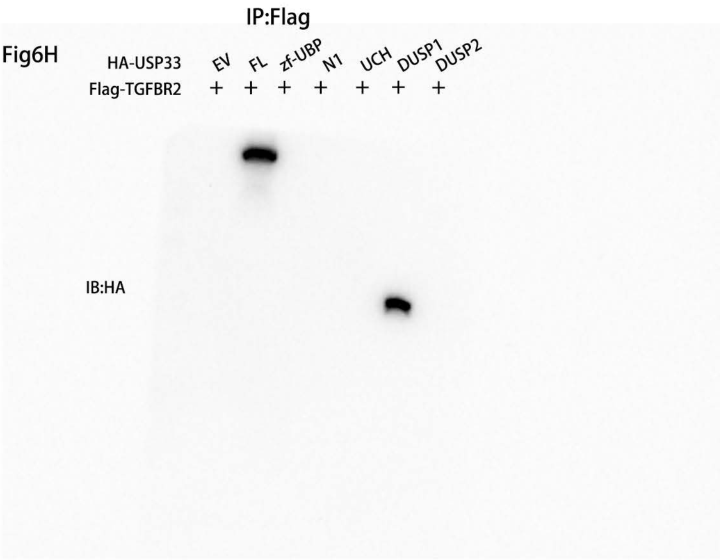
IP:Flag
Fig6H
HA-USP33 EV FL zf-UBP N1 UCH DUSP1 DUSP2
Flag-TGFBR2 + + + + + + +
IB:HA

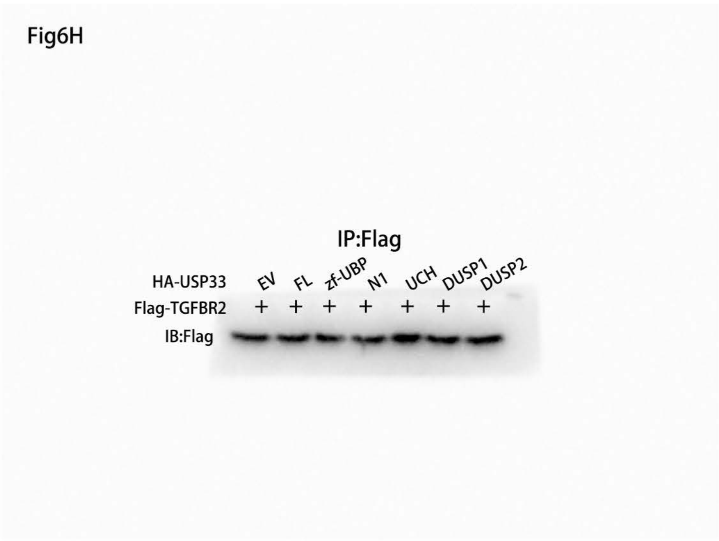
Fig6H
IP:Flag
HA-USP33 EV FL zf-UBP N1 UCH DUSP1 DUSP2
Flag-TGFBR2 + + + + + + +
IB:Flag

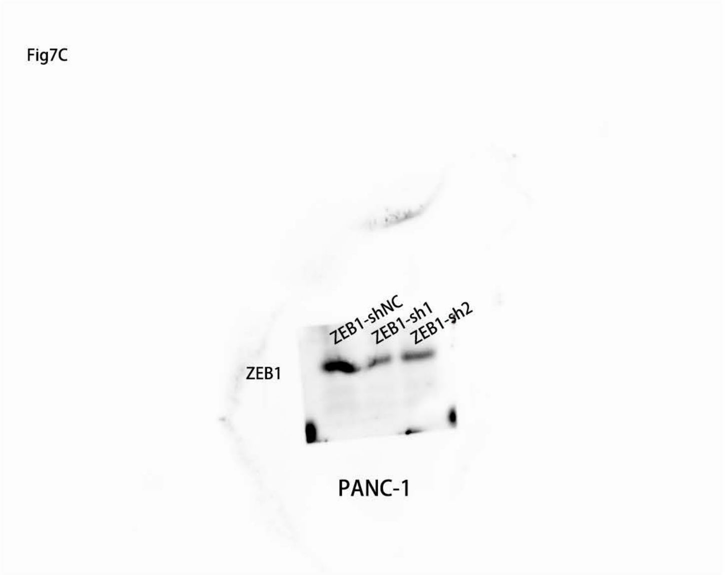
Fig7C
ZEB1-shNC ZEB1-sh1 ZEB1-sh2
ZEB1
PANC-1

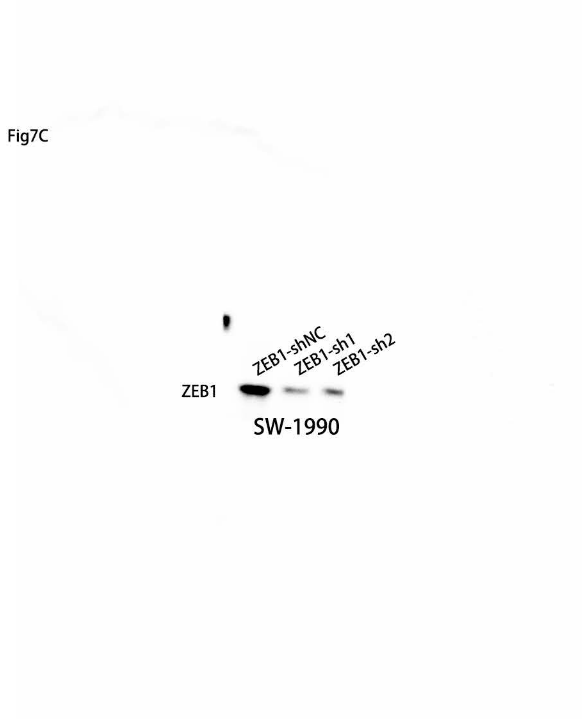
Fig7C
ZEB1-shNC ZEB1-sh1 ZEB1-sh2
ZEB1
SW-1990

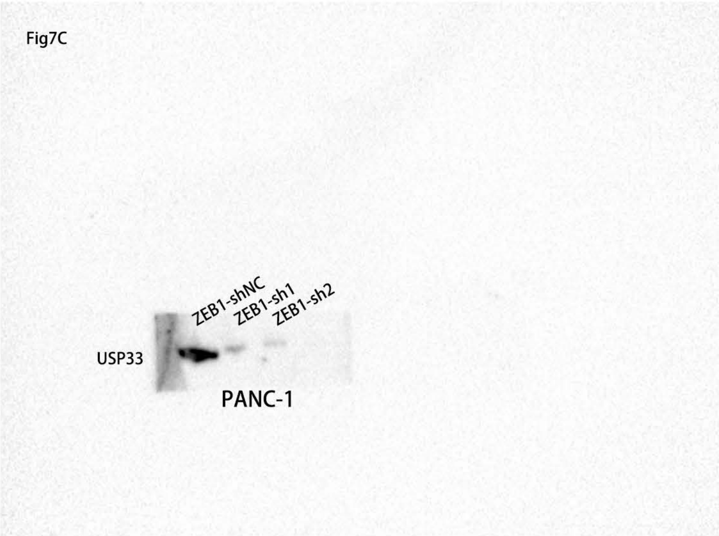
Fig7C
ZEB1-shNC ZEB1-sh1 ZEB1-sh2
USP33
PANC-1

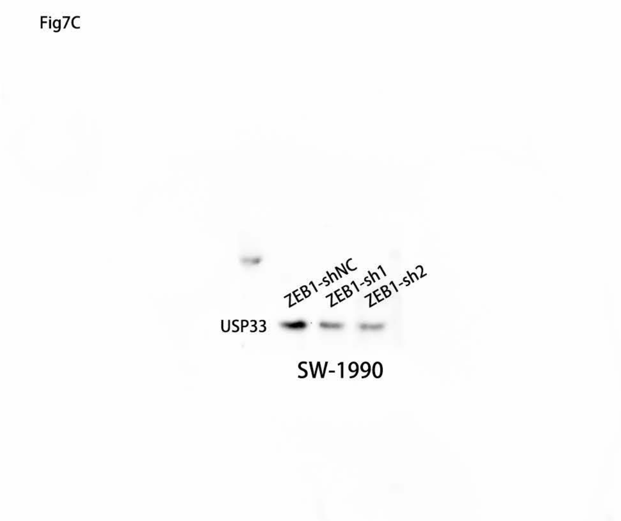
Fig7C
ZEB1-shNC ZEB1-sh1 ZEB1-sh2
USP33
SW-1990

Fig7C

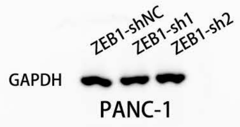

Fig7C

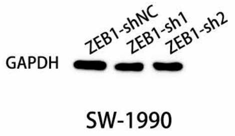

Fig7C

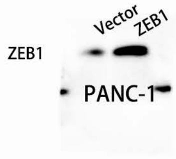

Fig7C

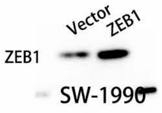

Fig7C

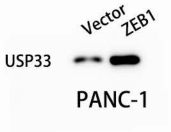

Fig7C

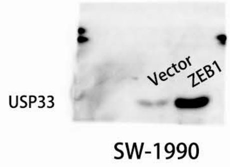

Fig7C

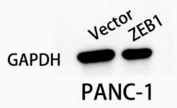

Fig7C

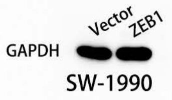

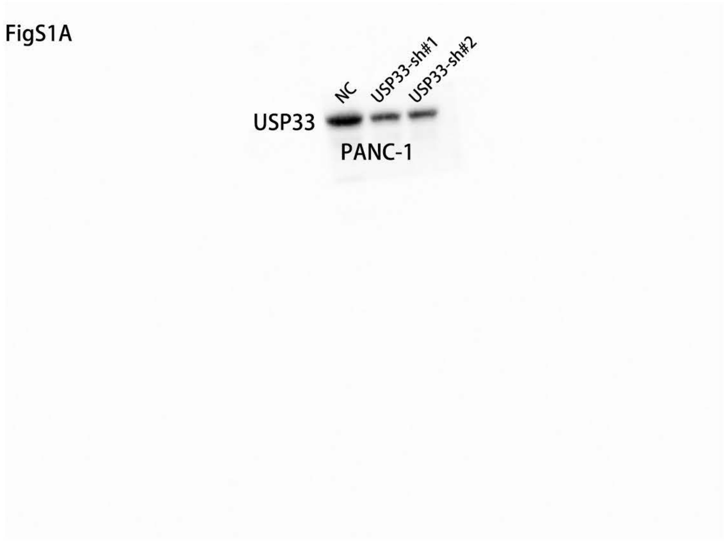
FigS1A
NC
USP33-sh#1
USP33-sh#2
USP33
PANC-1

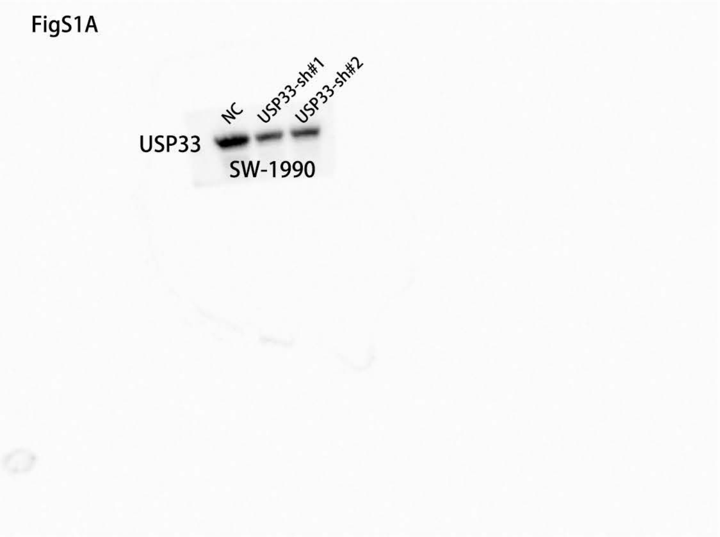
FigS1A
NC
USP33-sh#1
USP33-sh#2
USP33
SW-1990

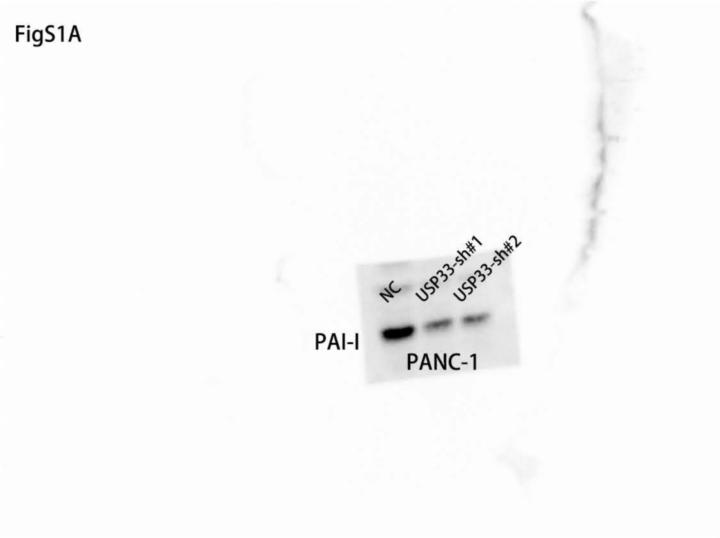
FigS1A
NC
USP33-sh#1
USP33-sh#2
PAI-I
PANC-1

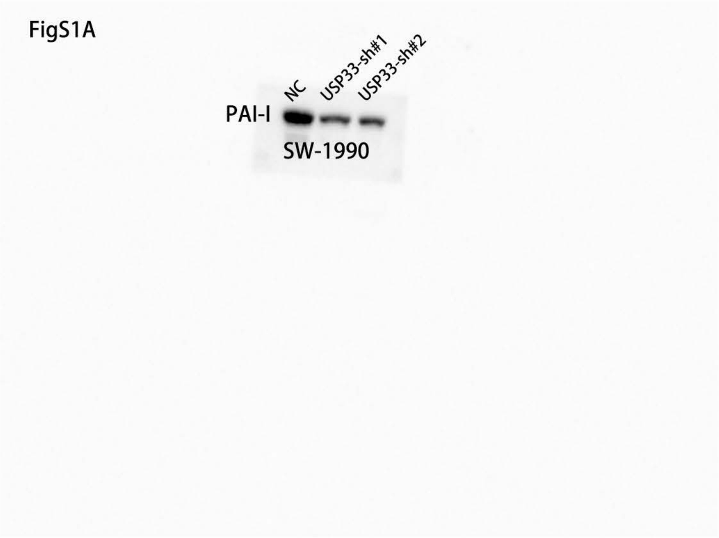
FigS1A
NC
USP33-sh#1
USP33-sh#2
PAI-I
SW-1990

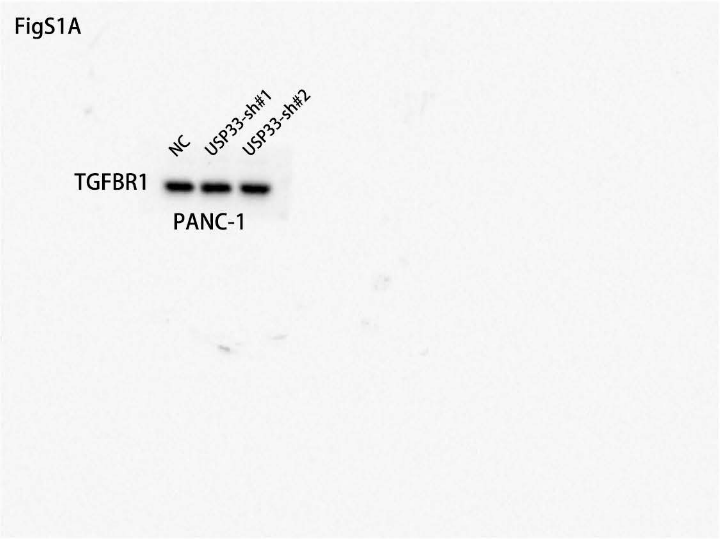
FigS1A
NC
USP33-sh#1
USP33-sh#2
TGFBR1
PANC-1

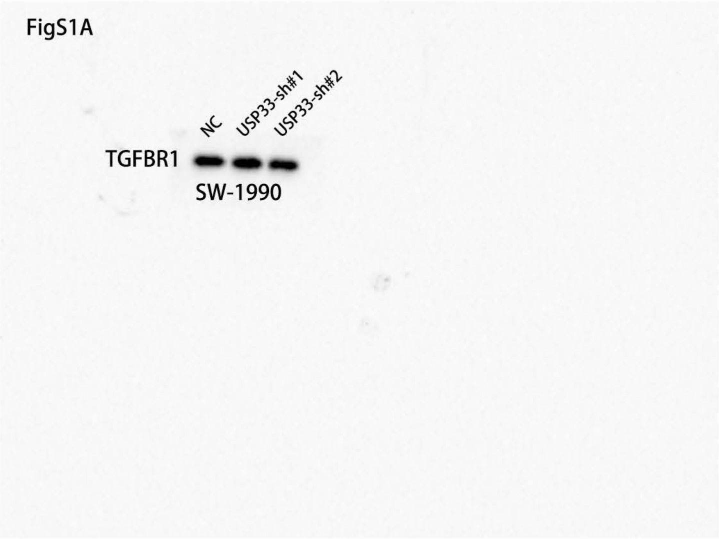
FigS1A
NC
USP33-sh#1
USP33-sh#2
TGFBR1
SW-1990

FigS1A

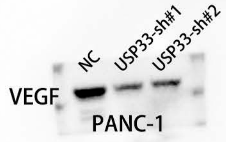

FigS1A

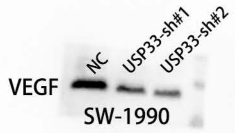

FigS1A

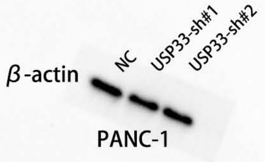

FigS1A

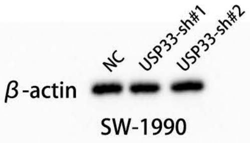

FigS1B

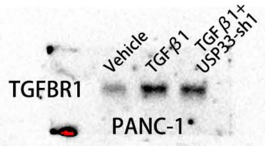

FigS1B

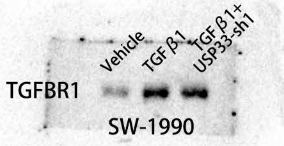

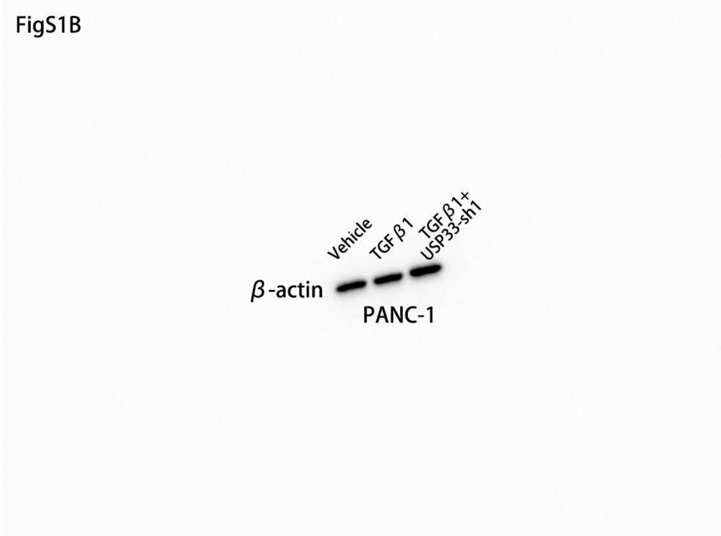
FigS1B
Vehicle
TGFβ1
TGFβ1+
USP33-sh1
β-actin
PANC-1

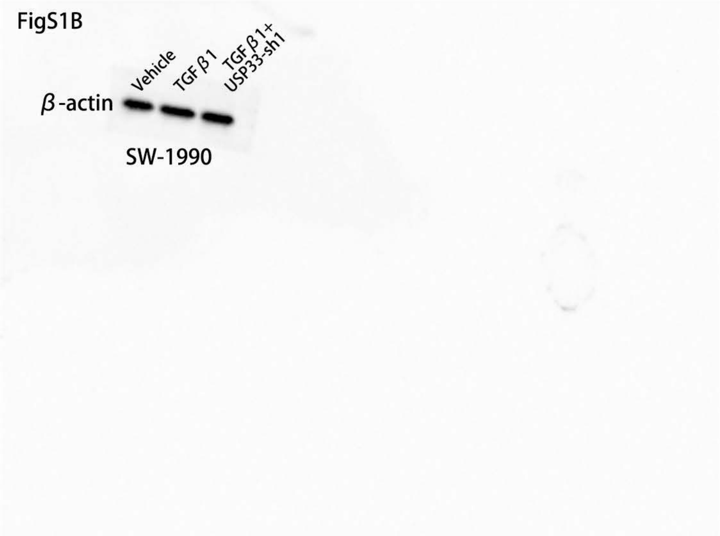
FigS1B
Vehicle
TGFβ1
TGFβ1+
USP33-sh1
β-actin
SW-1990

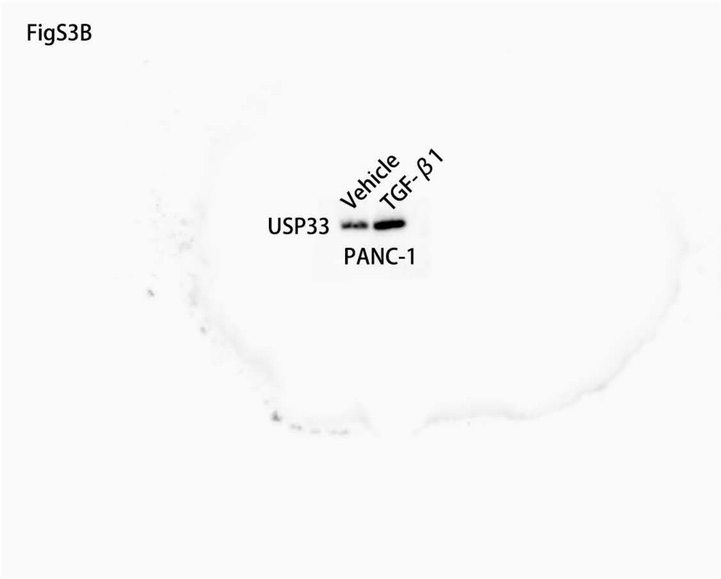
FigS3B
Vehicle
TGF-β1
USP33
PANC-1

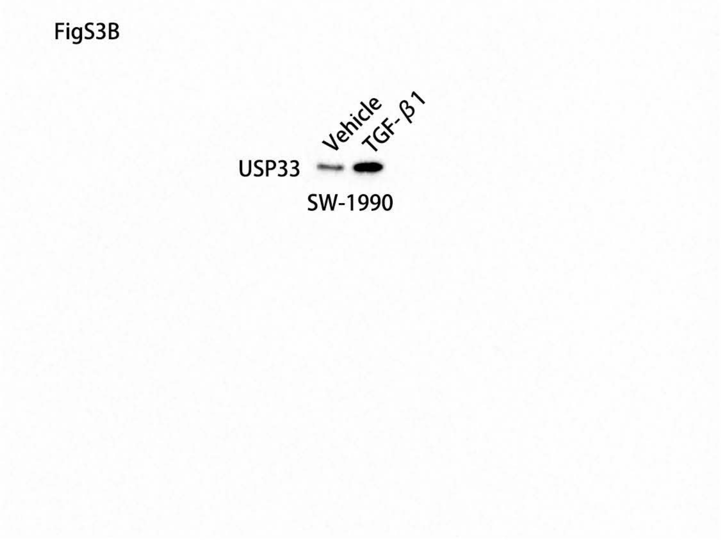
FigS3B
Vehicle
TGF-β1
USP33
SW-1990

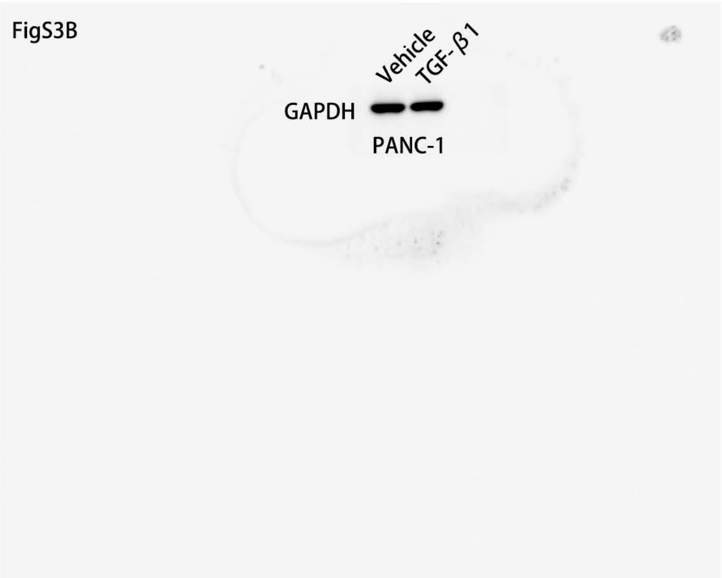
FigS3B
Vehicle
TGF-β1
GAPDH
PANC-1

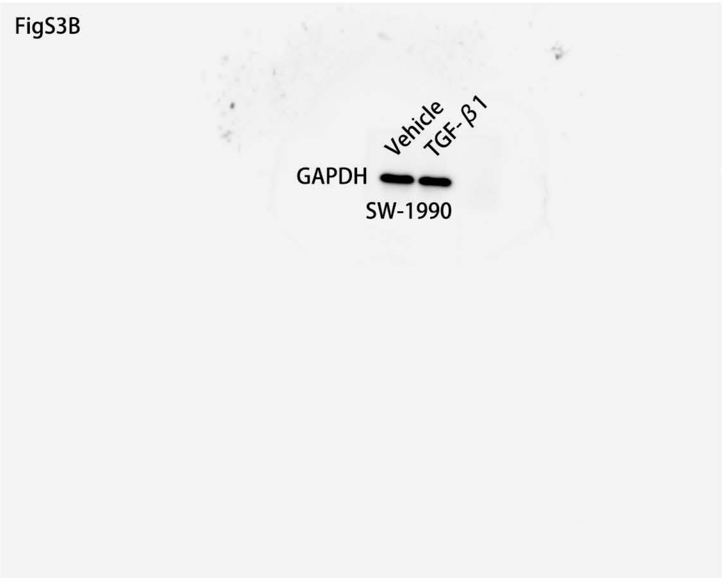
FigS3B
Vehicle
TGF-β1
GAPDH
SW-1990
